# *De novo* design of quasisymmetric two-component protein cages

**DOI:** 10.1038/s41586-026-10464-0

**Published:** 2026-05-20

**Authors:** Shunzhi Wang, Ying Xie, David Chmielewski, Connor Weidle, Tong Shu, Green Ahn, Ryan D. Kibler, Cindy Hernandez, Wei Chen, David Camilo Duran, Ann Carr, Asim K. Bera, Sangmin Lee, Justin Decarreau, Alex Kang, Evans Brackenbrough, Emily Joyce, Kejia Wu, Andrew J. Borst, Andrew Favor, Buwei Huang, Frank DiMaio, Liam Holt, David Baker

**Affiliations:** 1. Department of Biochemistry, University of Washington, Seattle, WA, USA.; 2. Institute for Protein Design, University of Washington, Seattle, WA, USA.; 3. Institute for Systems Genetics, New York University Langone Medical Center, New York, NY, USA.; 4. Department of Chemical Engineering, Pohang University of Science and Technology (POSTECH), Pohang, Republic of Korea.; 5. Molecular Engineering and Sciences Institute, University of Washington, Seattle, WA, USA.; 6. Howard Hughes Medical Institute, University of Washington, Seattle, WA, USA.

## Abstract

Quasisymmetric icosahedral viral capsids achieve larger sizes than possible with strictly symmetric icosahedra by tessellating pentagons and hexagons using a single subunit that adopts different conformations in symmetrically non-equivalent locations^1,2^. Recapitulating such quasisymmetric architectures through computational design is a considerable challenge in nanomaterials engineering. Here, we introduce a computational design strategy based on geometric frustration to generate two-component, quasisymmetric protein cages with customizable properties. We designed complementary trimeric and dimeric protein components that co-assemble into positively curved local hexagonal assemblies. Hexagonal lattices cannot tile spherical surfaces; instead, the components form closed sphere-like cage assemblies through incorporation of curvature inducing pentagonal defects, as evidenced by electron microscopy. By designing dimers that encode different local curvatures, we programmed cage dimensions ranging from 40 to over 200 nanometers in diameter and molecular weights from 2 to over 50 megadaltons, comparable to natural virus capsids. We further functionalized these large cages with additional protein domains to enable ribonucleoprotein cargo loading and cellular uptake. Fluorescently labeled cage assemblies expressed in mammalian cells function as rheological probes and cargo recruiters, enabling a systematic study of size-dependent cytoplasmic diffusion and protein localization. Thus, the quasi-symmetry which has long fascinated structural biologists can now be achieved by computational protein design, with immediate applications to biologics delivery and molecular cell biology.

While symmetry is widespread in Nature, it is not always perfectly respected, as illustrated by fullerenes^3^, quasicrystals^4^, and clathrin cages^5^. In the 1960s, Caspar and Klug showed that viruses generate larger capsids by relaxing perfect icosahedral symmetry among subunits with subtle deviations^1^. Such structural plasticity offers evolutionary advantages, enabling viruses to expand their capsid shells and accommodate larger genome cargos without a substantial increase in genetic encoding^2^. *De novo* protein design has generated well-defined self-assembling icosahedral structures^6,7^, which show considerable promise for vaccines^8,9^ and other applications^10,11^. However, the structures realized so far are largely limited to regular point group symmetries with discrete copy numbers, with the exception of pseudo-symmetric structures^12–14^ where multiple chemically distinct yet structurally similar chains occupy unique environments. Genetically encodable quasisymmetric structures, where a single sequence encodes multiple local conformations, remains a formidable design challenge, as multiple conformations must be accessible to a single sequence and the correct ones must be adopted during assembly to avoid large scale aggregation. Developing a strategy that intentionally breaks perfect symmetry in a programmable manner could unlock a vast design space for quasisymmetric structures, leading to structural and materials properties at organelle and subcellular scales (10–1000 nm), currently out of reach of current approaches.

We set out to develop a general strategy based on geometric frustration^15^ for designing self-limiting quasisymmetric particles. Instead of targeting protein monomers that inherently fold into multiple states, we focused on designing proteins capable of undergoing local conformational changes to accommodate a globally low energy architecture^16^. To drive spherical particle closure, we imposed positive curvature on hexagonal building blocks. Since hexagons alone cannot tile a spherical surface, pentagonal defects (+60° wedge disclinations) can emerge to relieve accumulated strain as these components co-assemble into closed spherical particles (total of 4π wedge disclination = 12 pentagons, Fig. 1a). We reasoned that symmetry breaking could arise in such a system if the energetic cost of monomer deformation, which determines the energy difference between pentameric and hexameric states, could be overcome by the gain in overall contact energy upon full particle closure (Extended Data Fig. 1). Such symmetry breaking to generate both pentagonal and hexagonal faces driven by the formation of energetically favorable interactions upon assembly closure also occurs in carbon fullerenes, but whereas in fullerenes the cost of the required C-C bond angle deformations is fully determined by carbon hybridization chemistry, in designed protein assemblies the interfacial bending angles can be continuously tuned to minimize local strain and stabilize the desired target structure.

We aimed to design two-component protein assemblies that approximate fullerene topology starting from a spherical hexagonal tessellation. In our design, each vertex is pre-positioned with an existing protein homotrimer designated as C3-A^17^, and we designed^18^ the edge to be a homodimer, designated the C2-B linker, which has a pre-specified curvature (Methods and Extended Data Fig. 2). To ensure precise assembly into the specified geometry, we employed a pre-validated heterodimer motif^19^ as the molecular interface between C3-A and C2-B. As shown in Fig. 1b, we placed the threefold axis of each C3-A at the vertices of an ideal hexagon of edge length ***l***. We then tilted each axis toward the global sixfold axis so that they lie on a cone of angle ***α***; ***l*** and ***α*** together set the target sphere size. Finally, we rotated each C3-A by an angle ***ω*** about its own threefold axis to control the relative orientation of the C2-B binding domains. We chose to design C2-B linkers with alpha-beta (α/β) folds for improved structural rigidity as compared to all alpha-helical folds^20^, to achieve the small deviations in cone angles required for different T number cages (Fig. 1c). The strong inter-subunit binding affinity (Kd ~ 10 nM^19^) enforces interface specificity and minimizes unintended off-target interactions, driving complete assembly closure to maximize binding interface pairing. This two-component system allows for straightforward separate purification of building blocks and subsequent *in vitro* experimental validation under various assembling conditions.

## Encoding cage size with hexagon curvature

**Fig. 2 |:**
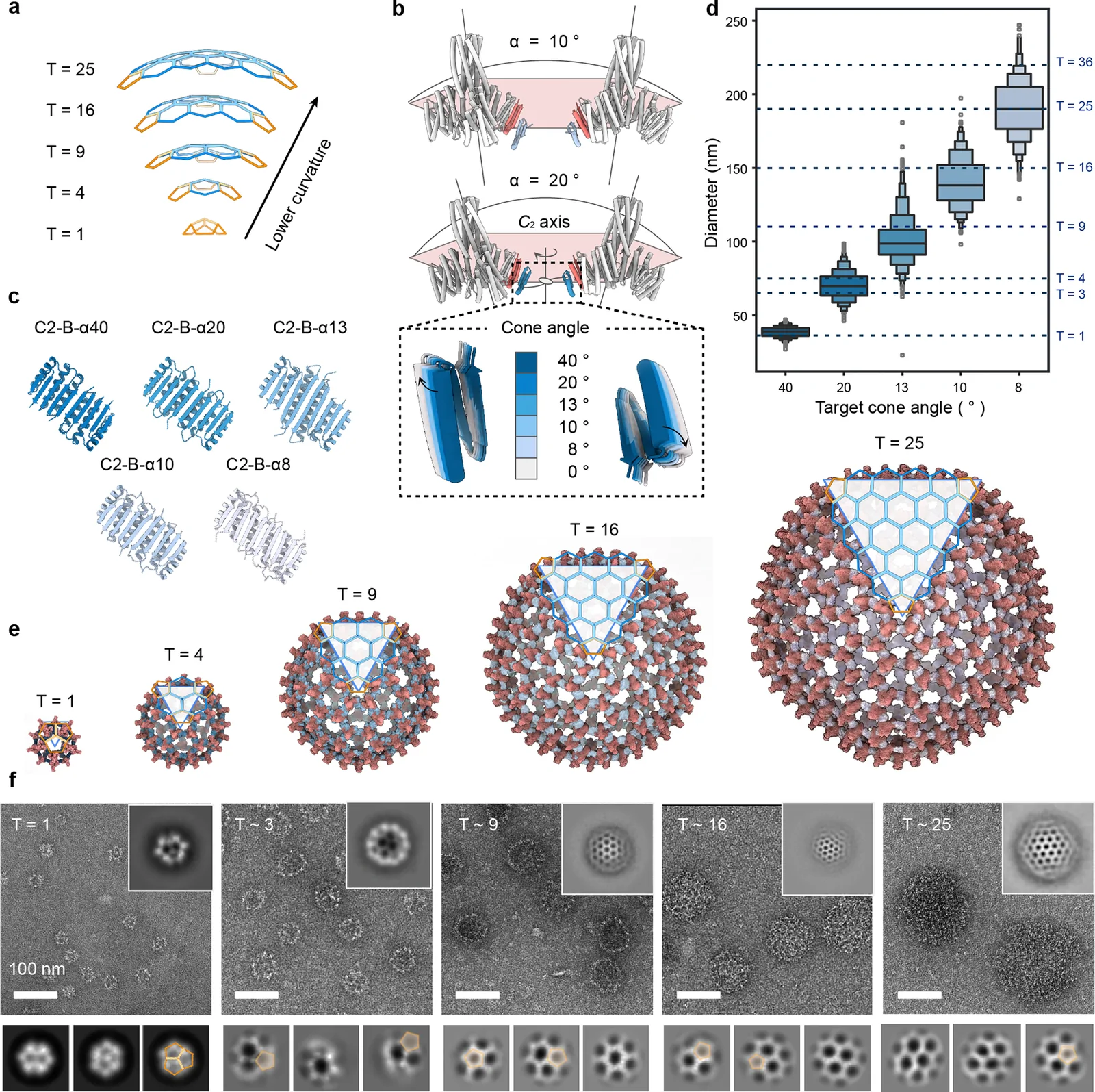
Cage size can be programmed through the curvature of designed dimeric linkers. **a**) Cartoon depiction of high-T cages illustrating reduced local curvature at individual hexagonal facets. **b**) Local curvature of the hexagonal building motif is controlled by a pre-specified cone angle, defined as the intersecting angle between two adjacent trimers linked by a single C2-B linker. A zoomed-in view highlights the close spatial proximity of C2-B interface placements targeting different cone angles (inset). **c**) Computational models of five designed dimeric linkers spanning a range of target cone angles. **d**) Particle size distributions were measured by nsEM and plotted as a function of cone angles (8° to 40°), shown as letter-value box plots. The center line indicates the median; Successive boxes outward represent the middle 50%, 75%, 87.5%, and 93.75% of the data, respectively (n=254/272/476/317/270 particles for 40/20/13/10/8 degree target cone angle). Individual outliers are indicated by dots. **e**) Cartoon depiction of ideal 2-component icosahedral cages with T numbers ranging from 1 to 25. **f**) Representative nsEM micrographs of the five designed dimeric linkers assembled with the shared trimeric component to form quasisymmetric cages (N = 3 independent experiments), with insets displaying selected 2D class averages. The bottom row shows nsEM 2D class averages, where pentagonal structures (highlighted in orange) are clearly visible across all designs.

We began by seeking to design a wide range of cage sizes by varying the cone angles. To target Goldberg icosahedra^21^ ranging from triangulation numbers T = 1 to T = 25, we designed a set of C2-B linkers with cone angles **α** ranging from 40° to 4° and fixed edge length **l** = 125 Å (Fig. 2a–c, Table S1). We used RFdiffusion^18^ to generate C2-symmetric homodimers that rigidly scaffold the pre-specified heterodimer binding motif at pre-specified geometry (Methods), and selected 36 for experimental testing. These 36 were expressed in *E. coli*; 32 could be purified via immobilized metal affinity chromatography (IMAC), and 26 eluted as a single peak at the expected elution volume on size exclusion chromatography (SEC, protein yield shown in Table S2). Upon mixing with C3-A at equal molar ratio (50 μM), we observed formation of larger assemblies as the major product fraction for 18 designs (Supplementary figure S2, Table S3). We then used negative-stain electron microscopy (nsEM) to screen for particle formation, and observed 8 designs show particle formation with low polydispersity (corresponding SEC profiles and dynamic light scattering data are shown in Supplementary figure S1). The remaining 10 designs formed particles with large size distributions, which we attributed to linker geometries unfavorable for particle closure. Analysis of particle diameters derived from nsEM measurement showed a clear negative correlation between designed cone angle and particle size distributions (Fig. 2d). The smaller cages tended to be more monodisperse than the larger cages. In contrast to the weak subunit-interactions which drive assembly of quasisymmetric clathrin cages^22^, the strong heterodimeric interface between C_3_-A and C2-B drives assembly in our designed cages, which could lead to greater heterogeneity due to kinetic trapping.

We set out to structurally characterize particles of different sizes using nsEM (Fig. 2d–f and Supplementary figure S3–4). We first examined a design with a cone angle **α** = 40°, which as intended generated a T=1 regular dodecahedron (~ 40nm), as evidenced by nsEM 2D classes which revealed predominantly pentagons. Reducing the cone angle to α = 30° yielded a broader distribution of polyhedral assemblies, comprising a mixture of T = 1 and larger T = 1–3 cages, consistent with a partial relaxation of curvature/closure constraints (Extended Data Fig. 3). Fitting the design model into the experimental 3D maps indicated that particle assembly based on heterodimer interactions was sufficient to bend the C3-A and C2-B linkers from the original hexagonal geometry to achieve strained five-membered ring closure. C2-B-α20 linkers, with a cone angle **α** = 20°, were found to generate spherical particles approximately 50–70nm in diameter (expected T number 3~4, Extended Data Fig. 4). C2-B-α13 linkers with **α** = 13° formed spherical particles about 100 nm in diameter. While we could not precisely determine the exact T number, the observed size range suggests a triangulation number of T ~ 9. A C2-B-α10 linker formed particles around 130 nm in diameter (T ~ 16), and three designed **α** = 8° linkers yielded particles approaching 200 nm in diameter, corresponding to T ~ 25 (Supplementary Fig. S5). The small angular deviation in designed C2-B linkers (C2-B-α40 vs. C2-B-α10) results in a significant change in cage triangulation number (T ~ 1 vs. T ~ 16, Supplementary Fig. S6). Due to the heterogeneous particle assembly we were not able to obtain well-defined 2D class averages for full particles with high T number (e.g. T > 9). The morphology of our assembled particles appeared rounded rather than faceted, consistent with the expected spherical shape of Goldberg polyhedra. Smaller cone angles generated roughly flat hexagonal lattices, and we observed irreversible aggregation upon mixing two components when targeted cone angles were smaller than 6° (Supplementary figure S7). Local structural analyses based on nsEM 2D class averages revealed the presence of pentagonal defects in all samples (Fig. 2f, bottom row and inset), confirming our hypothesis that cooperative deformation resulting in pentagonal defects drives particle closure.

## CryoEM characterization of T = 3 and high T cages

**Fig. 3 |:**
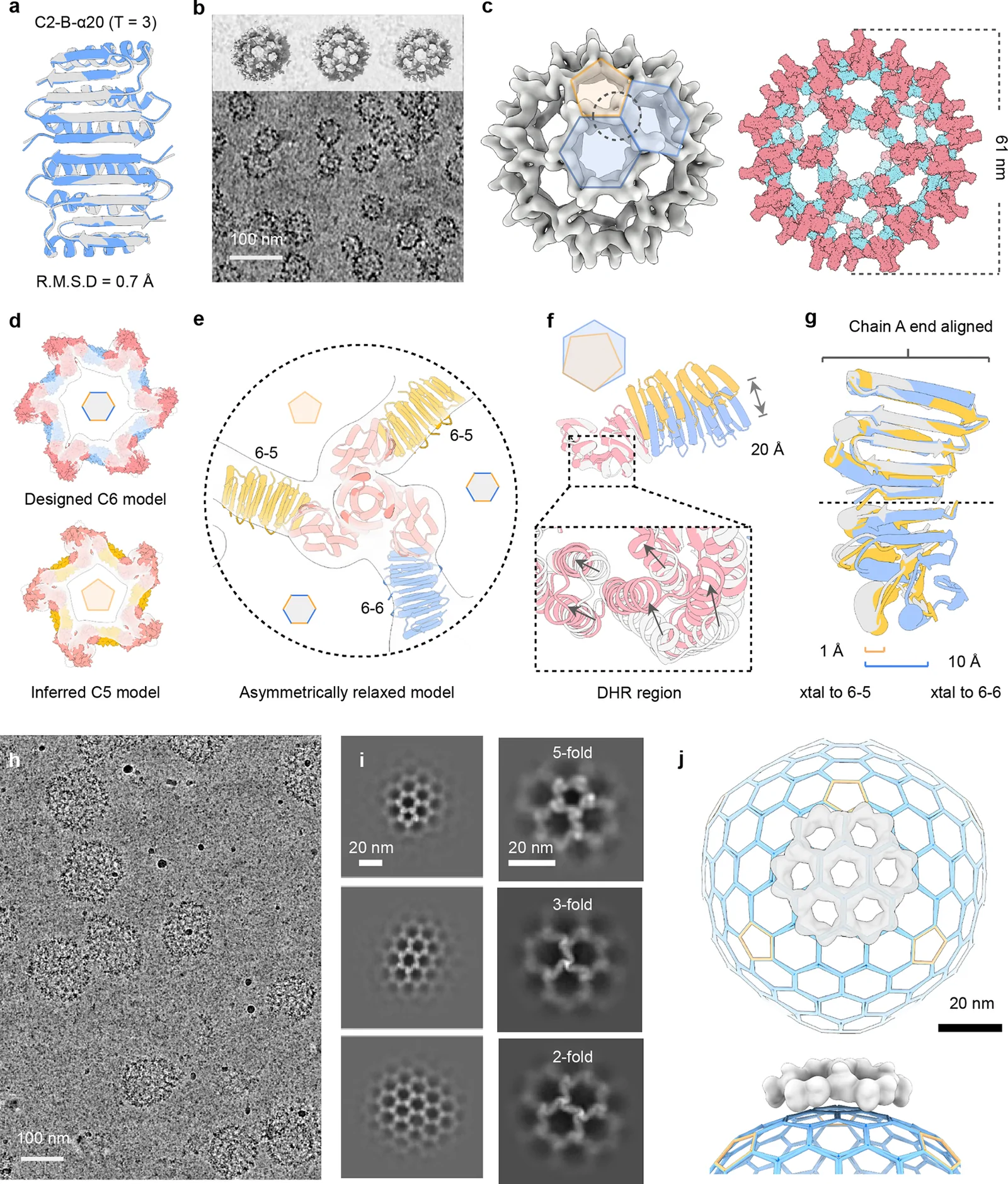
CryoEM characterization of *in vitro* assembled cages. **a**) The crystal structure of the designed C2-B-α20 linker for the T = 3 cage (blue) aligned to the design model (gray). **b**) CryoET tomogram Z-slice and three representative *ab initio* model reconstructions based on subtomogram averaged density without applied symmetry (inset). **c**) A cryoEM 3D density map at 20 Å resolution refined with applied Icosahedral symmetry (left) and corresponding relaxed full particle model (right). The dihedral angle between two highlighted hexagonal facets 6–6 is 138°, and between a hexagonal facet and pentagonal facet 6–5 is 142°, respectively. **d**) Overlay of experimental map (gray) with hexagonal ring design model (top) and calculated pentameric ring model (bottom), respectively. **e**) A zoomed in view of one C3-A homotrimer and three adjacent C2-B-α20 linkers relaxed into experimental density. C3 symmetry is broken to form asymmetric local structures as a result of particle assembly. **f**) Alignment of 6–6 (grey and blue) to 6–5 (pink and orange) conformations showing a gap distance of 20 Å between the termini of two dimers. A zoomed in view of the DHR region (inset). **g**) Comparison of 6–6 (blue) to 6–5 (orange) conformations of the C2-B-α20 linker aligned to one end of its crystal structure (gray). **h**) CryoEM micrograph (successfully repeated for N = 3 experiments) and **i**) local 2D class averages of T ~ 16 cage (C2-B-α10). **j**) Top and side view of 3D reconstructed maps corresponding to curved local hexagons.

We sought to characterize *in vitro* assembled cages with cryo-EM to understand the structural basis of quasisymmetric particle closure. We chose to first analyze the cage design targeting T = 3, the simplest Goldberg icosahedron, which is structurally equivalent to a C60 fullerene and has the general shape of a soccer ball with 12 pentagonal and 20 hexagonal facets. There are a total of 60 vertices (C3-A) and 90 edges (C2-B). We obtained a crystal structure at 2.1 Å of the designed C2-B-α20 linker, which closely agrees with the design model (Root mean square deviation, RMSD = 0.7 Å; Fig. 3a). For the complete cage, we obtained both single-particle (Supplementary figure S8) and cryo-electron tomography (cryoET, Fig. 3b) data. Subtomogram 3D classification of all particles into three classes without symmetry averaging revealed half complete cages with arrangements of pentagon and hexagon assemblies matching the expected T = 3 architecture (Fig. 3b, top). Further refinement of the largest class with icosahedral symmetry applied resulted in a ~30 Å map with T = 3 icosahedral structure (Fig. 3c, left). Starting from the designed hexamer model, we used Rosetta with a make_symmdef_file^23^ to build a C5 complex preserving the subunit-subunit interface in the design. These rings were docked into the density using Chimera, the asymmetric unit (a full C3-A and 3 adjacent C2-B-α20 linkers) was extracted, and Rosetta relax with density constraints^24^ was used to refine the full assembly into the cryoEM density (PDB ID: 9OP9). This (Methods, Fig. 3c, right) resulted in a full assembly model with a truncated icosahedron shape (diameter = 61 nm) and the C3-A helical bundles pointing outward bridged by C2-B-α20 dimers). The extracted experimental hexagonal and pentagonal structural motifs are both slightly smaller than the designed hexagonal model and computationally derived pentagonal model, respectively (Fig. 3d). Within the particle, the homo-trimeric protein C3-A and the associated C2-B-α20 partner assume two distinct local environments: two adjacent hexagons (denoted as 6–6) or hexagon-pentagon (denoted as 6–5). This splitting causes the homo-trimeric protein C3-A to break its original C3 symmetry and become an asymmetric trimer (Fig. 3e and Supplementary figure S9). The termini of the two different conformations (6–6 to 6–5) differ in position by 20 Å when the components are aligned on the trimeric core (Fig. 3f). The designed helical repeat (DHR) region of the trimer (residue 90–234) undergoes the most significant conformational shift, contributing to symmetry breaking. We identified two conformational states for the relaxed C2-B-α20 linkers with different bending angles and broken C2 symmetry, corresponding to the hexagonal 6–6 and the pentagonal 6–5 ring configurations, respectively. The 6–5 conformation is closer to the crystal structure of the linker alone (RMSD = 1 Å) than the 6–6 conformation (RMSD = 2 Å) (Fig. 3g).

To investigate larger cages with higher triangulation numbers, a single particle cryo-EM dataset was collected for the *in vitro* assembled T ~ 16 cages (C2-B-α10). Most particles were observed to be spherical and closed, with no apparent gaps (Fig. 3h). The variations in particle sizes and shapes suggest cage assemblies do not strictly follow Goldberg icosahedral architecture. The observed structural heterogeneity prevents averaging full particles; instead we combine 2D class averages and local region reconstructions to characterize the curved lattices and the spatial distribution of pentagonal assemblies. In 2D class averages, we observed hexagonal array patterns and confirmed the presence of pentagonal assemblies (Fig. 3i). We did not observe two pentagons within the same 2D class regardless of particle box size, and hence the spatial distribution of pentagons is most likely to be random and not evenly distributed (Supplementary figure S10). We also identified heptagonal defects adjacent to pentagonal rings and distorted hexagons, both of which have been observed as topological defects in large fullerenes^25^, highlighting the flexibility of the lattice to accommodate both positive and negative curvatures (Extended Data Fig. 5 and Supplementary discussion). The reconstructed 3D map of a curved hexagon and its nearest neighbors show good agreement with the corresponding region of an idealized T = 16 icosahedron (Fig. 3j). Electron cryo-tomography of the *in vitro* assembled T ~ 25 cage reveals a near-spherical morphology with a hollow interior. (Supplementary figure S11).

## Modular cargo loading and cellular uptake

**Fig. 4 |:**
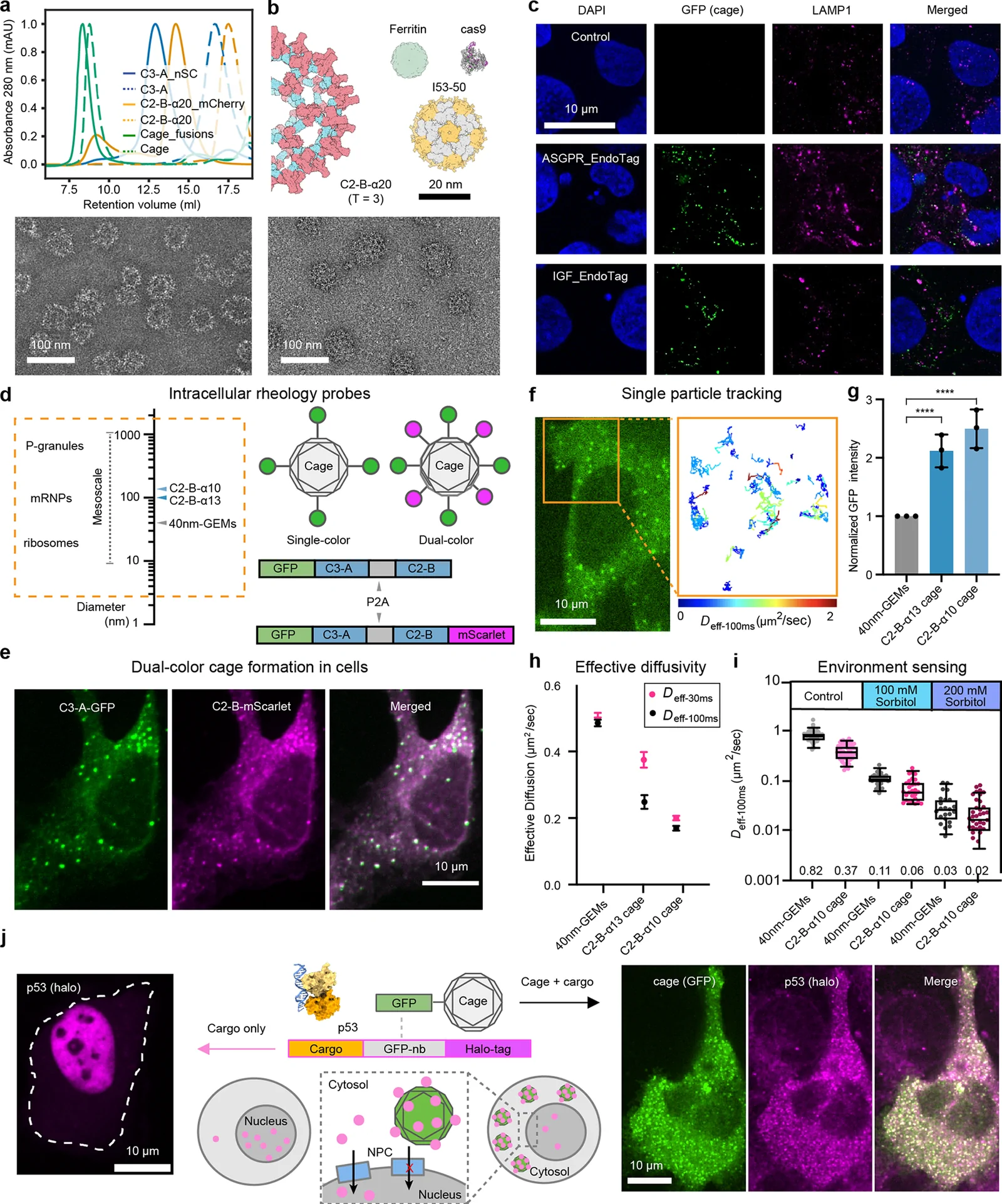
Cage assembly *in vitro* and in living cells for cargo packaging and rheological probes. **a**) SEC elution profiles (top) and nsEM micrograph (bottom) comparing cages assembled *in vitro* from the original building blocks (dashed lines) and from fusion-protein building blocks (solid lines). **b**) Bulky RNP cargo (Cas9) do not fit in most natural (ferritin) and *de novo* cages (I53–50); nsEM shows GFP-nanobody-functionalized T=3 cages encapsulating GFP-Cas9 (bottom, the reported morphology was observed across multiple fields of view from the same preparation). **c**) Confocal fluorescence images of fixed HEP3B cells incubated with AS_EndoTag and IGF_EndoTag functionalized GFP-cages. **d**) Probe sizes relative to cellular length scales (left) and schematic of single- and dual-color cages (right). **e**) Dual-color cages (GFP/mScarlet) in U2OS cells. **f**) Single particle tracking of single-color C2-B-α10 cages (GFP) in U2OS cell. Individual particle trajectories (zoomed in view) were color coded based on effective diffusivity coefficient (*D*_eff-100ms_). **g**) Normalized probe intensity in fixed U2OS cells (Data are presented as mean ± standard deviation from N=3 independent experiments, 40nm-GEMs: n=329; C2-B-α13 cages: n=358; C2-B-α10 cages: n=235; One-way ANOVA with Dunnett’s multiple comparisons test was used). **h**) The effective diffusivity at 30 ms (*D*_eff-30ms_) and 100 ms (*D*_eff-100ms_) decreases with probe size of the particles. (40nm-GEMs: n=3358 particles; C2-B-α13 cages: n=769 particles; C2-B-α10 cages: n=3157 particles, Median ± 95% confidence interval). **i**) *D*_eff-100ms_ for 40nm-GEMs and C2-B-α10 cages upon 10-minute sorbitol treatment. (Each datapoint represents one cell, N = 3 independent experiments, 40nm-GEMs: control n=55, 100 mM sorbitol n=31, 200 mM sorbitol n=23; C2-B-α10 cage: control n=56, 100 mM sorbitol n=28, 200 m=M sorbitol n=34) Box bounds: the 25th/75th percentiles; whiskers: min/max; and center lines: median. **j**) Cargo-loading schematic and nuclear import test based on confocal imaging: p53 localizes to the nucleus alone (left) but is retained in the cytosol when bound to cages (right). NsEM micrographs and confocal images are representative; key observations were reproduced in at least three independent experiments (**a**, **c**, **e**, **f**, **j**). All p values were determined from Dunn’s multiple comparison test (**g**). Scale Bars: 100 nm (**a**, **b**) or 10 μm (**c**, **e, f, j**).

Modular functionalization of *de novo* cages could provide control over their interactions with biological systems and enable downstream biomedical applications. Both C3-A and C2-B of our designed cages have exposed termini that support straightforward genetic fusions with natural and *de novo* proteins, for example fluorescent proteins^26^, *de novo* peptide binders^27^, nanobodies^28^, HaloTag^29^, SpyCatcher/SpyTag^30^, and ApoE3^31^. C3-A-nSpyCatcher and C2-B-α20-nMCherry fusion proteins were expressed in *E. coli* and purified by SEC as soluble oligomers with expected molecular weight (Fig. 4a, Supplementary figure S12). Robust *in vitro* assembly of functionalized C3-A and C2-B resulted in monodisperse cages at high yield as indicated by SEC elution profiles and nsEM (Supplementary figure S13 and Extended Data Fig. 6). Due to their internal cavity, these designed cages have the potential to encapsulate large cargoes such as ribonucleoprotein gene editors, which remains challenging for both natural viral particles and previous designs. As a step in this direction, we prepared T = 3 cages with C3-A and C2-B linkers fused to an anti-GFP nanobody^27^ (C2-B-α20_nGFPnb). Upon mixing with GFP-labeled Cas9, we observed efficient cargo encapsulation, as evidenced by co-elution of the GFP signal with the nanobody-functionalized cages using SEC (Supplementary Fig. S14). NsEM analysis revealed no appreciable morphological changes following encapsulation (Fig. 4b).

Next, we sought to functionalize cages with designed binding proteins^31^ that target different endocytic cell surface receptors to facilitate uptake. We genetically fused the N-terminus of different C2-B-α20 linkers with GFP and three *de novo* designed proteins that promote endocytosis (EndoTags) through the asialoglycoprotein receptor (ASGPR) the insulin-like growth factor 2 receptor (IGF2R), and sortillin^32^ (ASGPR_EndoTag, IGF_EndoTag, and Sort_EndoTag). *In vitro* assembled cages were purified by SEC as a single peak with no appreciable change in morphology according to nsEM. Using confocal microscopy, we observed intracellular puncta for all three types of EndoTag cage conjugates following incubation with HEP3B cells for 24 hours (Fig. 4c). Quantitative uptake was assessed by flow cytometry (Supplementary Fig. S15). T = 3 cages (diameter ~ 60 nm) functionalized with ASGPR and Sortilin binding proteins show cellular uptake, as evidenced by partial co-localization of GFP signals with the Lysosomal tracker LAMP1 signal, whereas GFP-labeled (no Endotags) cages showed minimal internalization.

We also evaluated cellular uptake of AS_EndoTag and Sort_EndoTag functionalized cages of three different sizes (60 nm: C2-B-α20, 80 nm: C2-B-α10, and 100 nm: C2-B-α8). Compared with unfunctionalized C2-B linkers, GFP- and EndoTag-fused constructs consistently formed smaller assemblies under identical monomer and salt concentrations, which we attribute to charge repulsion from the highly anionic GFP domains. Flow-cytometry and time-course measurements (Supplementary Fig. S15) revealed that uptake increased over 24 h for all constructs, but the influence of cage diameter was observed to be receptor-dependent: sortilin-targeted cages favored larger diameters under the tested conditions, while ASGPR-targeted cages showed higher uptake for smaller cages. Receptor engagement thus impacts cellular internalization, with size effects context-dependent rather than universal.

## Intracellular cage assembly as rheological probes

Single particle tracking nanorheology is a powerful strategy to probe the complex mesoscale physical properties of intracellular environments. These properties are dynamic and vary in a non-linear fashion across length- and time-scales^33^. Passive rheological probes have been developed based on genetically encoded multimeric nanoparticles (GEMs) from natural scaffolds, including *Pyrococcus furiosus* (40nm-GEMs^34^) and *Quasibacillus thermotolerans* (50nm-GEMs^35^); however, it has been difficult to make larger nanoparticles. Condensates such as μNS particles (50–150 nm) have been used to investigate longer length scales^36^, where distinct cellular biophysical properties emerge^37^, but these condensates do not have a defined size. Therefore, there is an acute need for well-defined rheological probes at the ~100 nm length scale relevant to cell biological processes such as vesicle transport. Given their tunable size and robust assembly, we hypothesized that our quasisymmetric protein cages could provide an orthogonal and genetically encoded approach for expanding the nanorheology toolbox to study physical properties in mammalian cells.

To study cytoplasmic physical properties at the mesoscale^34^, we prepared two types of fluorescently labeled cages (Fig. 4d) and expressed them in human bone osteosarcoma epithelial cells (U2OS cells). To confirm that the observed fluorescence signals originated from particles formed with the designed two components instead of random aggregates, we co-expressed the C3-A protein fused with GFP and the C2-B-α10 linker protein fused to the mScarlet fluorescence protein^38^ (Fig. 4e, Supplementary Fig. S16, and Table S4). Using identical TrackMate analysis across samples within each condition, our C3-A-nGFP_C2-B-α10 cages displayed narrow Gaussian particle-intensity distributions, slightly wider than that of 40 nm GEMs, in contrast to the broad, heterogeneous distributions characteristic of synDrops (Supplementary Fig. S17). When assembled in test tubes, the C2-B-α10 construct forms 140 nm cages (T ~ 16) with a high particle assembly yield. 60% of the GFP-labeled particles were colocalized with mScarlet-labeled particles (particle positions in two channels were within 180 nm), whereas almost no GFP puncta were in close proximity with randomized spot positions, consistent with the cages being assembled from the designed two components.

We performed single particle tracking analyses on C2-B-α13 and C2-B-α10 single-color cages generated by fusing GFP to the N terminus of the scaffold C3-A protein and co-expressing with two different C2-B linkers (Fig. 4f). As compared to previously developed 40nm-GEMs^33^ (40 nm genetically encoded multimeric nanoparticles), an increase in GFP mean intensity was observed for both cages, consistent with their larger number of subunits (Fig. 4g, and Supplementary video 1). We analyzed particle trajectories that were continuously monitored for more than 10 frames and deduced the effective diffusivity at 30 ms (*D*_eff-30ms_) and 100 ms (*D*_eff-100ms_) from mean square displacements (MSDs). We found that the effective diffusivity of different particles negatively correlates with their *in silico* designed size, making them useful rheological tools to infer physical parameters in mammalian cells. All three particle types exhibited higher effective diffusivity at 30 ms than at 100 ms, indicating time-scale-dependent physical constraints (Fig. 4h). This effect was more pronounced for cages designed in this study, suggesting greater intracellular constraints at larger length scales. To explore whether these probes can effectively sense cellular environmental changes, we used sorbitol treatment to acutely reduce intracellular water content, decrease cell volume, and thereby increase macromolecular crowding. The diffusivity *D*_eff-100ms_ of both 40nm-GEMs and C2-B-α10 cages decreased with increasing sorbitol concentrations, indicating that their diffusion is sensitive to physical property perturbations (Fig. 4i), as would be expected for a rheological tracer. We found that the particles can also form in *Saccharomyces cerevisiae* (yeast, Supplementary figure S18), indicating that they may be useful in diverse organisms.

We reasoned that tagging intracellular cargo molecules of interest could enable their recruitment into our quasisymmetric cages expressed and assembled in cells. To explore this, we created a stable cell line that expresses the C2-B-α10 cages fused to GFP. As a model system, we genetically fused the C-terminus of the p53 protein with a anti-GFP nanobody and a HaloTag^29^, and transiently transfected U2OS cells with the plasmid (Fig. 4j). The assembled C2-B-α10 cages effectively recruited the p53 protein through GFP binding to the GFP nanobody in U2OS cells, as evidenced by pronounced co-localization of the GFP labeled cage and the halo tagged p53 upon addition of the Halo ligand. p53 is normally localized in the nucleus (Fig. 4j, left), but upon cage binding, the constructs become too large to enter the nucleus or invade mitotic chromatin and are thus restricted to the cytosol^39^ (Fig. 4j, right). Introducing interface-disrupting C3-A mutations (L267K/R) impaired cage assembly and reduced p53 translocation, confirming that cargo redistribution requires intact cage formation (Supplementary Fig. S19). Thus, our designed multivalent protein cages can be loaded with cargo in cells; this could be useful for delivery applications, and for controlling subcellular protein localization.

## Discussion

**Extended Data Fig.1 |:**
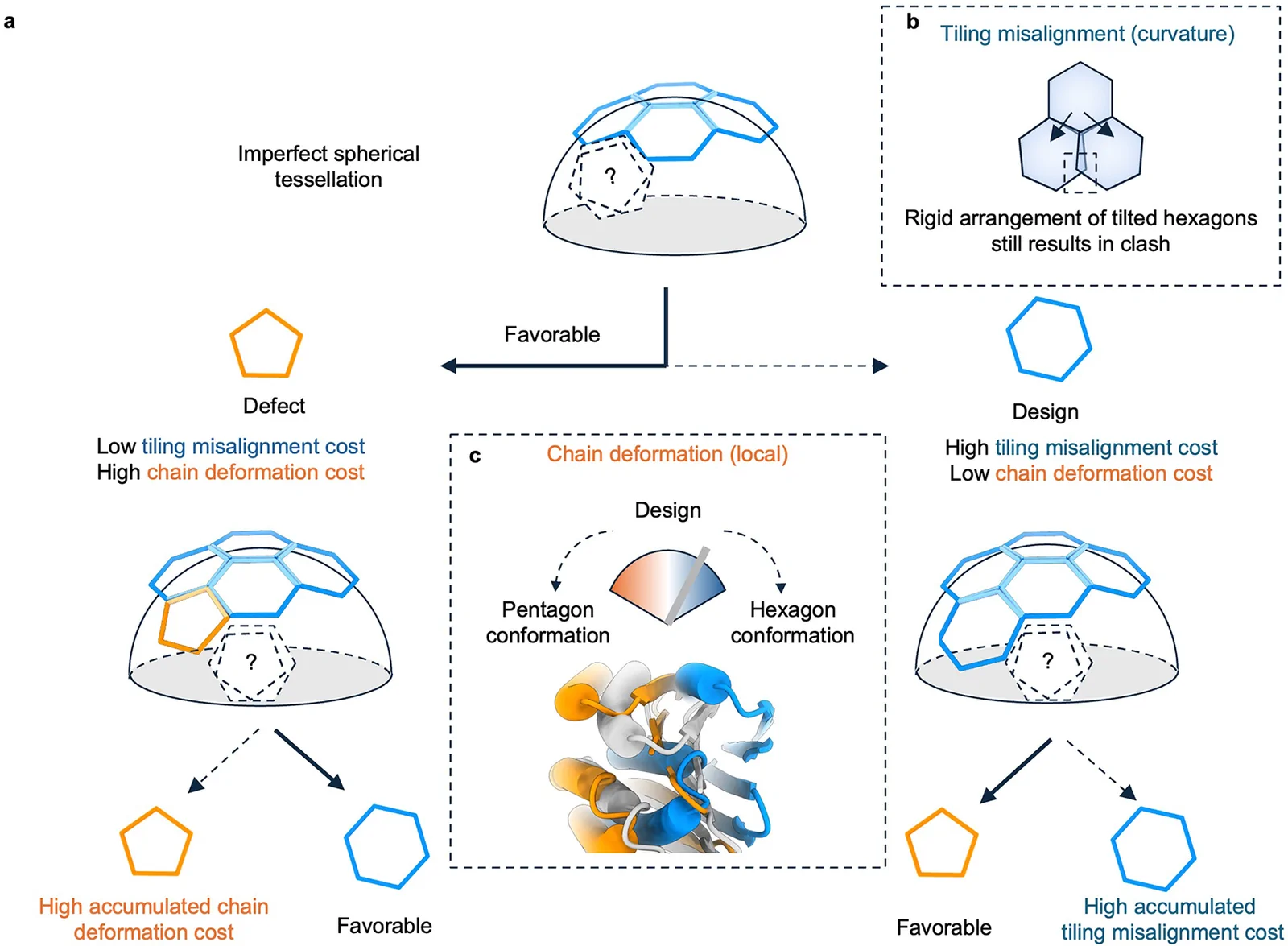
Geometric frustration encountered during cage closure. **a**) Symmetry breaking as a result of two contrasting additions to a self-limiting growing assembly: left, formation of pentagonal defect that accounts for the sphere’s positive curvature, right, formation of designed hexagonal assembly which minimizes local chain deformation. The different types of energy costs are noted below each case: **b**) Tiling misalignment cost is a result of rigid arrangement of tilted hexagons which cannot perfectly tile spherical surfaces, and therefore need to bend to avoid clashes. **c**) Chain deformation cost is the energy required for the designed building block to bend into desired local pentagonal or hexagonal conformation.

We demonstrate the computational design of 2-component quasisymmetric protein assemblies. A key conceptual advance is the departure from traditional explicit modeling strategies, which require multiple explicitly defined conformational states. Instead, our implicit multi-state design approach uses locally flexible structures as inputs that approximate conformational ensembles, leading to particle closure through spontaneous symmetry breaking (Extended Data Fig. 1 and Supplementary discussion). By encoding the curvature of the overall assembly in the local geometry of the subunits, we are able to specify the overall size distribution. In the accompanying manuscript, Lee et al. show that the same principle can be used to generate one-component quasisymmetric cages, demonstrating the generality of the approach. The tunable self-assembly of our quasisymmetric protein materials contrasts with the uncontrolled high-energy carbon vaporization approaches used in the commercial synthesis of fullerenes which make larger fullerenes difficult to access^40^. A fundamental limitation in carbon-based cages is that the C-C bond angle is fixed by atomic hybridization and cannot be reprogrammed, whereas in our protein assemblies the effective inter-subunit bond angle is an explicit design parameter, enabling predictable and continuous tuning of particle size. The two component quasisymmetric cages designed in this work assemble robustly and reproducibly, driven by strong binding affinities between the heterodimeric interfaces grafted on the building blocks. Rapid nucleation and growth contribute to size heterogeneity, which can be qualitatively mitigated by modulating assembly conditions, such as ionic strength, temperature, and crowding agents (Supplementary figure S20–22), which likely improve particle uniformity by slowing down nucleation and growth.

Our ability to predictably control particle size, from 40 to over 200 nm, and to functionalize components through genetic fusion, sets these *de novo* cages apart from naturally occurring viral capsids and other engineered protein particles. The modularity of our assembly approach enables precise control over individual components and straightforward cargo loading for biological applications. Our assemblies provide tools for passively probing the cytosolic biophysical environment, actively recruiting both intra- and extra-cellular receptors, and as protein vehicles for cell-based delivery. The tagging of intracellular proteins with these particles could open new approaches in spatial proteomics^41^ and CryoET-based structure determination^42^. More generally, these genetically encodable cages, along with methodologies for creating new ones, open up new approaches in synthetic biology and metabolic engineering^43^; for example engineered cells could utilize our assemblies as scaffolds to host customizable enzymatic cascades, analogous to bacterial microcompartments^44^. Our methodology, alongside rapid advancements in the *de novo* design of protein building blocks, lays the groundwork for a next generation of programmable biomaterials tailored to intracellular delivery and synthetic biology applications.

## Methods

### Computational design of C2-B linkers

**Extended Data Fig.2 |:**
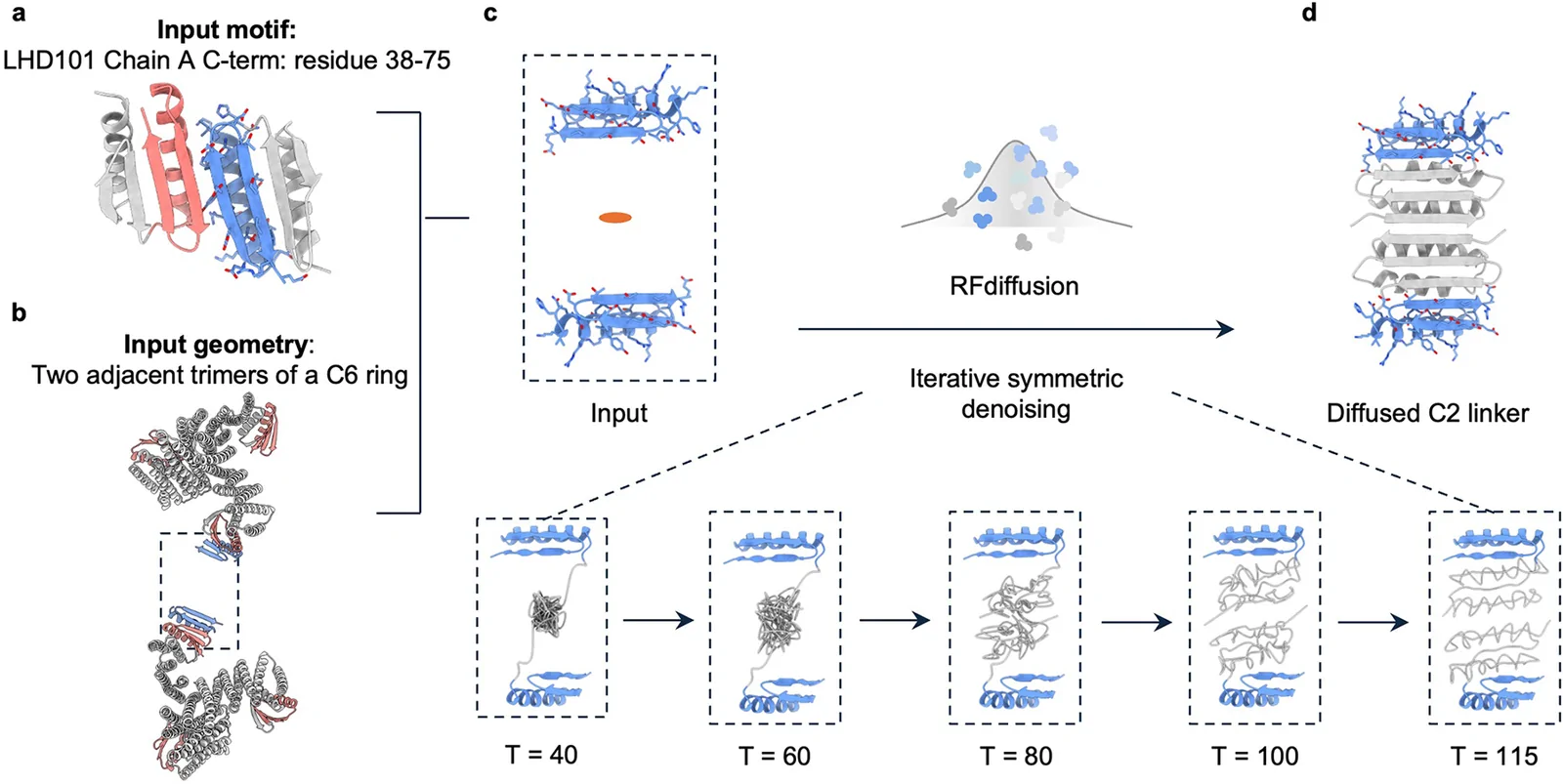
Computational generation of C2 linker Backbones with RFdiffusion. **a**) Cartoon representation of input motif (blue, LHD101 chain A, residue 38–75) extracted from a previously designed heterodimer LHD101. The trimeric building block C3-A is rigidly fused with the complementary binding partner LHD101B (red). **b**) As C3-A trimers are symmetrically arranged in a tilted hexagon at a defined cone angle, two adjacent input motifs (blue) are extracted to create a C2 dimer with missing core. **c**) Symmetric RFdiffusion generatively designs a C2 backbone scaffold to stabilize input motifs in the fixed pre-specified geometry. **d**) Generated C2-B linkers were further sequence designed with ProteinMPNN and filtered by AF2.

The C3-A protein used in this study is a designed homo-trimeric protein^17^ with c terminal rigid fusions of split heterodimer LHD101 A chain^19^ (residue 38–75, PDB ID: 7MWR), which forms a specific non-covalent interface with its binding partner, the LHD101 B chain. Given a target input parameter set (arc length **l** and cone angle **α**), the design calculation of C2-B linker is initialized by symmetrically arranging six copies of C3-A proteins along the z axis with a cyclic C6 symmetry. We then place LHD101 B chains adjacent to C3-A proteins as scaffolding motifs. The rotational degree of freedom ⍵ of C3-A along its 3-fold axis was sampled to control the relative position of C2 binding domain B, which we targeted small gap distance to avoid excessive flexibility. A sample python script for model construction and a test input protein model are available via Zenodo at: https://doi.org/10.5281/zenodo.18892841. RFdiffusion generates C2-symmetric homodimers that maintain the pre-specified heterodimer binding sites (Extended Data Fig. 2). Following sequence design with ProteinMPNN^45^ (--tied_positions option was selected to ensure two chains are identical), the designs were confidently predicted by AlphaFold2^46^ (AF2) to assemble as C2-symmetric oligomers (plDDT > 92, RMSD < 1.2 Å), and to scaffold the heterodimer interface with high accuracy. Finally, to facilitate alternative pentameric ring closure, we docked AF2 predicted structures of C2-B in both 6- and 5-member ring configuration *in silico*, and sub-selected for designs that require less than 4 Å terminal bending to close the 5-member ring.

### Protein expression and purification from *E. coli*

The full list of protein constructs designed and tested in this study is provided in Supplementary Table S5 and the Supplementary DNA Sequences. Synthetic genes for computationally filtered designs were purchased from IDT and cloned into the pET29b+ vector using NdeI and XhoI restriction sites. Constructs were expressed in BL21* (DE3) *E. coli* competent cells with a C-terminal polyhistidine tag. For small-scale expression, transformed cells were cultured in 50 mL Terrific Broth supplemented with 200 mg/L kanamycin and induced for 24 hours at 37°C under the T7 promoter. Cells were harvested by centrifugation, resuspended in Tris-buffered saline, and lysed with five minutes of sonication. The lysates were then subjected to nickel affinity chromatography, washed with ten column volumes of 40 mM imidazole and 500 mM NaCl, and eluted with 500 mM imidazole and 300 mM NaCl. Successful complex formation was confirmed by the presence of both oligomers on SDS–polyacrylamide gel electrophoresis following Ni-NTA pulldown. Proteins of the correct molecular weights were analyzed by electron microscopy. Selected designs were scaled up to 0.5 L for expression and purification under the same conditions.

### *In vitro* cage assembly

For standard *in vitro* assembly, purified cage components were mixed at equimolar ratios to 40 μM per subunit in SEC elution buffer (25 mM Tris-HCl, pH 8.0; 300 mM NaCl) at room temperature. Assemblies also formed across a broad range of conditions, component concentration 5–200 μM and NaCl 150 mM-1.5 M, which modulated particle size and structural uniformity in a construct-dependent manner. For many assemblies, cage uniformity improved when NaCl was increased to 0.6–1.0 M and components were pre-incubated at 50 °C followed by rapid mixing. We also observed enhanced uniformity when components were directly mixed in the IMAC elution buffer (25 mM Tris-HCl, pH 8.0; 500 mM imidazole; 300 mM NaCl), which likely facilitates assembly by stabilizing the subunits and suppressing premature aggregation.

### Negative stain EM

SEC purified sample fractions obtained from *in vitro* assembly were diluted to a final concentration of about 0.5 μM (monomer equivalent) for negative-stain electron microscopy (nsEM) analysis. For each fraction, a 6 μL sample was applied to glow-discharged 400-mesh copper grids coated with formvar and carbon (Ted Pella, Inc.) and left to adsorb for over 2 minutes. Excess sample was blotted, and the grid was stained with 6 μL of 2% uranyl formate. After blotting, an additional 6 μL of uranyl formate was applied for 20 seconds before a final blotting step. Imaging was carried out using a Talos L120C transmission electron microscope operating at 120 kV. Automated nsEM datasets were collected using the E. Pluribus Unum software (EPU, FEI Thermo Scientific), and further processed using CryoSPARC software^47^. Micrographs were uploaded to the CryoSPARC platform, where contrast transfer function (CTF) corrections were performed. Two-dimensional class averages and three-dimensional maps were generated using CryoSPARC. Particles from selected classes were used to generate an *ab initio* model, which was subsequently refined using C1 symmetry and further adjusted for I symmetry as needed.

### Identification of off-targets assemblies from nsEM 2D class averages

In addition to the major assembly product of T = 3 cage (structural analog of C60), we also observed a distribution of minor species under nsEM micrographs 2D class averages (Extended Data Fig. 4a), including particles of different sizes and anisotropic shapes. Based on careful examination of 2D class averages, we isolated a subset of particles that correspond to T = 4 icosahedron (C80) by 3D nsEM map. However, we couldn’t reconstruct and identify other particles due to polydispersity, and we speculate these assemblies correspond to a variety of fullerene cage analogs, including C70 (Extended Data Fig. 4b–c. The formation of these non-uniform off-target assemblies could be under kinetic control, which we aim to optimize in future studies. For both T = 3 and T = 4 cages, all pentagonal rings are located at the vertices of an icosahedron, however they no longer occupy vertices when lower symmetry particles are formed.

### X-ray crystallography

Crystals of C2-B-α20 linker were grown using protein purified as described above. A concentrated sample was used for crystallization. The crystallization screening was performed using a Mosquito LCP by STP Labtech. Crystals grew in 2.0 M Ammonium sulfate, 0.1 M Sodium acetate pH 4.6. Crystals were harvested directly from a screening tray, and flash cooled in liquid nitrogen. 20% glycerol was used as a cryoprotectant. X-ray diffraction was performed at NSLS2 beamline 17-ID-2. Crystal diffracted to 2 Å. Data was processed with XDS^48^, and merged/scaled using Pointless/Aimless in the CCP4 program suite^49^. The structure was phased by molecular replacement using the designed structure as the search model by Phaser^50^ and refined with Phenix^51^. Following molecular replacement, the models were improved, and efforts were made to reduce model bias. The final structure was refined in Phenix. Model building was performed using COOT^52^. The final model was evaluated using MolProbity^53^. Data collection and refinement statistics are recorded in Extended Data Table 1. Data deposition, atomic coordinates, and structure factors reported in this paper have been deposited in the Protein Data Bank (PDB), http://www.rcsb.org/ with accession code 9NDL.

### CryoEM and CryoET Data Collection and Processing

Three microliters of purified cages at approximately 40 μM in 25 mM Tris-HCl and 300 mM NaCl (pH 8) were applied to glow-discharged 300 mesh R2/2 holey carbon C-flat grids. The grids were blotted using a Vitrobot Mark IV (Thermo Fisher Scientific) for 4 seconds at room temperature and ~100% chamber humidity. For data collection, the grids were imaged on a Glacios cryo-electron microscope (Thermo Fisher Scientific) operated at 200kV using SerialEM data collection software^54^ and images were recorded on a K3 Summit electron detector at a magnification of 22,000x at 2x binning corresponding to a calibrated sampling of 1.82 Å per pixel. Tilt series were acquired using a dose-symmetric collection scheme^55^ at 3 degrees increments with low dose settings and a defocus range of −2 to −5 μm. Each tilt series was composed of 41 individual tilt images, with a total accumulated dose of 110 e^−^/Å^2^. Tomogram analysis steps were performed using EMAN2 version 2.3 Tomo pipeline. Tilt image movie stacks were motion-corrected using MotionCor2^56^, aligned and reconstructed into 3D tomogram volumes using *e2_tomogram.py.* CTF estimation for each tilt image was performed using the EMAN program *e2spt_tomoctf.py.* Data deposition, atomic coordinates, and structure factors reported in this paper have been deposited in the Protein Data Bank (PDB), http://www.rcsb.org/ with accession code 9OP9.

For single particle cryoEM analysis (SPA), the dataset for C2-B-α20 sample (T = 3 cage) was collected automatically using SerialEM^46^ and used to control a ThermoFisher Titan Krios 300 kV, microscopes was equipped with a K3 Summit direct electron detector^57^ and BioQuantum Gif energy filter and operated in counting mode. Random defocus ranges spanned between −0.8 and −1.8 μm using image shift. 7,920 movies with a pixel size of 0.843, total dose of 48.3 e^−^/Å^2^ were recorded with a total exposure of 5 seconds over 99 frames. All data processing was carried out in CryoSPARC^47^. Default parameters were used for refinement unless otherwise noted. The video frames were aligned using Patch Motion, and defocus and astigmatism values were estimated using the Patch Contrast Transfer Function. Curate Exposures was used to remove 1,295 micrographs, leaving 6,625 good micrographs (Supplementary figure S8a). Micrographs were then denoised in using the Micrograph Denoiser, with the pretrained model. 210 particles were picked on 21 micrographs to generate templates. Using these templates, Template Picker was used with particle diameter set to 500 Å, Maximum number of local maxima to consider set to 100, Pick on denoised micrographs set to true. 278,552 particles were picked, following an Inspect Picks job 258,422 particles were remaining, in total 142,674 particles were extracted with a box size of 1400 pixels, Fourier cropped to 350 pixels. 2D classification was run with 300 classes, number of online-EM iterations set to 40, number of final full iterations set to 10. 4 classes were selected with 49,467 particles and another round of 2D classification was run with: 50 classes, number of online-EM iterations set to 40, number of final full iterations set to 10. 8 classes with 49,224 particles were selected (Supplementary figure S8b). An *ab initio* was run with 5 volumes using Icosahedral symmetry (Supplementary figure S8c). 22,924 particles generated an Ab Initio volume that closely matched the expected design structure and tomography reconstruction. This volume and particles were used for a Non Uniform Refinement (Supplementary figure S8d–f). All volumes were shown using ChimeraX^58^. Data deposition, atomic coordinates, and structure factors reported in this paper have been deposited in the Protein Data Bank (PDB), http://www.rcsb.org/ with accession code 9OM3.

### Construction of cage models based on EM density map

Initial hexamer and pentamer models were created by taking the designed model as the hexamer model, and creating a C5 pentamer model using Rosetta's *make_symmdef_file*^22^ to build a C5 complex that preserved as much as possible of the inter-subunit geometry from the original hexameric design. These C5 and C6 rings were then docked into the cryoEM density using Chimera. When C5 and C6 subunits overlapped, the C6 subunits were used as a reference. This gave us a rough initial model of the cage.

Then the full cage structure was refined using Rosetta’s cryoEM refinement protocol^23^. One asymmetric unit was extracted from the initial rough model. We then used Rosetta’s *relax* with icosahedral symmetry restraints to refine the entire complex. Additionally, during this refinement weak atom-pair constraints were used for all subunit interfaces within the asymmetric unit to prevent the various interfaces from diverging too much. All interface Cα’s within 9 Å were constrained to maintain their distances from the original hexamer design. A single repeat of relax yielded the final model shown in (Fig. 3c).

### Cage cellular uptake experiments

For confocal imaging: HEP3B cells (ATCC HB-8064, authenticated and tested for mycoplasma contamination by provider) were plated at 35,000 cells/well in an 8-well Labtek plate a day prior to the treatment. 250 nM of GFP-labeled IGF2R, ASGPR, and Sortilin cage were added to HEP3B cells for a 24 hour treatment. Cells were then fixed with 4% paraformaldehyde and stained with LAMP1 for lysosomal staining and DAPI for nuclear staining. Cells were washed with DPBS and imaged with a Nikon A1R confocal microscope using a Plan Fluor ×60, 1.30-NA oil objective.

For flow cytometry: HEP3B cells were plated at 7,500 cells/well in a 96-well plate a day prior to the treatment. 250 nM of GFP-labeled cages were added for a 3 hour, 6 hour, and 24 hour treatment. Cells were then trypsinized, quenched, and transferred to a 96-well-U-bottom plate. Cells were washed three times with DPBS and were analyzed on Attune Flow Cytometer (Thermo Fischer).

### *In cellulo* expression of cages

Mammalian cell lines: Human bone osteosarcoma epithelial cells (U2OS cells) were cultured in DMEM (Gibco, Cat. No. 11995073) containing high glucose and sodium pyruvate supplemented with 10% fetal bovine serum (FBS, Gemini bio-products, Cat. no. 100–106) and 50 U/mL penicillin, and 50 μg/mL streptomycin (Gibco^™^, Cat. No. 15140122), and maintained at 37°C in a humidified incubator with 5% CO_2_. Cells were regularly split in fresh medium upon reaching 80–90% confluency. All cells were routinely tested for mycoplasma by PCR screening of the conditioned medium.

Transient transfection of mammalian cell lines: To express particles in mammalian cells, we introduced plasmids encoding the particle-forming two-component monomer proteins into cells. Gene fragments were synthesized by GenScript and assembled into plasmid backbones via Gibson assembly. All constructs were sequence-verified by nanopore sequencing services (Plasmidsaurus). Plasmids were then delivered by either transient transfection or lentiviral transduction, enabling intracellular protein expression and self-assembly into particles inside cells.

For transfection, U2OS cells were seeded as 60–70% confluency in a 6-well glass bottom plate (Cellvis, Cat. No. P06–1.5H-N) on the day before transfection and were transfected with 1 μg of plasmid DNA per well using FuGENE HD^™^ (Promega, Cat. no. E2312) reagent per manufacturer guidelines. 24 hour post-transfection, fresh DMEM medium was replaced. And imaging experiments were usually carried out between 48 to 72-hour post-transfection.

### Lentivirus production and cell transduction for stable cell lines

Human embryonic kidney cells (HEK293T cells) (6×10^6^ per 10 cm dish) were plated in antibiotic free DMEM supplemented with 10% FaBS. The next day, cells were transfected with transgene plasmid (pLH2601 or pLH2704) together with lentivirus packaging plasmids pMD2.G (a gift from Didier Trono (Addgene plasmid # 12259)) and psPAX2 (a gift from Didier Trono (Addgene plasmid # 12260)), using FuGENE HD^™^ transfection reagent following manufacturer’s protocol. 24 hours later, 6 mL antibiotic free DMEM was replaced and the supernatants were collected at both 48 and 72 hour post-transfection, and stored at 4°C. 1.5 mL fresh virus titers were added to 35 mm dish culture of U2OS cells. The rest of the virus titers were concentrated by centrifugation at 4,000 rcf for 30 minutes in an Amicon Ultra-15 30 KDa centrifugal filter (MilliporeSigma, Cat. No. UFC903024). Concentrated viral suspensions were aliquoted and stored at −80°C until later use. After cell lines stabilized, they were expanded in culture to 10 cm dishes, and enriched for high GFP expression cell populations by fluorescence-activated cell sorting. The cell lines were frozen in 10% DMSO (Sigma-Aldrich, Cat. no. D2650– 100) in DMEM medium, and thawed for use in experiments whenever needed.

### Passive rheological probes imaging and single particle tracking

To image 40nm-GEMs and *de novo* cages in U2OS cells, Andor Yokogawa CSU-X confocal spinning disc on a Nikon Ti2 X1 microscope was used at 488 nm excitation with 100% laser power. Fluorescence was recorded with a sCMOS Prime 95B camera (Photometrics) with a 60x objective (pixel size: 0.18 μm), at a 100 fps image capture rate for a total of 2 seconds. The tracking of particles was performed with the Mosaic suite of FIJI, using the following parameters: radius = 3, cutoff = 0, Per/Abs = 0.5, a link range of 1, and a maximum displacement of 5 px, assuming Brownian dynamics. All trajectories were then analyzed with the GEMspa (GEM single particle analysis) software package that we are developing in house^49^. Mean-square displacement (MSD) was calculated for every 2D trajectory, and trajectories continuously followed for more than 10 time points were used to fit with linear time dependence based on the first 10 time intervals to quantify time-averaged MSD: MSD(*T*) = 4*D*_eff_*T*, where *T* is the imaging time interval and *D*_eff_ is the effective diffusivity with the unit of μm^2^/s. To determine the ensemble-time-averaged MSD, all trajectories were fitted with MSD(*τ*)_T−ens_=4*Dτ*^*α*^ where *α* is the anomalous exponent, with *α* = 1 being Brownian motion, *α* < 1 suggests sub-diffusive motion and *α* > 1 as super-diffusive motion. All analyzed trajectories within one cell were averaged to present single cell *D*_eff_ for each condition (Supplementary figure S16).

### Measurement of particle intensity and colocalization

To fix U2OS cells, 4% paraformaldehyde in PBS was added to each of the imaging wells for 10 min at room temperature. The cells were subsequently washed three times in 1x PBS before imaging. Andor Yokogawa CSU-X confocal spinning disc on a Nikon Ti2 X1 microscope was used to capture particles within the cell at one focal plane. The particles were identified with the LoG detector algorithm with manual thresholding and quality examination in trackmate plugin Fiji^59^, the mean intensity and the x,y location of each particle were measured. The mean intensity of the cytosolic regions without particles was also measured across cells to determine the background intensity. Thereafter, the background subtracted GFP mean intensity was calculated for each particle. The median value of all the quantified particles was denoted for each type of particle in individual experiment, and the fold change of particle intensity was calculated by normalizing the median value of *de novo* cages to 40nm-GEMs.

A custom Python script is developed to quantify the colocalization between GFP particles and mScarlet particles. Due to potentially immobile bright particles being detected in mScarlet channel but not in GFP channel, we set up criteria to pair exclusive green and red particles before measuring the distance between them. Initially, all green particles were assigned to the nearest neighbor red particles, if more than one green particle were paired with the same red particle, they were not considered as exclusive pairing thereby removed from further analysis. The identified pairs of green and red particles were considered colocalization only if their locations were within 180 nm (1 pixel) difference. As a control analysis, randomized green particle locations were generated and assigned within the cytosolic region of the cell, and the same function was used to identify pairing green and red particles, and then measure distance between them. We reported the percentage of colocalized green particle number over the total exclusive paring green-red particle number in individual cells.

## Extended Data

**Extended Data Fig.3 |:**
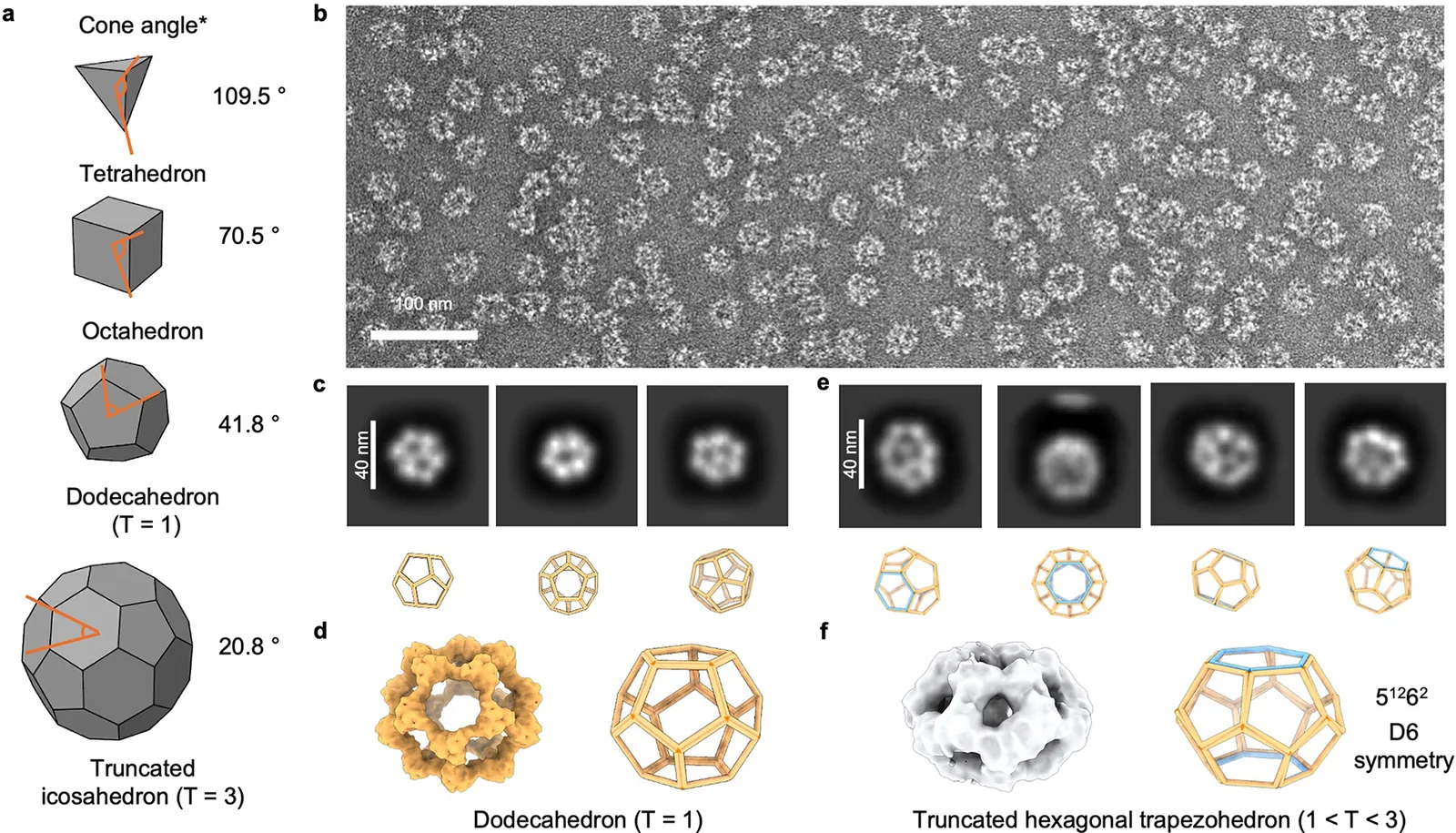
Vertex angle–guided polyhedral cage formation. **a**) Schematic of different polyhedral cages and their corresponding intersecting angles between adjacent vertices. *In vitro* mixing of C3-A with the C2-B-α30 linker yields assemblies comprising a mixture of T = 1 and T = 1–3 cages. **b**) Representative nsEM micrograph of the assembled cages, the reported morphology was observed across three independent experiments. **c**) nsEM 2D class averages revealing assemblies consistent with dodecahedral geometry. **d**) Comparison between the experimentally reconstructed nsEM 3D density map (left, with imposed Icosahedral symmetry) and a regular dodecahedron (right). **e**) Selected 2D class averages showing additional cage populations, potentially corresponding to a larger cage containing 12 pentagonal faces and 2 hexagonal faces. **f**) nsEM 3D reconstruction map without imposed symmetry (left) compared with a cage model with D6 symmetry (right).

**Extended Data Fig.4 |:**
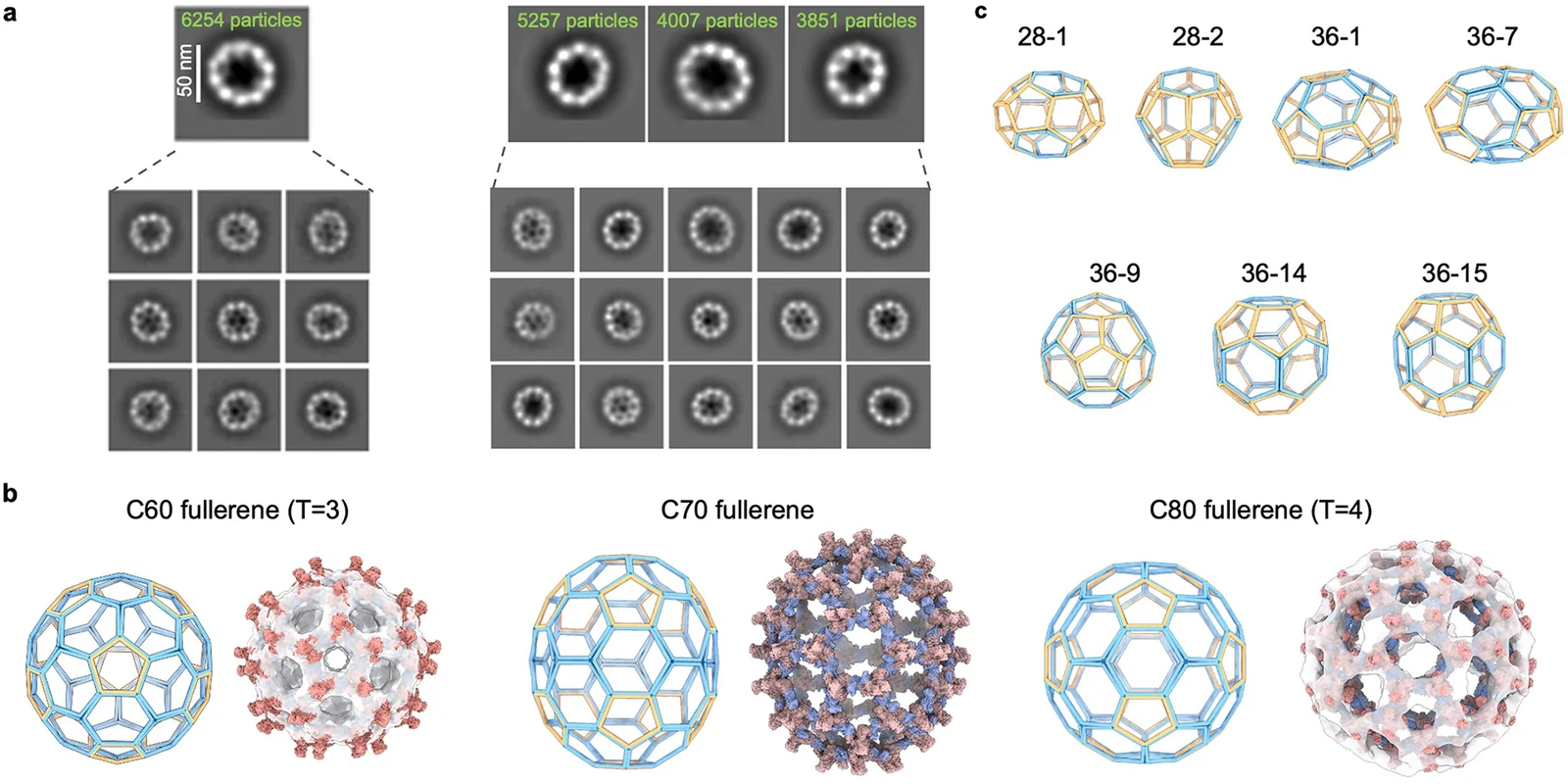
nsEM characterization of the T ~ 3 cage. **a**) Selected subsets of nsEM 2D class averages showing distinct cage architectures observed from for the T ~ 3 cage sample. Scale bar = 50 nm. **b**) A model of C60 fullerene cage (T=3) and an experimental nsEM 3D reconstruction map (with applied Icosahedral symmetry, left). An elongated cage with high aspect ratio could potentially be C70 fullerenes (middle). C80 fullerene topology (T = 4) and experimental nsEM 3D reconstruction (with icosahedral symmetry applied, right) agrees with the design model. **c**) Other possible cage topologies from hexagon-pentagon mixed tiling that have been reported.**</Figure_Caption>**

**Extended Data Fig.5 |:**
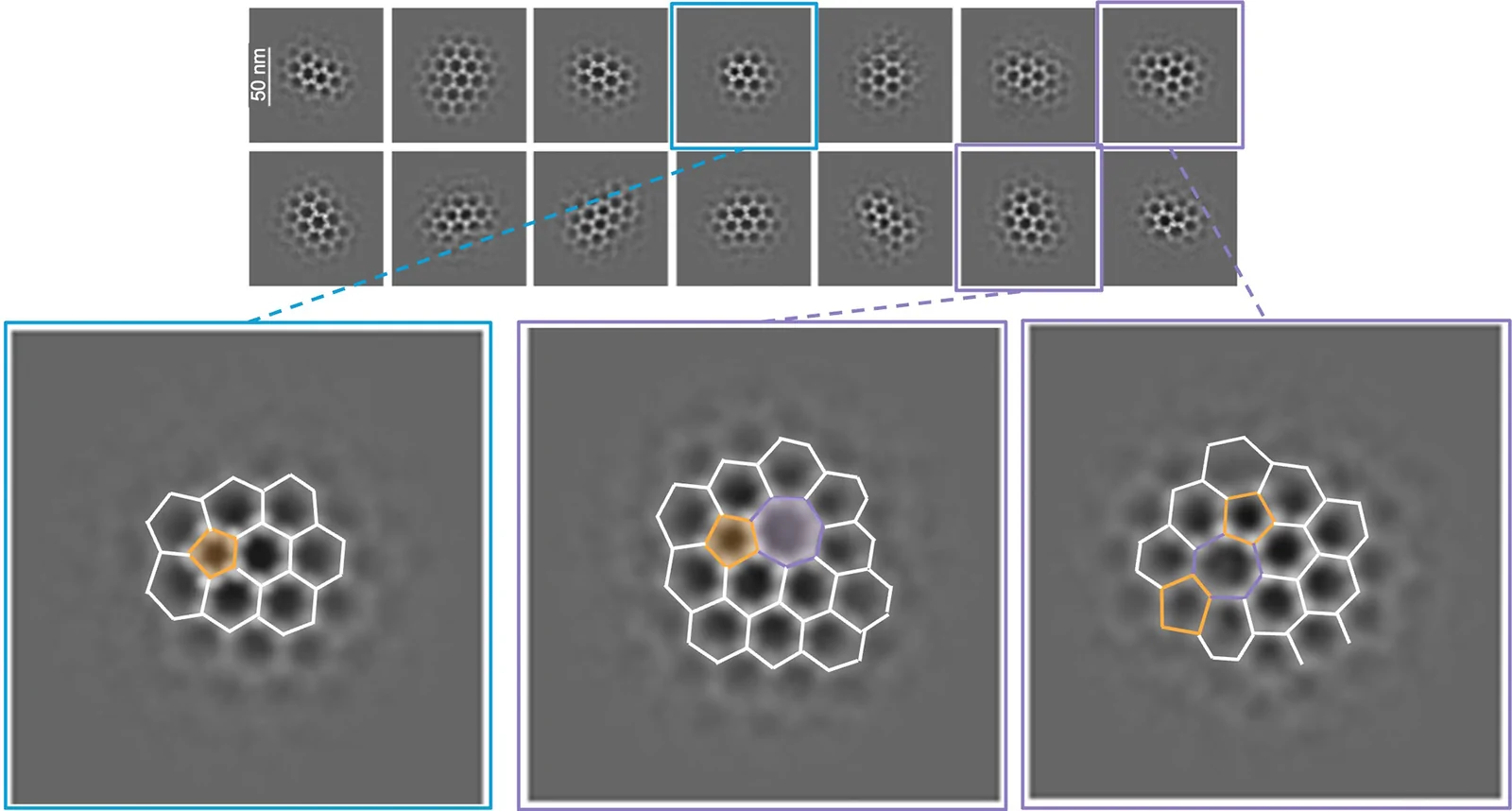
Local defects analyses based on cryoEM 2D class averages. For cages with T < 9, only pentagonal defects were observed. For high T cages, both pentagonal (orange) and heptagonal defects (purple) were observed. Although heptagonal defects generate negative Gaussian curvatures that are energetically unfavorable, they could be formed during cage closure under kinetic control.

**Extended Data Fig.6 |:**
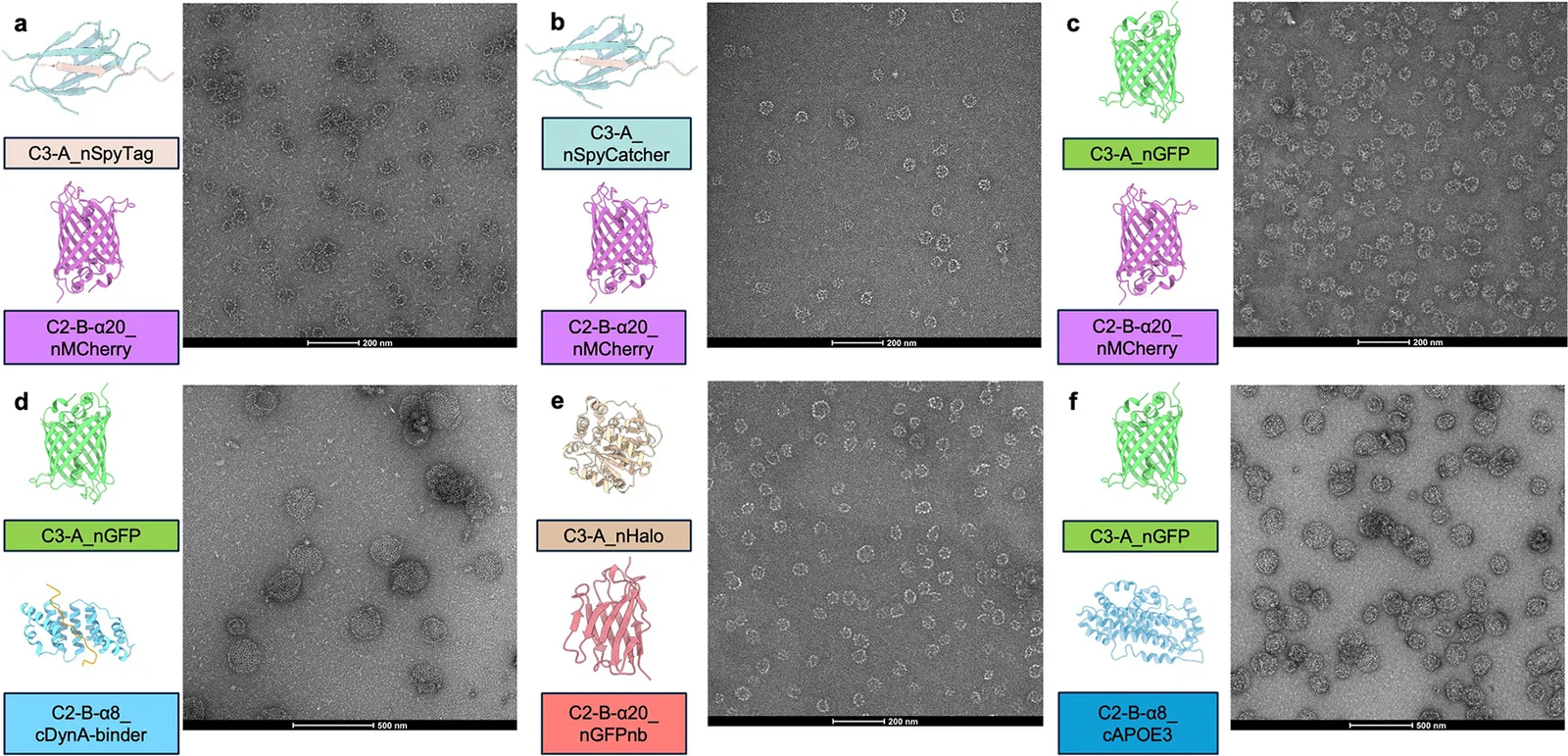
NsEM characterization of functionalized protein cage assemblies. Representative nsEM images show the formation of cage structures utilizing various building block combinations terminally fused to diverse proteins of interest. Similar particles were observed across at least three independent experiments.

**Extended Data Table 1:** X-ray diffraction data collection and refinement statistics for C2-B-α20 linker.

	C2-B-α20 (N9DL)

Resolution range	28.99 – 2.15 (2.21 – 2.15)
Space group	C 2
Unit cell	61.19, 93.30, 60.69; 90, 119.94, 90
Unique reflections	15664 (1104)
Multiplicity	4.3 (4.3)
Completeness (%)	98.00 (94.5)
Mean I/sigma (I)	8.70 (2.70)
Wilson B-factor	36
R-merge	0.094 (0.524)
CC_1/2_	0.993 (0.863)
Reflections used in refinement	15518 (1388)
R-work	0.2029 (0.2583)
R-free	0.2523 (0.3319)
Number of non-hydrogen atoms	2463
macromolecules	2427
solvent	36
Protein residues	318
RMS (bonds)	0.003
RMS (angles)	0.530
Average B-factor	
macromolecules	55
solvent	52

Statistics for the highest-resolution shell are shown in parentheses.

**Extended Data Table 2:** Cryo-EM data collection, refinement and validation statistics.

	#1 Two-component protein cage (T=3) (EMDB-70605) (PDB 9OM3)	#2 Two-component protein cage (T=3) (EMDB-70685) (PDB 9OP9)

Data collection and processing		
Magnification	105,000 ×	22,000 ×
Voltage (kV)	300	200
Electron exposure (e–/Å^2^)	9.66	120
Defocus range (μm)	0.8–1.8	2–4
Pixel size (Å)	0.843	0.885
Symmetry imposed	Icosahedral	Icosahedral
Initial particle images (no.)	107,197	599
Final particle images (no.)	11,155	462
Map resolution (Å)	17.45	31.6
FSC threshold	0.143	0.143
Map resolution range (Å)	20.02 – 15.91	N/A
Refinement		
Initial model used (PDB code)	Design model	Design model
Model resolution (Å)	17.45	31.6
FSC threshold	0.143	0.143
Model resolution range (Å)	20.02 – 15.91	N/A
Map sharpening *B* factor (Å^2^)	0	N/A
Model composition		
Non-hydrogen	553,560	553,560
atoms	71,640	71,640
Protein residues Ligands	NA	NA
*B* factors (Å^2^)		
Protein	98.11	98.11
Ligand	NA	NA
R.m.s. deviations		
Bond lengths (Å)	0.017	0.019
Bond angles (°)	1.435	1.565
Validation		
MolProbity score	0.86	0.93
Clashscore	1.35	1.72
Poor rotamers (%)	0	0
Ramachandran plot		
Favored (%)	100	100
Allowed (%)	0	0
Disallowed (%)	0	0

## Supplementary Material

SI

Video-1_single-color_C3-A-nGFP_C2-B-α10_Cage

## Figures and Tables

**Fig. 1 |:**
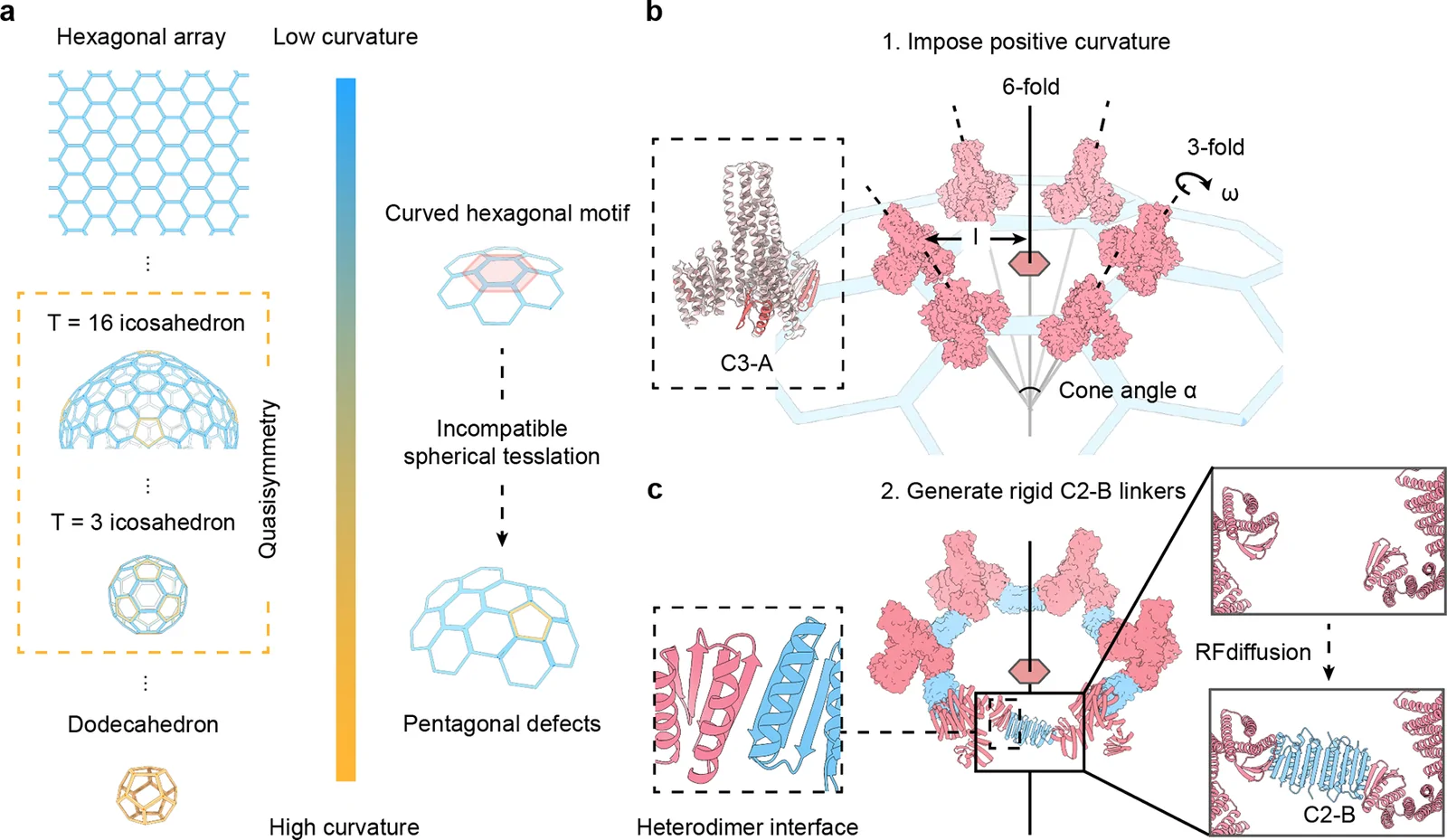
Computational design strategy for generating quasisymmetric particles. **a**) Regular hexagons tessellate flat 2D lattices with zero curvature. In contrast, twelve regular pentagons form a dodecahedron with positive curvature, creating an angular curvature of 36 degrees at each vertex. Quasisymmetric icosahedral cages do not maintain constant curvature; instead 12 pentagons are combined with varying numbers of hexagons to form closed particles with different triangulation numbers. The ratio of pentagons to hexagons determines overall curvature. **b**) We approximated quasisymmetric particles using positively curved, symmetric hexagonal building motifs. Six copies of the homotrimer (inset) were symmetrically arranged into a hexagonal pattern and bent inward to impose positive curvature. **c**) Next, we used RFdiffusion, a generative protein design method, to create dimeric linkers that rigidify the designed curvature. A previously validated heterodimeric interface (inset) drives binding between the trimer and the designed dimeric linkers, ensuring proper particle self-assembly.

## Data Availability

The principal data supporting the findings of this work are available within the figures and the Supplementary Information. Design models for the cage building blocks are available via Zenodo at: https://doi.org/10.5281/zenodo.18892841. The heterodimer protein pair LHD101 was derived from study https://www.science.org/doi/10.1126/science.abj7662 (PDB ID 7MWR). Coordinates and structure factors are available in the Protein Data Bank with the following accession codes: 9NDL (C2-B-α20), 9OM3 (T=3 cage, cryoEM structure), and 9OP9 (T=3 cage, cryoET structure).

## Code availability

RFDiffusion code can be downloaded from https://github.com/RosettaCommons/RFdiffusion. ProteinMPNN is available from https://github.com/sokrypton/ColabDesign/. AlphaFold2 is available from https://github.com/google-deepmind/alphafold. Scripts for generating curved hexagon are available via Zenodo at: https://doi.org/10.5281/zenodo.18892841.
