# Supplementary material for: De novo design of quasisymmetric two-component protein cages
Source: Nature. Author manuscript; Available in PMC 2026 Jul 29. (PMC13412984; doi:10.1038/s41586-026-10464-0)
Supplement: Supplementary file 1 [file NIHMS2194045-supplement-SI.pdf]

Supplementary Materials for

De novo design of quasisymmetric two-component protein cages

Shunzhi Wang[1,2,3*], Ying Xie[3], David Chemielewski[1,2], Connor Weidle[1,2], Shu Tong[3], Green Ahn[1,2], Ryan D. Kibler[1,2], Cindy Hernandez[3], Wei Chen[1,2], David Camilo Duran[3], Ann Carr[1,2], Asim K. Bera[1,2], Sangmin Lee[1,2,4], Justin Decarreau[1,2], Alex Kang[1,2], Evans Brackenbrough[1,2], Emily Joyce[1,2], Kejia Wu[1,2], Andrew J. Borst[1,2], Andrew Favor[2,5], Buwei Huang[2,5], Frank DiMaio[1,2], Liam Holt[3], David Baker[1,2,5,6*]

1. Department of Biochemistry, University of Washington, Seattle, WA, USA.
2. Institute for Protein Design, University of Washington, Seattle, WA, USA.
3. Institute for Systems Genetics, New York University Langone Medical Center, New York, NY, USA.
4. Department of Chemical Engineering, Pohang University of Science and Technology (POSTECH), Pohang, Republic of Korea.
5. Molecular Engineering and Sciences Institute, University of Washington, Seattle, WA, USA.
6. Howard Hughes Medical Institute, University of Washington, Seattle, WA, USA.

*To whom correspondence should be addressed:
shunzhi.wang@nyulangone.org
dabaker@uw.edu

Table of Contents

Supplementary Discussion

Energetic Landscape and Conformational Mechanism of Assembly ……………………………….3
Implicit Multi-state vs. Explicit Multi-state and Negative Design ……………………………………3
Heptagonal defects in high-T shells …………………………………………………………………4

Supplementary Figures

Fig. S1 Characterization of cage assemblies with SEC and DLS …………………………….…...5
Fig. S2 Quantification of *in vitro* cage assembly yields ……………………..……………….….…. 6
Fig. S3 Electron microscopy characterization of T~16 cages ……………………………….…….7
Fig. S4 Electron microscopy characterization of T~25 cages …………………………………….8
Fig. S5 Characterization of C3-A_nGFP_C2-B-α8 cage variants ………………………………9
Fig. S6 Impact of C-2B linker geometry on cage size …………………………………...…. 10
Fig. S7 Aggregated assemblies of low-cone-angle linkers ……………………….………...10
Fig. S8 Single-particle CryoEM of T ~ 3 cage ………………………………………………….11
Fig. S9 Symmetry breaking of C3-A during cage assembly ……………………………………12
Fig. S10 Defect distribution within high-T cages ……………………………………………….12
Fig. S11 CryoEM imaging of protein cages ………………………………………………………13
Fig. S12 SDS-PAGE analysis of representative functionalized building blocks………………..14
Fig. S13 SEC characterization of functionalized cages and their components ………………..15
Fig. S14 SEC validation of Cas9-GFP encapsulation ……………………………………………16
Fig. S15 Cellular uptake kinetics of EndoTag-functionalized cages ……………………………16
Fig. S16 Characterization of passive rheological cage probes in the cytosol …………………17
Fig. S17 Comparison of intracellular cages and condensate formation ………………………18
Fig. S18 Passive rheological probes in the *S. cerevisiae* cytosol ………………………………19
Fig. S19 Mutations at the assembly interface impair cage assembly ……………………………20
Fig. S20 Subunit stoichiometry influences *in vitro* cage assembly ……………………………..21
Fig. S21 Subunit concentration influences *in vitro* cage assembly ………………………………21
Fig. S22 Glycerol concentration modulates *in vitro* cage assembly ……………………………..22

Supplementary Tables

Table S1 Summary of T numbers, predicted cage sizes, and molecular weights ………………23
Table S2 Purification yields of protein cage building blocks ………………………………………24
Table S3 In vitro cage assembly yields of representative constructs …………………….….25
Table S4 Plasmids and cell lines ……………………………………………………………….….26
Table S5 Sequences of designed protein constructs …………………………………….….….27

References ………………………………………………………………………………………...…33

Other supplementary Materials for this manuscript include the following:

Supplementary Video S1**.** Real time video of single color C3-A-nGFP_C2-B-α10 cages assembled in U2OS cells. The video was captured every 50 ms for a total of 5 seconds.

Supplementary DNA sequences

Supplementary Discussion

Energetic Landscape and Conformational Mechanism of Assembly. We propose that the observed distinct conformations at the junctions represent points within a continuum of structural states rather than discrete, pre-specified switches. In the absence of binding partners, both homo-oligomeric C3-A and C2-B sample a range of conformations in solution, consistent with the observed flexibility of their arm/linker regions. Upon assembly, this intrinsic flexibility allows the components to locally constrain into the two required junction environments: hexagon-hexagon (HH) and hexagon-pentagon (HP).

This interpretation is consistent with several of our observations:

- Quasi-equivalence: The three chains within a single C3-A trimer adopt subtly different poses in the cryoEM model, showing intrinsic local variability.
- Observed heterogeneity: Our nsEM 2D class averages reveal heterogeneity within the assembled structures (e.g., mild faceting/shape distortions and occasional local linker defects), indicating that the local environment is richer than an ideal HH/HP dichotomy.

Regarding the energetic landscape, assembly is driven by the strong, pre-specified heterodimeric A–B interaction (Kd ≈ 10 nM; $\Delta G^\circ \approx -10.9$ kcal·mol^{-1} at 298 K). Curvature is achieved because the substantial free energy decreases from forming new A-B interfaces consistently outweighs the local elastic deformation penalties. This process adheres to an "elastic funnel" mechanism, where curvature is distributed across the subunits via many small, cooperative adjustments (local induced fit), rather than being enforced by a long-range, two-state conformational switch (allostery). We have not observed evidence for a global allosteric switching event (e.g., an assembly-competent to incompetent interconversion). Functionally, the strong A-B interaction ensures robust connectivity and assembly fidelity, while the soft modes and compliance within the arm/linker regions provide the necessary adaptability to tile both HH and HP junction types and accommodate the required curvature. The observed symmetry breaking (pentagon placement) follows from topological necessity plus this local compliance, not a discrete conformational switch.

Implicit Multi-state vs. Explicit Multi-state and Negative Design. Our design utilizes an implicit multi-state strategy: a single, robust heterointerface combined with highly compliant linkers creates an elastic energy landscape broad enough to stabilize both hexagon-hexagon (HH) and hexagon-pentagon (HP) junctions through minor induced-fit adjustments. We intentionally leverage the geometric incompatibility inherent between an ideal flat hexagonal tessellation and spherical curvature, allowing the system to distribute frustration and achieve global curvature through soft modes across the lattice spontaneously. This inherent frustration not only tolerates but necessitates multiple local states, naturally yielding two conformational basins consistent with the principles of quasi-equivalence and fullerene-like tiling. In contrast, explicit multi-state paradigms typically require designers to either (i) enumerate state-specific geometries (e.g., unique edge angles that enforce a chosen T-number in DNA shells[1]), (ii) enumerate state-specific parts (e.g., distinct BMC pentamer/hexamer/trimer proteins for non-equivalent lattice positions[2]), or (iii) encode two discrete subunit conformers and co-design interfaces so that X-state and Y-state oligomers switch cooperatively[3,4]. Relatedly, two-state hinges are designed to populate apo

and effector-bound conformations with quantitative pre-equilibrium control. Our approach instead positively encodes compatibility with multiple intended local states without explicitly enumerating them, while still applying mild implicit negative design to suppress completely off-pathway self-associations. The result is a robust yet tolerant assembly mechanism in which global curvature emerges from many low-energy deformations (an elastic funnel) rather than discrete, pre-specified state-specific fits.

Heptagonal defects in high-T shells. Heptagons are negative-curvature disclinations that can arise as stress-relief features in large quasi-icosahedral shells. Prior theory and experiments on crystalline order on curved surfaces show that spherical systems lower their elastic energy by introducing 5-7 defect pairs (“scars”) once they are sufficiently large[5,6], in contrast to defect-free structures in flat space[7]. This phenomenon, first described for colloidosomes and soft-matter systems, has recently been directly visualized at molecular resolution in the influenza C virus HEF lattice[8]. Using cryo-ET, these authors showed that HEF trimers form an extended hexagonal lattice decorated with spatially organized 5- and 7-fold defects and grain boundaries whose number increases linearly with system size, precisely matching continuum elastic predictions. Our observation of heptagonal defects in high-T protein cages are consistent with this general behavior.

In our C3-C2 architecture, strong A-B interaction connects the contact network while small hinge/shear adjustments distribute curvature. At higher triangulation numbers, where residual elastic mismatch accumulates during the late stages of growth, occasional heptagons are therefore expected as a mechanism to dissipate accumulated stress and minimize the overall elastic energy[9]. In biological lattices, these defects reflect an adaptation to the unavoidable geometric constraints of closing a spherical shell rather than a breakdown of design.

Consistent with models of diffusion-limited growth on curved membranes[10], defects are likely introduced late in assembly when perfect pentamer/hexamer tiling becomes geometrically difficult to maintain. High-T shells may also form via merger of meso-scale intermediates or pre-assembled fragments, in which case local mis-registry can lock in 7-fold motifs that cannot subsequently anneal.

Consistent with this framework, the heptagons we observe (Ext. Data Fig. 5) are rare and spatially localized. We expect minor negative effects on global mechanical stability, with structural impact confined to subtle, local perturbations in geometry or stiffness near the defect core. This interpretation aligns with elastic-shell analyses linking defect patterns and shell shape to the balance between stretching and bending energies[11].

Supplementary Figures

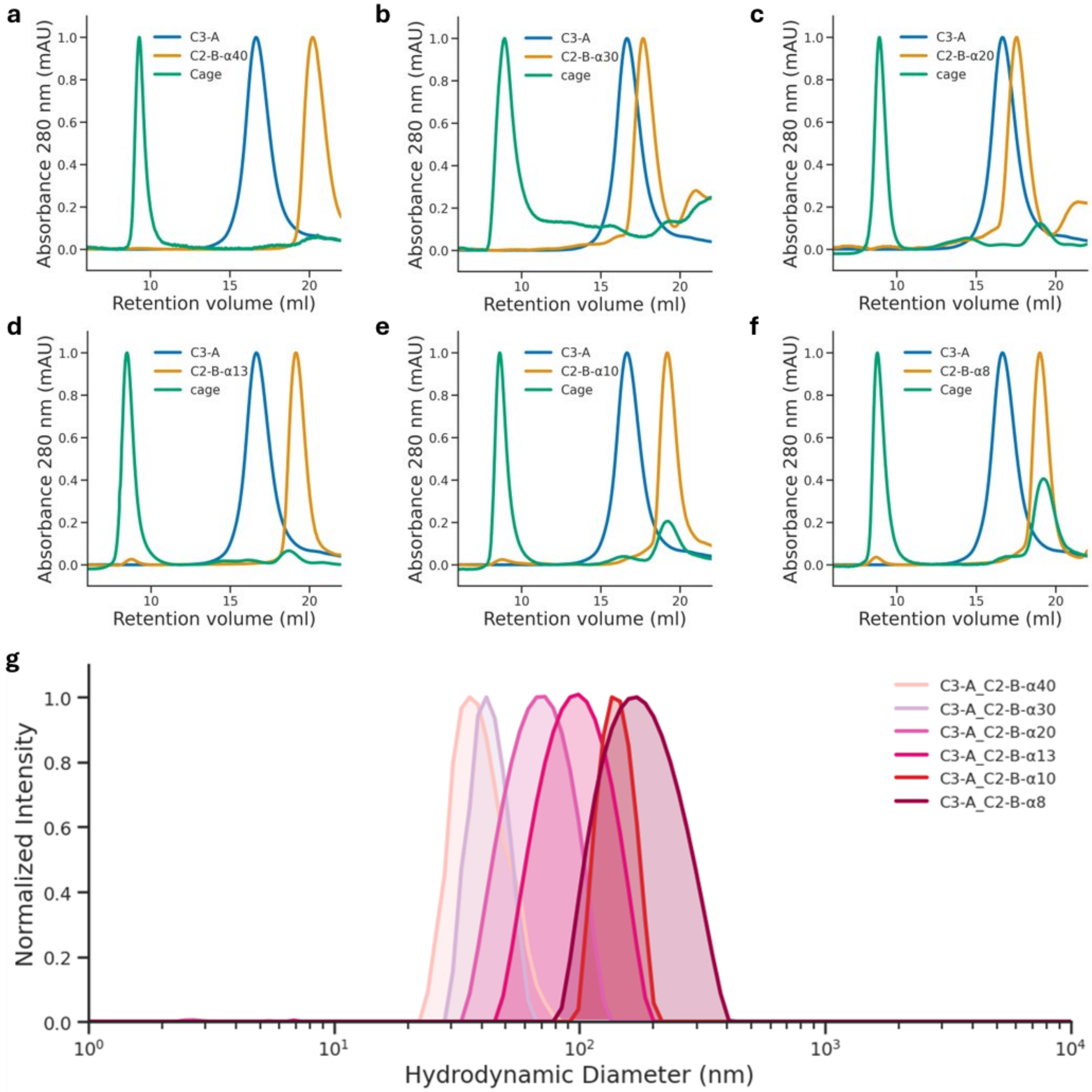

Fig. S1 | Characterization of cage assemblies with SEC and DLS. a-f) SEC elution profiles of in vitro assembled cages from equimolar of C3-A and different C2-B linkers. Assembly traces were eluted using Superose 6 Increase 10/300 GL AKTA columns, while building blocks were eluted with Superdex 200 Increase 10/300 GL AKTA columns. **g**) Dynamic light scattering profiles for each cage construct, showing their hydrodynamic size distributions in solution.

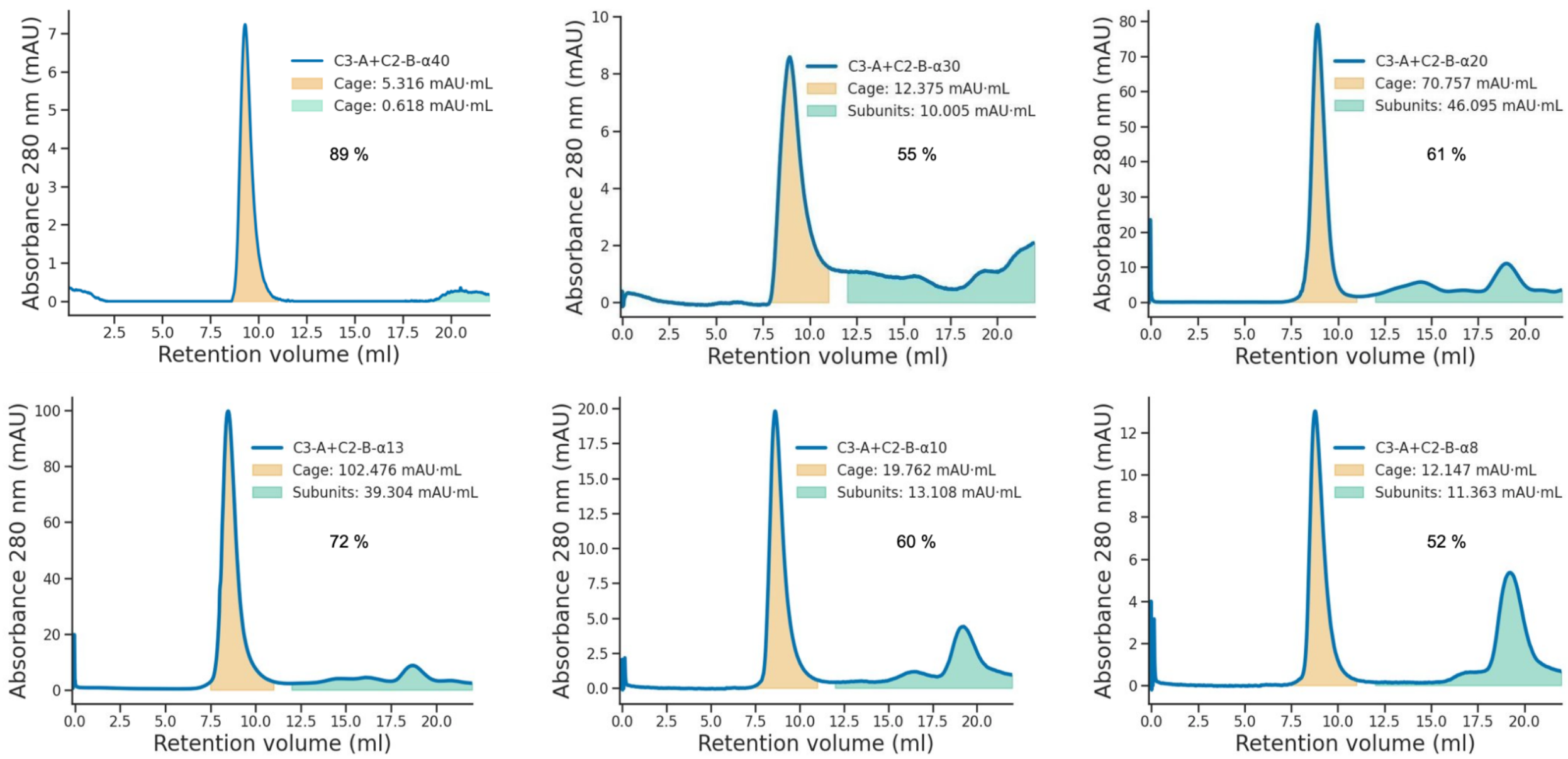

Fig. S2 | Quantification of in vitro cage assembly yields. SEC elution profiles of in vitro assembled unfunctionalized cages. Cage assembly yield, defined by AUC_{cage} / (AUC_{cage}+$AUC_{oligomer}$), was calculated from the cage peak (yellow, 7.5-11 mL) relative to the subunit/oligomeric region (teal, 12-22 mL).

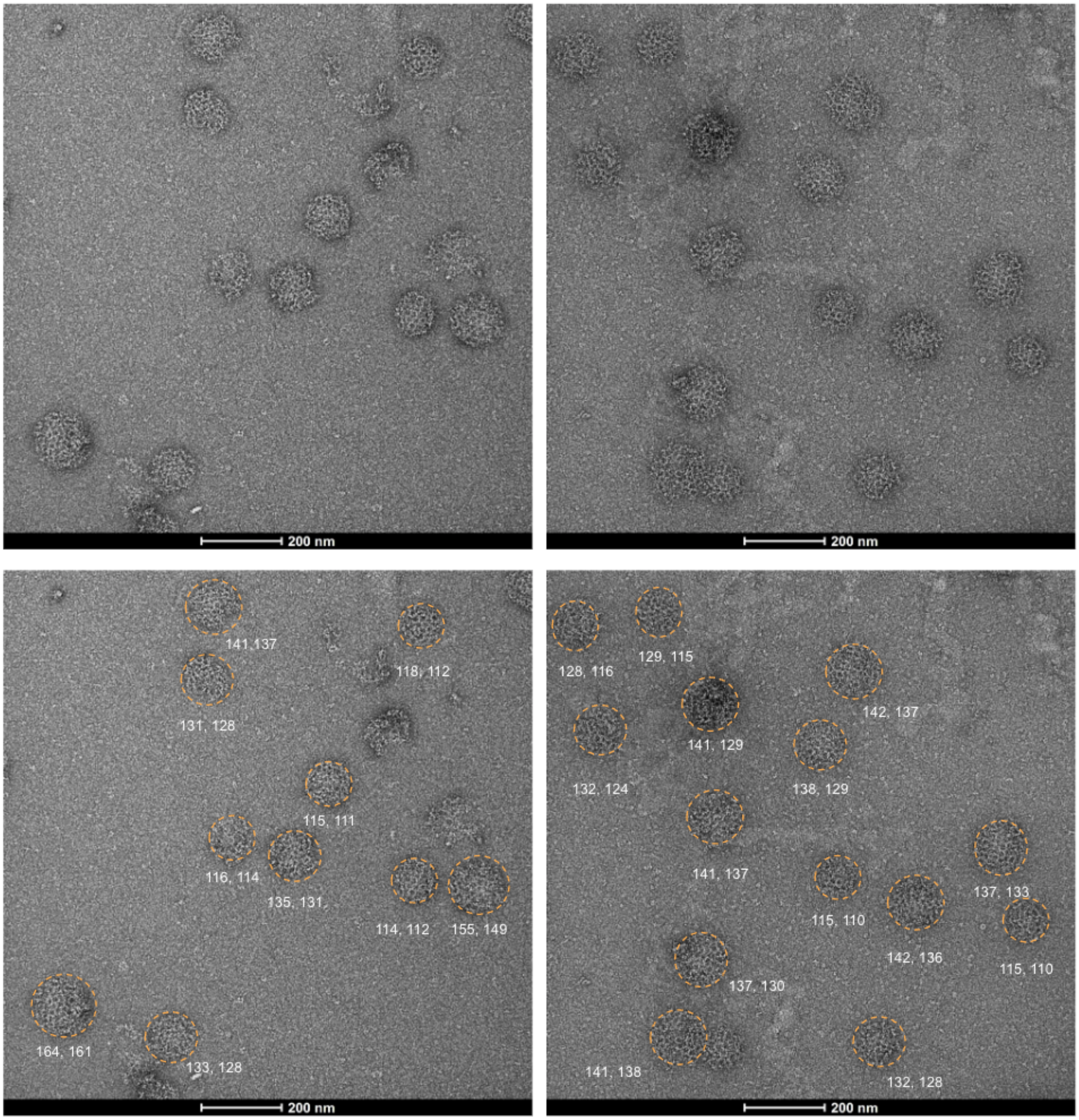

Fig. S3 | Electron microscopy characterization of T~16 cages. NsEM micrographs and morphology analysis for C3-A_C2-B-α10 (T~16) cages. Cages were manually selected and fitted with an ellipse in ImageJ. From these fits, the major (a) and minor (b) axes were extracted to calculate the aspect ratio (b/a) as a metric of shape uniformity.

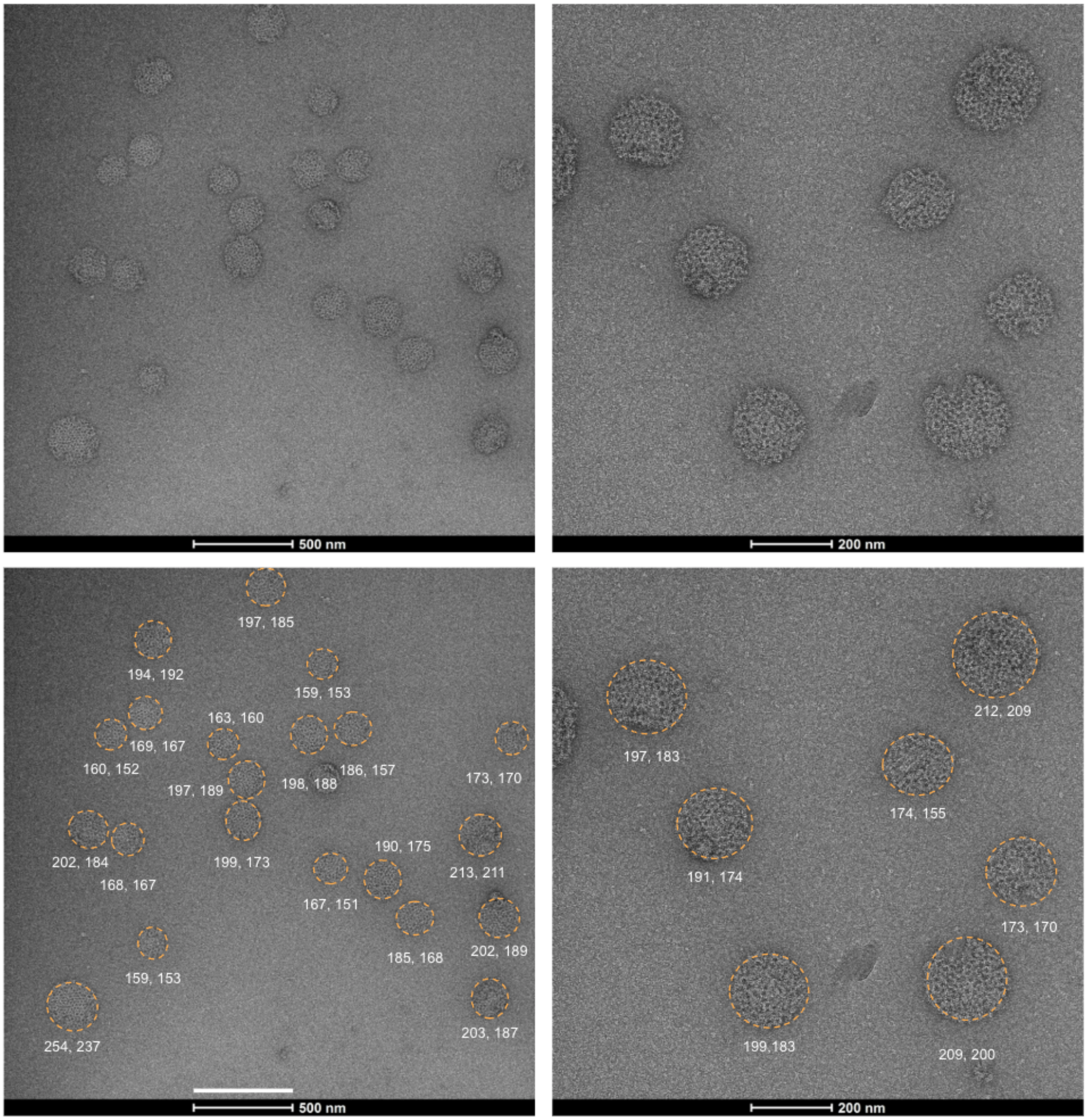

Fig. S4 | Electron microscopy characterization of T~25 cages. NsEM micrographs and morphology analysis for C3-A_C2-B-α8 (T~25) cage assemblies. Cages were manually selected and fitted with an ellipse in ImageJ. From these fits, the major (a) and minor (b) axes were extracted to calculate the aspect ratio (b/a) as a metric of shape uniformity.

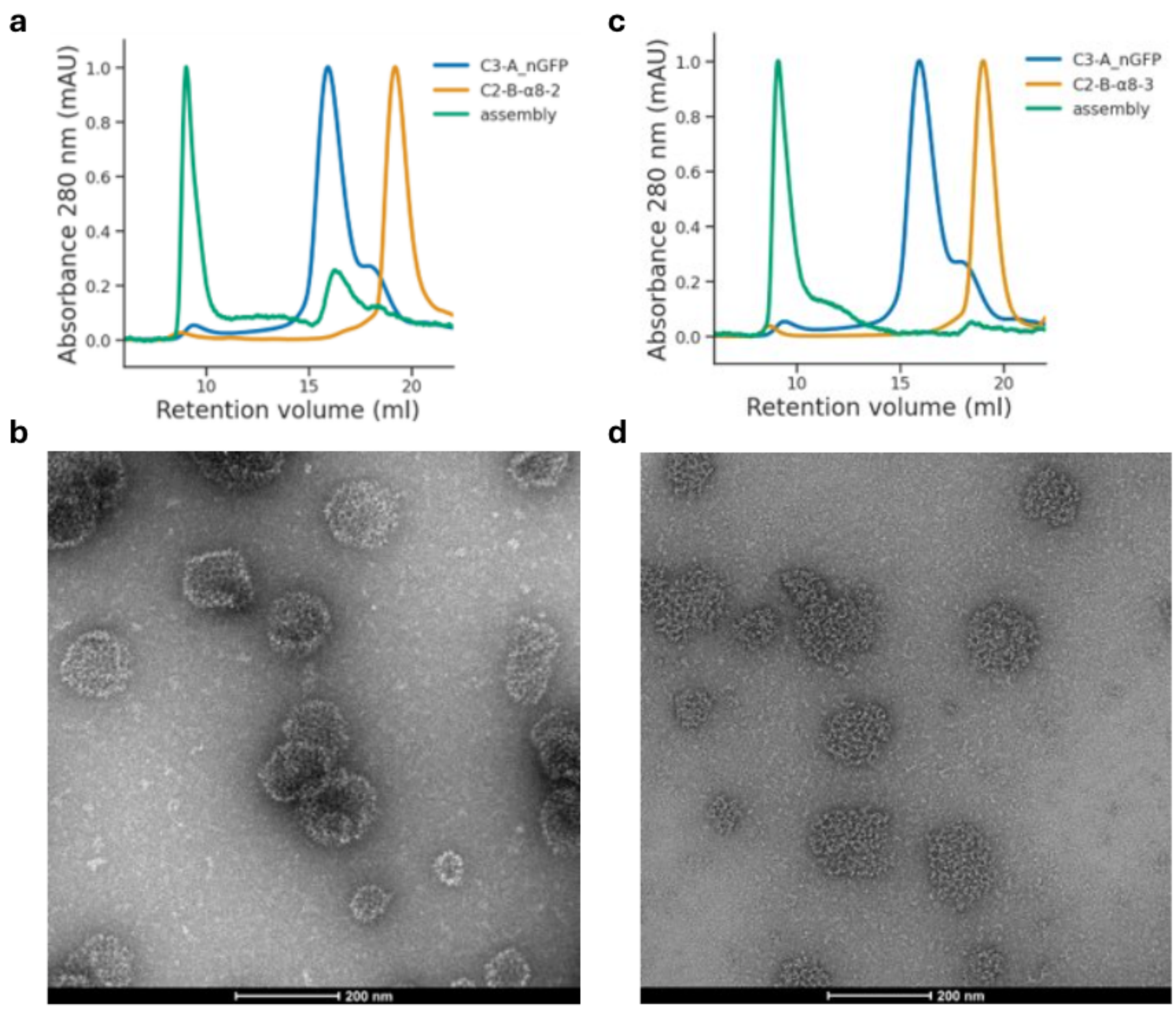

Fig. S5 | Characterization of C3-A_nGFP_C2-B-α8 cage variants. SEC profiles of C3-A_nGFP, individual C2-B-α8 variants (**a**: C2-B-α8-2, **c**: C2-B-α8-3), and their in vitro cage assemblies, respectively. **b,d**) Negative-stain EM images of the corresponding cage assemblies, revealing formation of 150 ~ 200 nm cages. Scale bar: 200 nm.

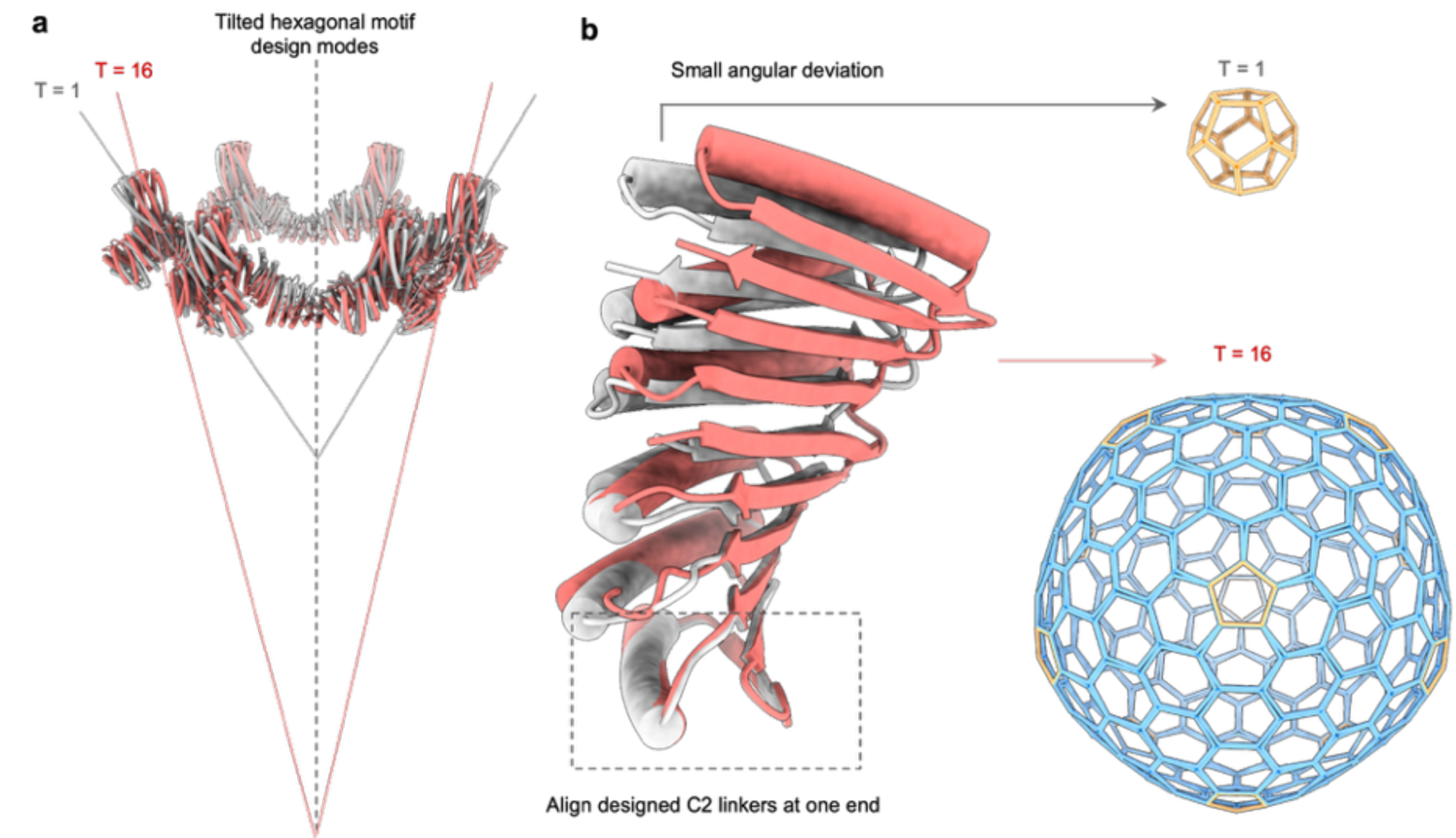

Fig. S6 | Impact of C-2B linker geometry on cage size. Aligning two hexagonal motifs along the z-axis shows a small angular deviation in C2-B linkers (C2-B-α40 vs. C2-B-α10) results in a significant change in cage triangulation number (T ~ 1 vs. T ~ 16).

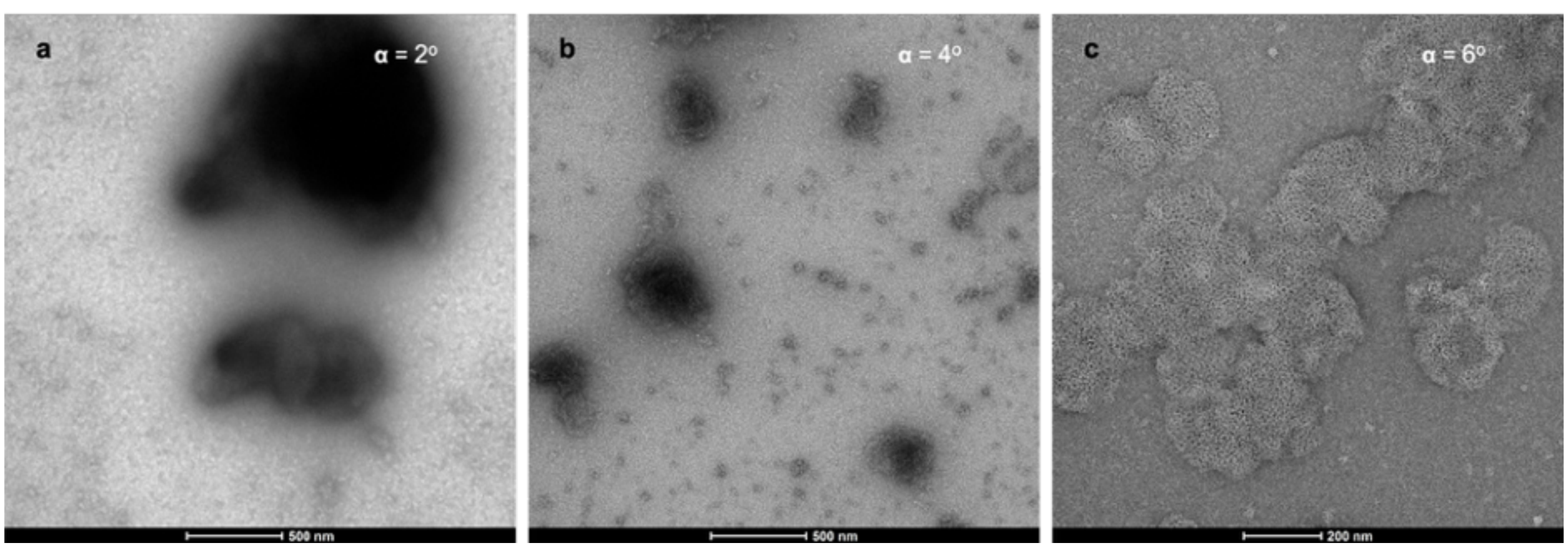

Fig. S7 | Aggregated assemblies of low-cone-angle linkers. Representative electron microscopy micrographs showing aggregated assemblies formed with C2-B linkers targeting low cone angles: **α** = 2° (a), 4° (b), and 6° (c).

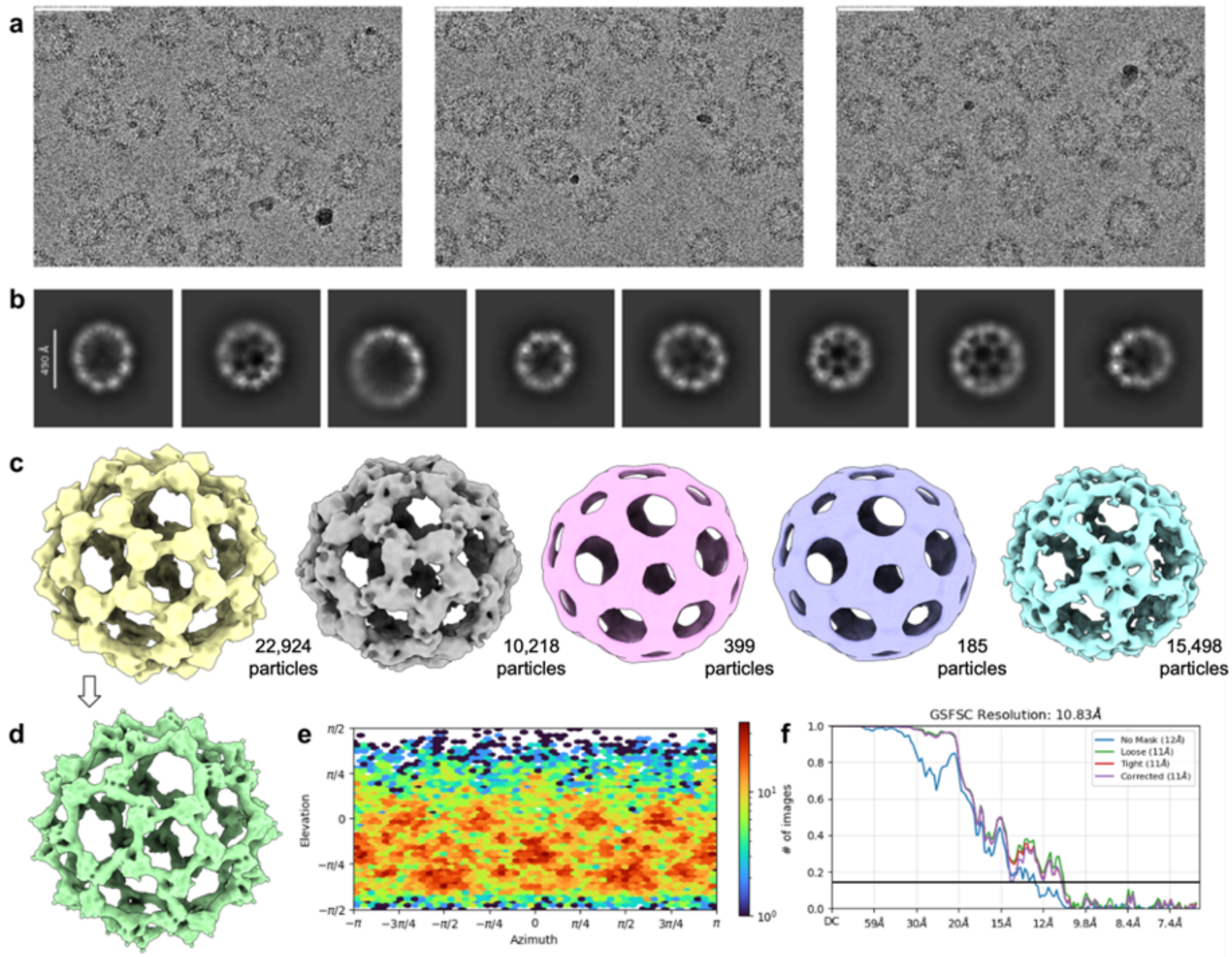

Fig. S8 | Single-particle CryoEM of T ~ 3 cage. **a**) Representative micrographs, 100 nm scale bar. **b**) 2D class averages, 43,349 particles. **c**) ab initio generated with 5 volumes: yellow 22,924 particles, gray 10,218 particles, pink 399 particles, purple 185 particles, cyan 15,498 particles **d**) Non-Uniform Refinement volume green, generated with the yellow ab initio volume and 22,924 particles from that volume. **e**) Orientational distribution plot demonstrating full angular sampling f, Global FSC non uniform refinement.

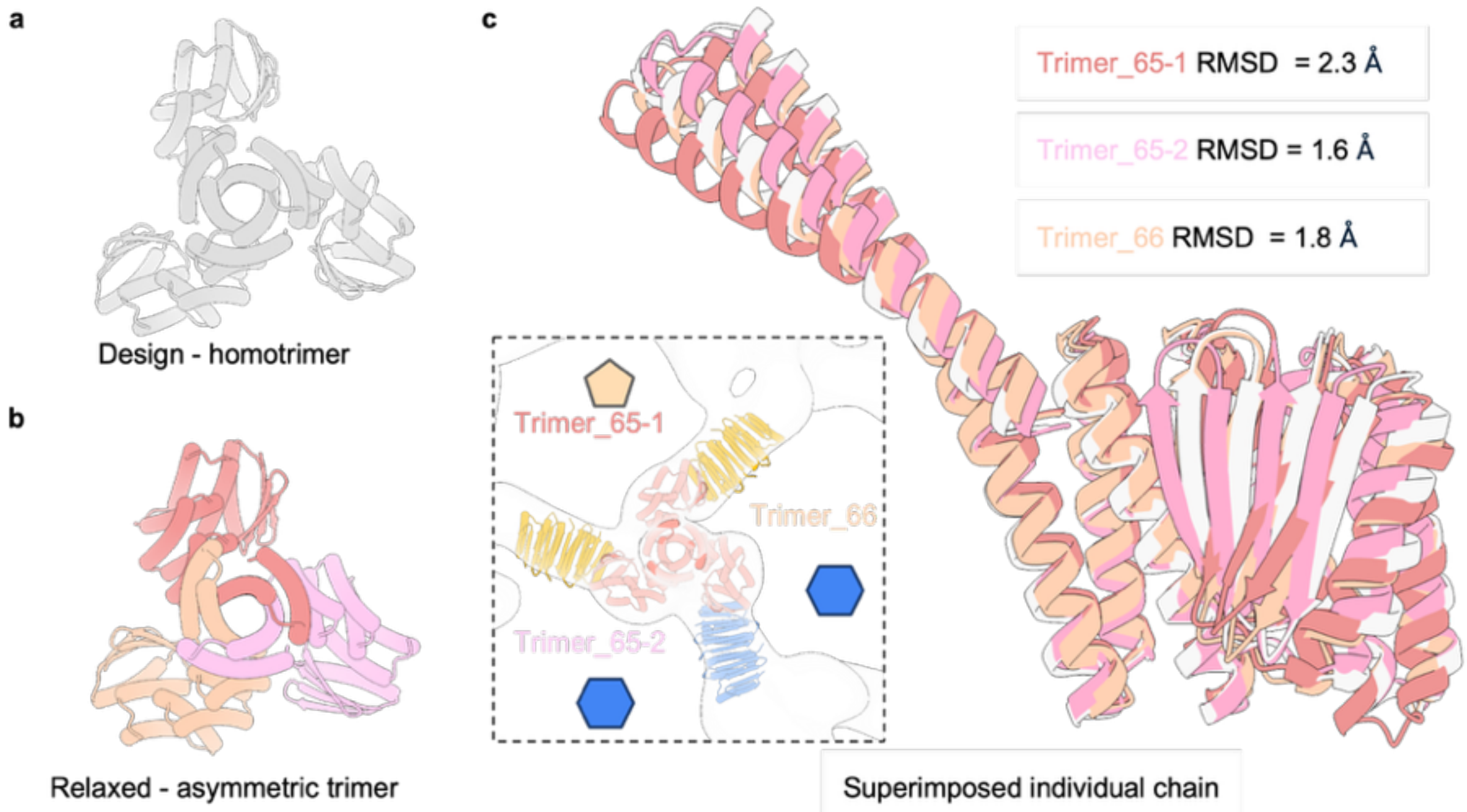

Fig. S9 | Symmetry breaking of C3-A during cage assembly. **a**) Top-down view of a design model of the C3-A homotrimer. **b**) Relaxing the design model into the cryoEM density generates an asymmetric trimer with individual chains adopting slightly different conformations. **c**) Superposition of three different chains (colored) show deviation from the design model (grey) with ~ 2 Å RMSD.

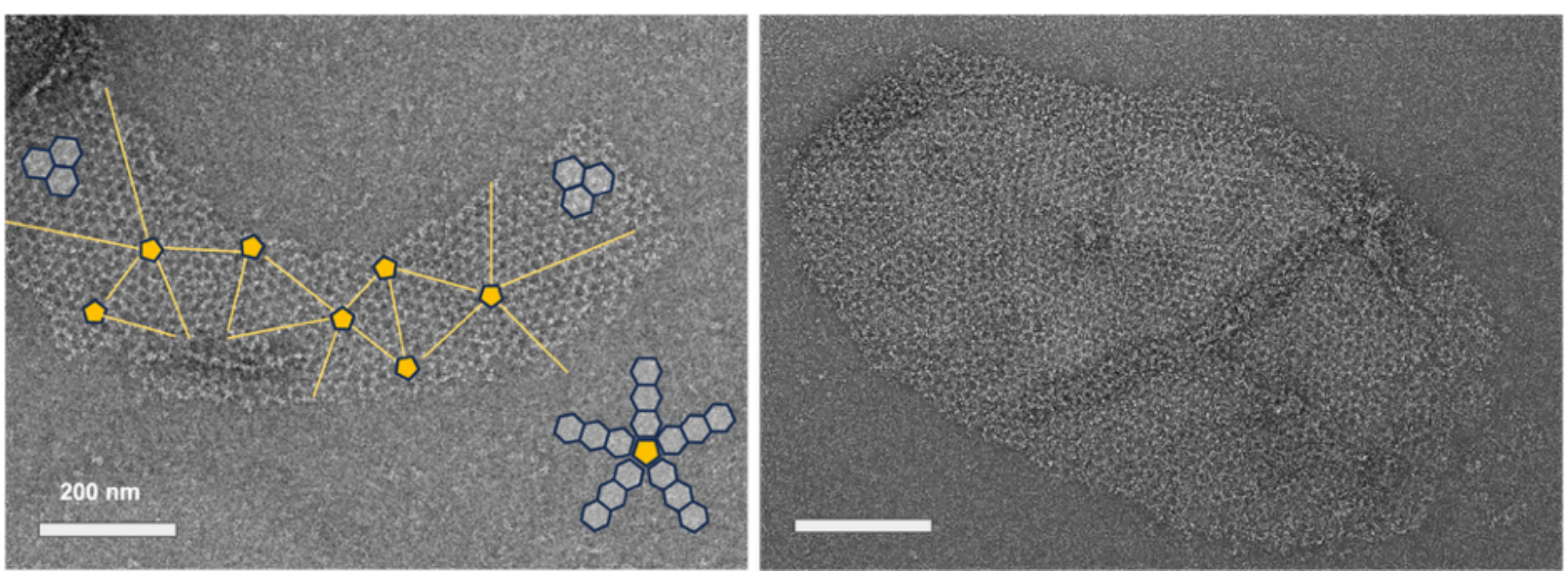

Fig. S10 | Defect distribution within high-T cages. Non-uniform spatial distribution of pentagonal defects observed in nsEM micrographs of large cages collapsed on the EM grid from in vitro assembled C3-A_L267Q-variant + C2-B-α8 linker.

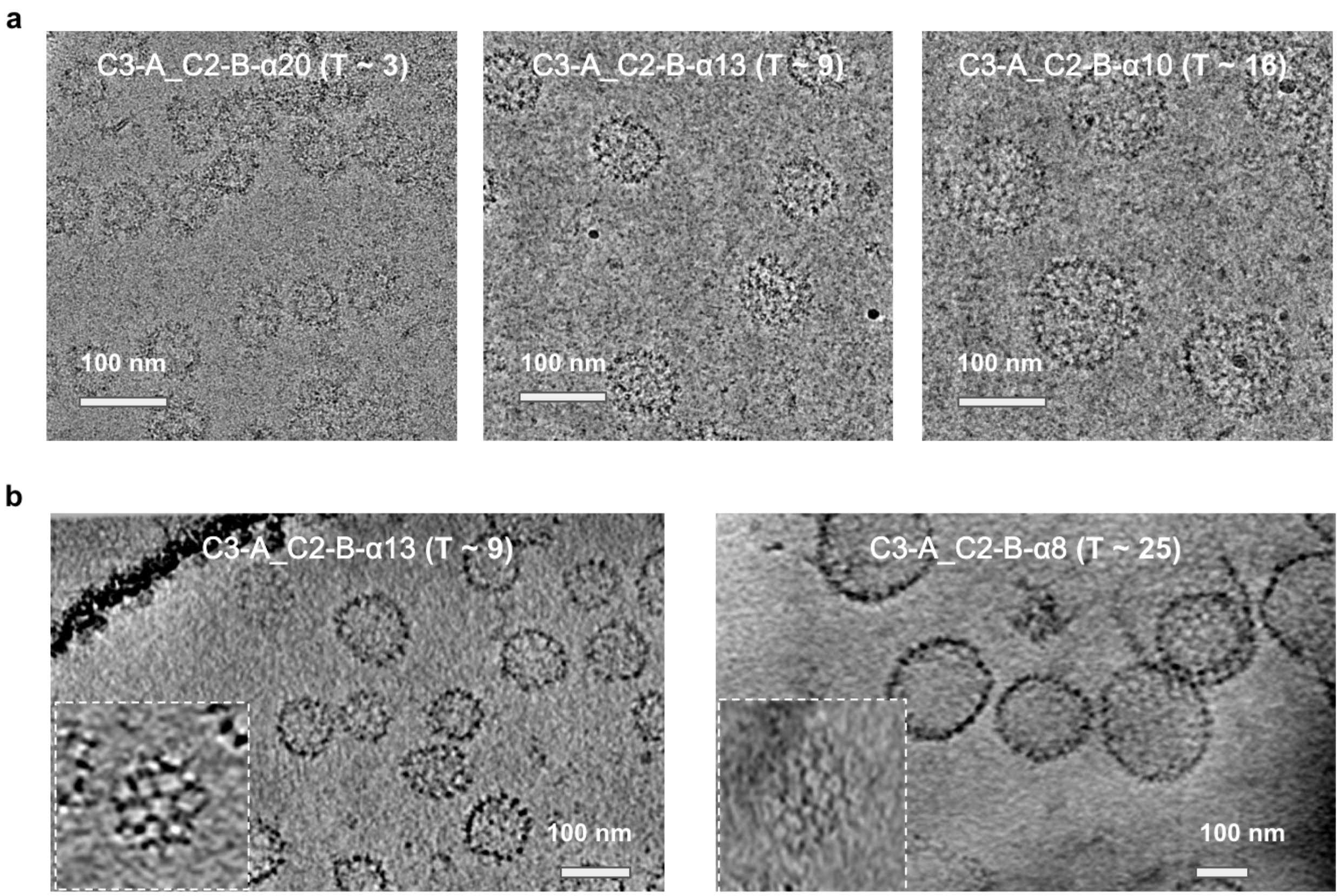

Fig. S11 | CryoEM imaging of protein cages. **a**) CryoEM micrographs of in vitro assembled cages. **b**) Additional cryoET tomograms and zoomed in view of tomogram slices for individual cage (inset).

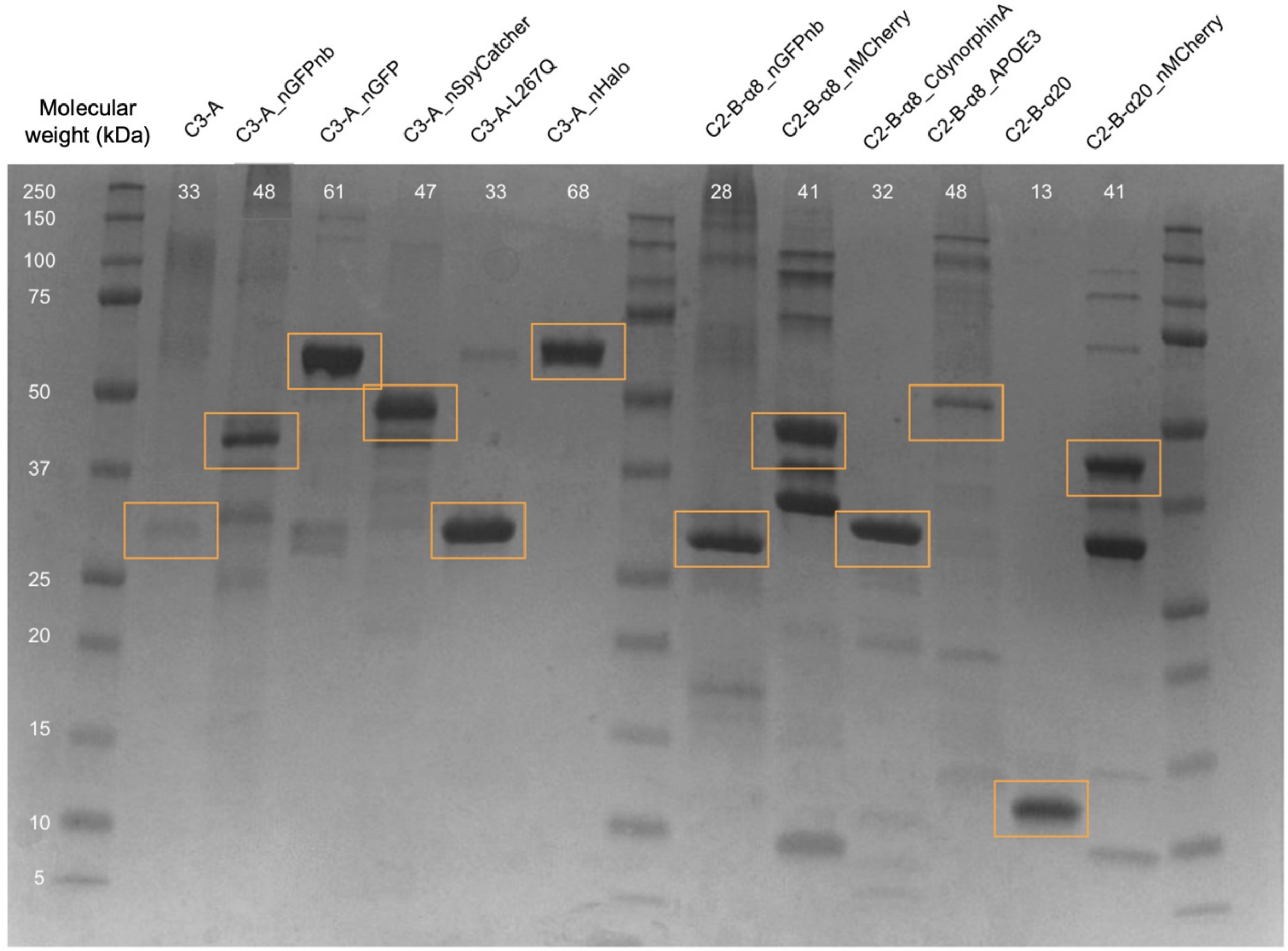

Fig. S12 | SDS–PAGE analysis of representative functionalized building blocks. All constructs exhibit bands at the expected molecular weights (top row, values in kDa shown below each construct name). Minor degradation products were observed for C2-B-α20_nMCherry and C2-B-α8_nMCherry.

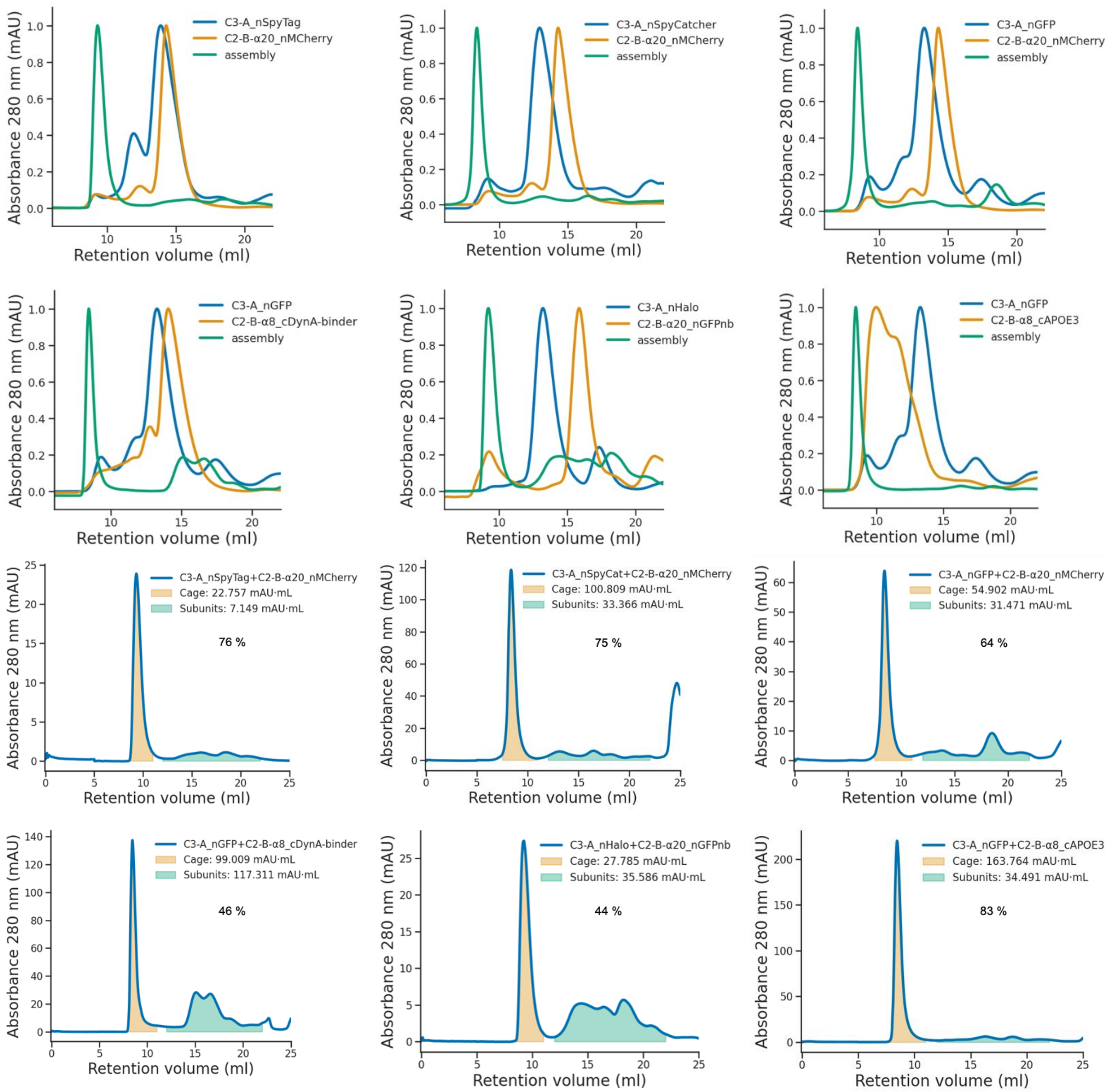

Fig. S13 | SEC characterization of functionalized cages and their components. Top panel: Assembly traces were eluted using Superose 6 Increase 10/300 GL AKTA columns, while building blocks were eluted with Superdex 200 Increase 10/300 GL AKTA columns. Bottom panel: Cage assembly yield, defined by AUC_{cage} / (AUC_{cage}+$AUC_{oligomer}$), was calculated from the cage peak (yellow, 7.5-11 mL) relative to the subunit/oligomeric region (teal, 12-22 mL).

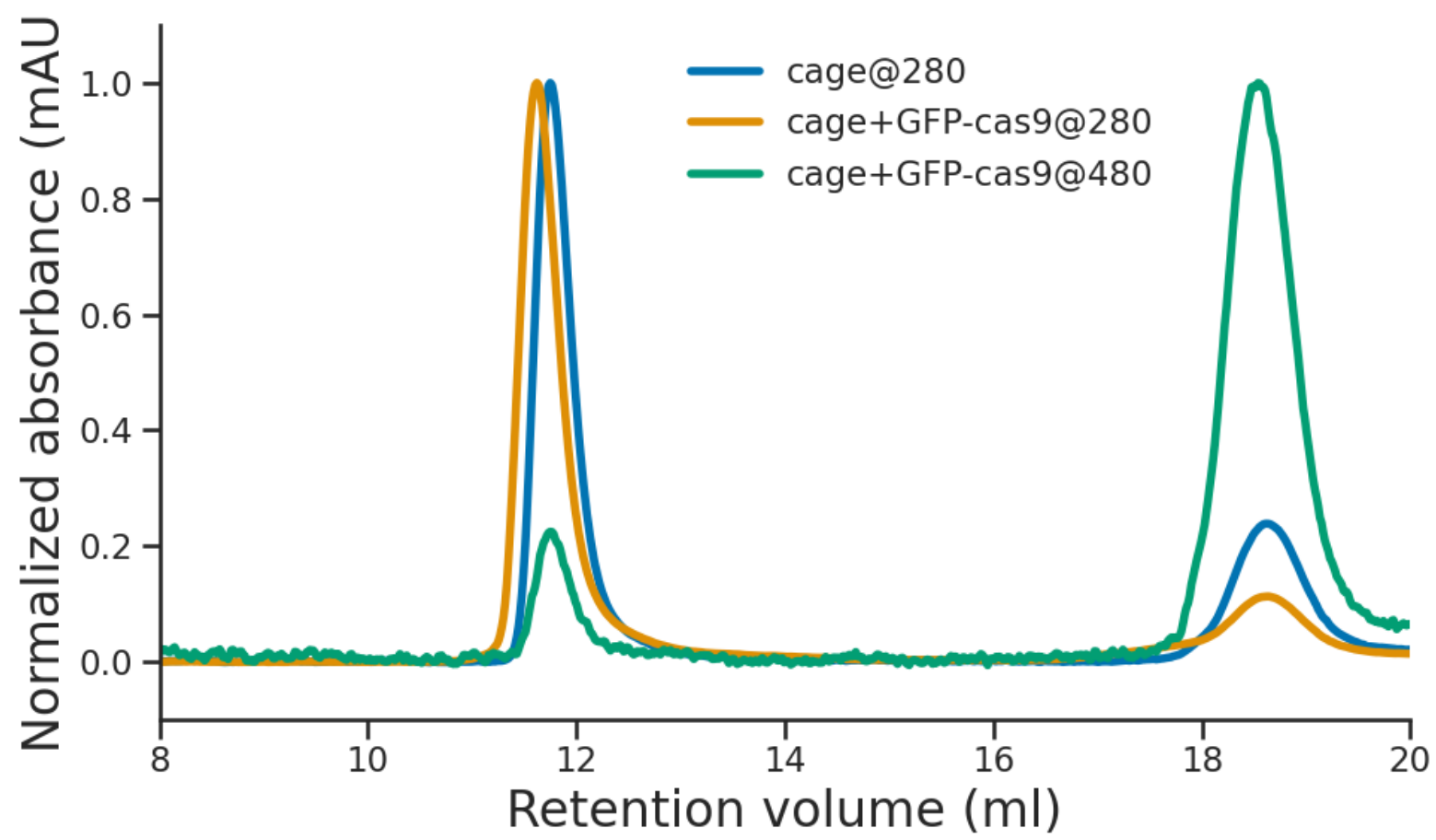

Fig. S14 | SEC validation of Cas9-GFP encapsulation. Size-exclusion chromatography trace showing co-elution of the GFPnb-cage assembly with GFP fluorescence from Cas9-GFP, indicating successful in vitro cargo encapsulation.

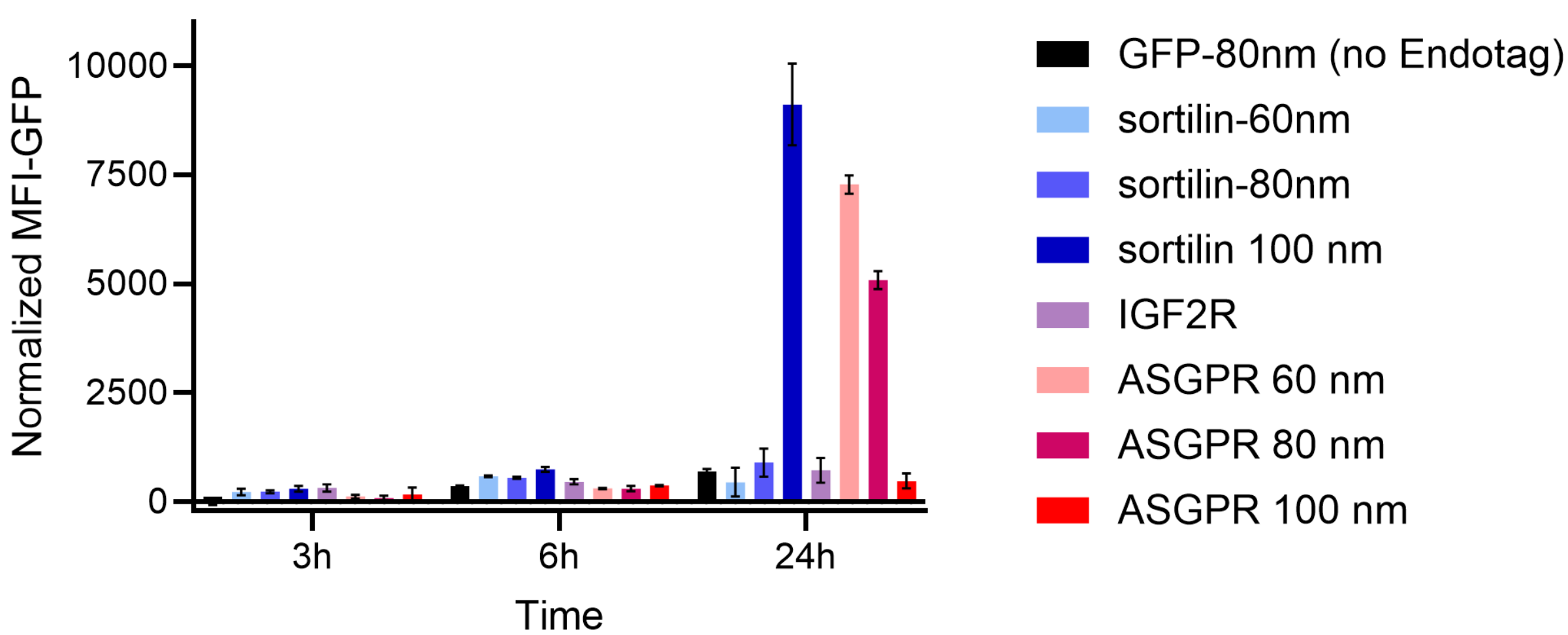

Fig. S15 | Cellular uptake kinetics of EndoTag-functionalized cages. Flow-cytometry quantification of GFP signal in HEP3B cells incubated with cages of different diameters (60, 80, 100 nm) functionalized with Sortilin-, IGF2R-, or ASGPR-targeting EndoTags. Uptake increases over time for all targeting modalities, with the highest internalization observed at 24 h. Receptor-specific differences dominate the uptake profiles, and size-dependent trends vary across receptors. Error bars represent mean ± s.d. of biological replicates.

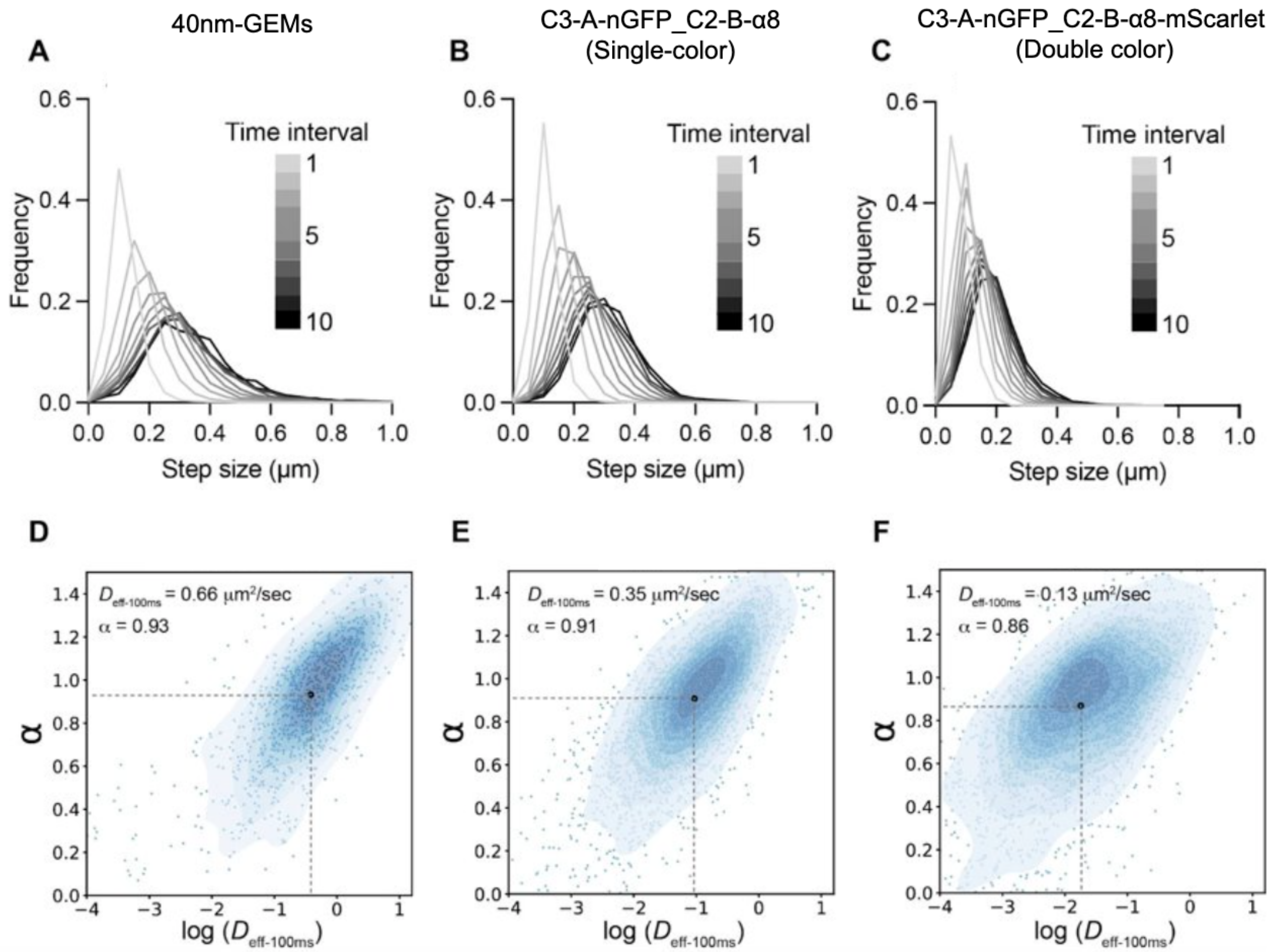

Fig. S16 | Characterization of passive rheological cage probes in the cytosol. a-c) Step size distribution of 40nm-GEMs, single-, and dual-color C2-B-α10 cages at the indicated time intervals. **d-f**) Effective diffusivity at 100 ms ($D_{eff\text{-}100ms}$), log10 scales were plotted against the anomalous exponent (α) for individual trajectories for 40nm-GEMs, single-color cage and dual-color cage. The color map represents the Kernel Density Estimation (KDE) which accounts for the spatial density of the data points. Darker shades in the KDE represent a higher concentration of data points around that value. The central point in each graph represents the median value of $D_{eff\text{-}100ms}$ and α for each type of cage.

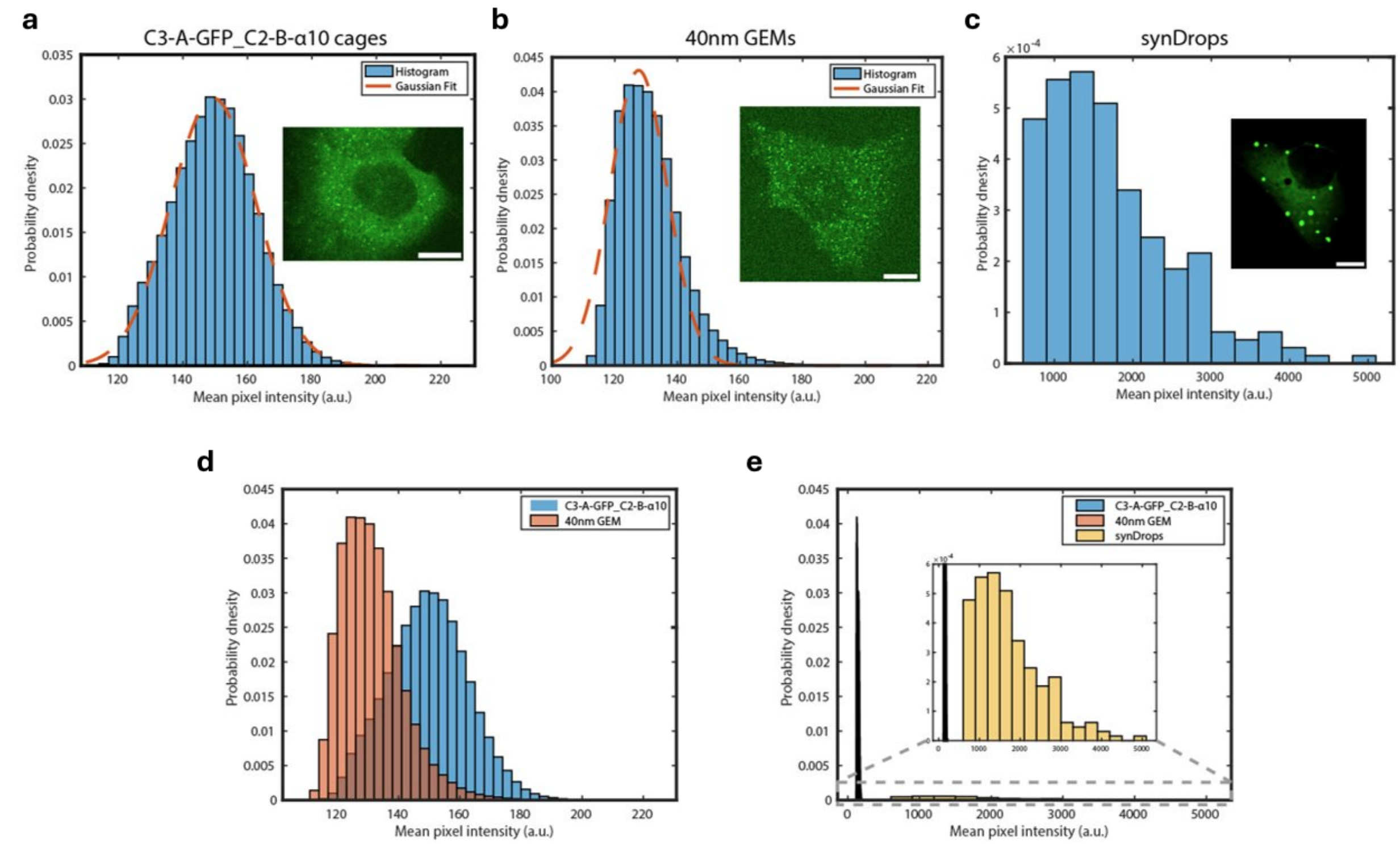

Fig. S17 | Comparison of intracellular cages and condensate formation. **a**) Representative fluorescence images of U2OS cells expressing single-color C3-A_nGFP_C2-B-α10 cage construct (this study), **b**) 40 nm-GEMs (control particles), and **c**) synDrops (control two-component condensates). Particles were detected with TrackMate (Fiji) using identical spot-detection settings across conditions. Scale bars, 10 µm. **d**) Distributions of mean particle pixel intensity extracted by TrackMate for each sample. Both C3-A_nGFP_C2-B-α10 cages and 40 nm-GEMs exhibit narrow, symmetric histograms well fit by Gaussians ($R^2 > 0.97$), whereas synDrops show a right-skewed distribution with a long tail. **e)** Summary statistics of particle intensities highlight the tighter spread for C3-A_nGFP_C2-B-α10 cages and 40 nm-GEMs versus the broad, heterogeneous condensates formed by synDrops.

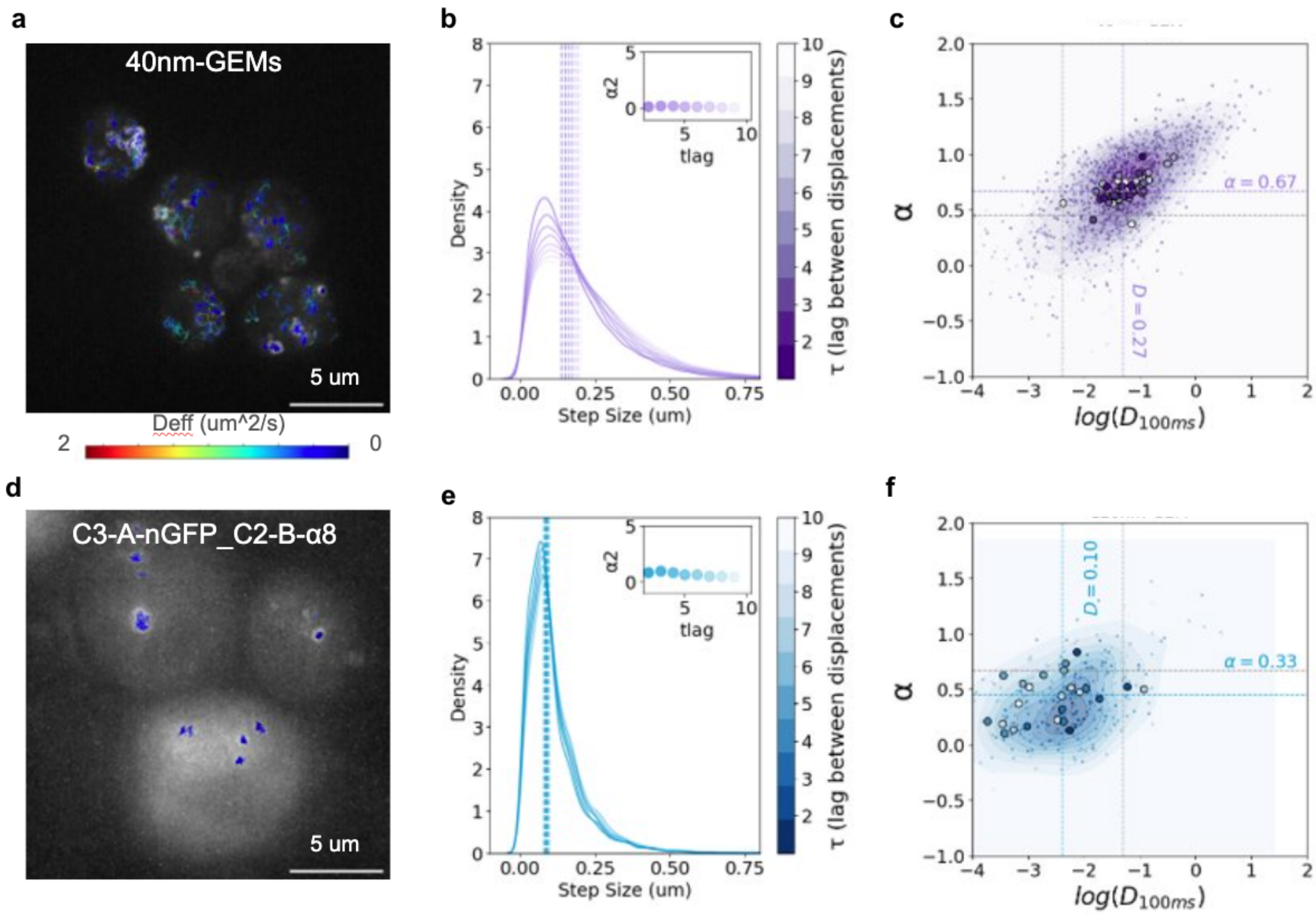

Fig. S18 | Passive rheological probes in the *S. cerevisiae* cytosol. Representative bright field images of 40nm-GEMs (**a**) and C3-A-nGFP_C2-B-α8 (**d**) in *S. cerevisiae* yeast cells. Step size distribution of 40nm-GEMs (**b**) and C3-A-nGFP_C2-B-α8 (**e**) at the indicated time intervals (inset). Effective diffusivity at 100 ms ($D_{eff\text{-}100ms}$), log10 scales were plotted against the anomalous exponent (α) for individual trajectories for 40nm-GEMs (**c**) and C3-A-nGFP_C2-B-α8 (**f**) The color map represents the Kernel Density Estimation (KDE) which accounts for the spatial density of the data points. Darker shades in the KDE represent a higher concentration of data points around that value. The central point in each graph represents the median values of $D_{eff\text{-}100ms}$ and α for each type of cage.

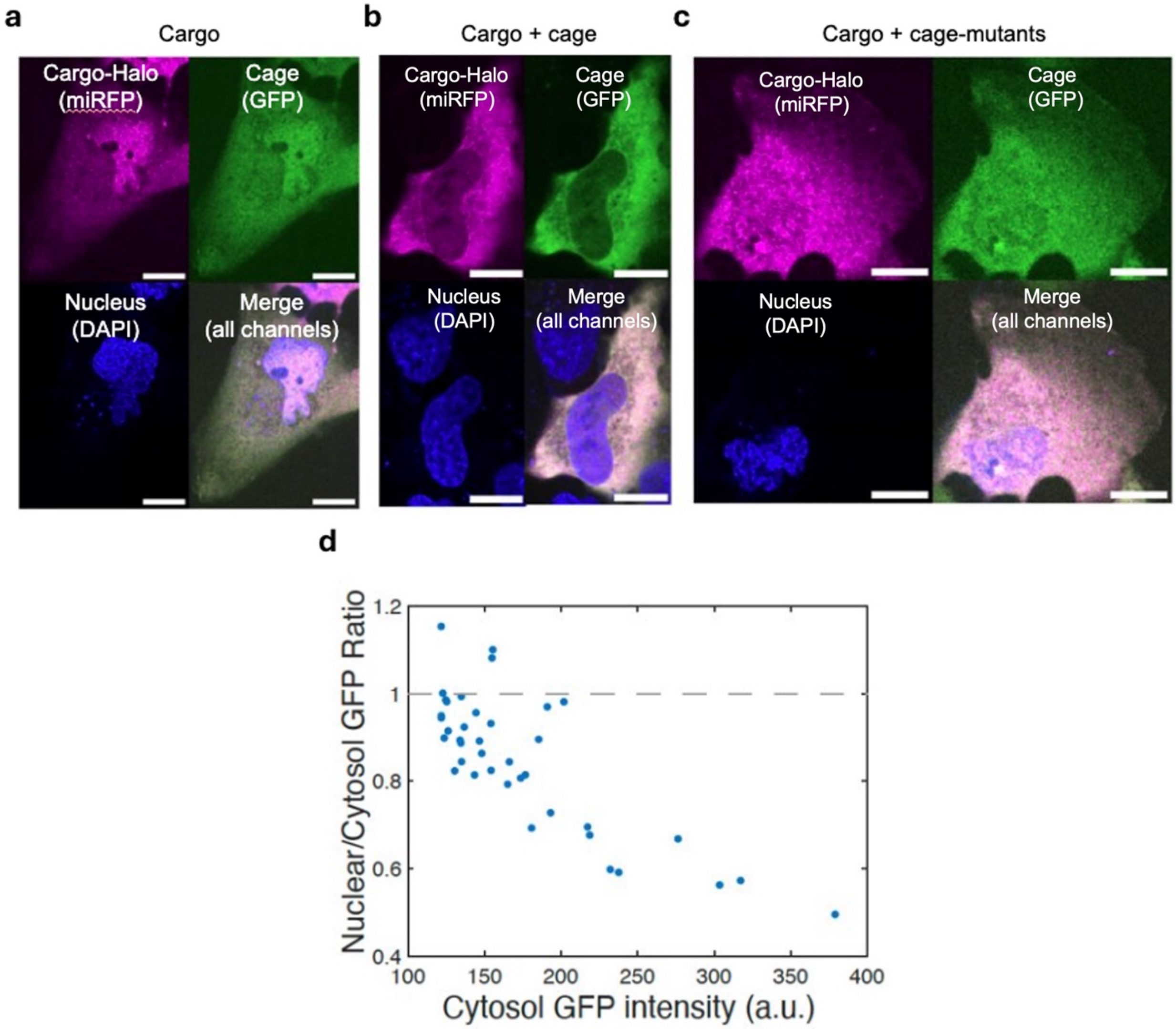

Fig. S19 | Mutations at the assembly interface impair cage assembly. To directly test whether p53 relocalization is dependent on cage assembly, we disrupted the A–B interface by substituting C3-A Leu267 with polar residues (L267K and L267R), which markedly reduced particle formation. **a**) U2OS cells expressing p53-HaloTag-GFP-nanobody cargo alone show predominantly nuclear p53 due to its NLS signal. **b**) Co-expression of C3-A_nGFP_C2-B-α10 cages with the cargo construct redistributes p53 to the cytosol, consistent with binding to the bulky cages impairing nuclear import. **c**) Interface mutations in C3-A (L267K or L267R) disrupt cage assembly and produce mixed nuclear and cytosolic p53 localization. **d**) Quantification of compartmentalization shows that the GFP nucleus-to-cytosol (N/C) ratio decreases with increasing cytosolic GFP (greater cytosolic enrichment of the components). In assembly-defective mutants, p53 relocalization is attenuated relative to assembled cages (higher p53 N/C at comparable expression), indicating that intact cage assembly is required for efficient p53 relocalization from the nucleus to cytosol. Scale bars, 10 μm.

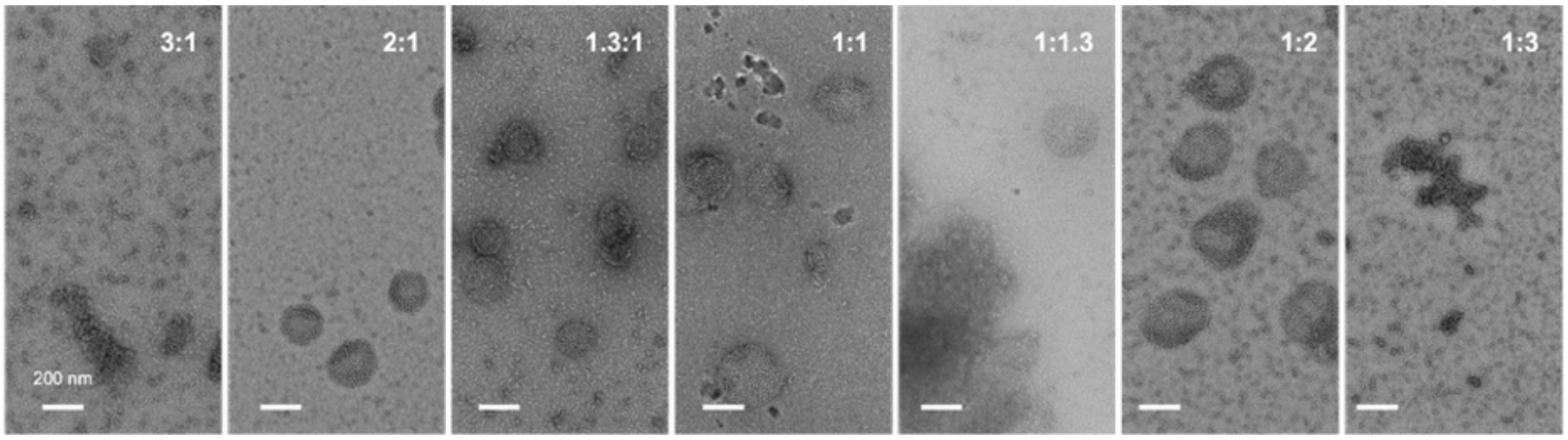

Fig. S20 | Subunit stoichiometry influences in vitro cage assembly. NsEM characterization of C3-A_C2-B-α8 cages assembled at different subunit stoichiometry. Mixing at high molar excess (3:1 or 1:3; normalized to 20 μM) disrupts cage formation, whereas near-equimolar ratios support intact assemblies.

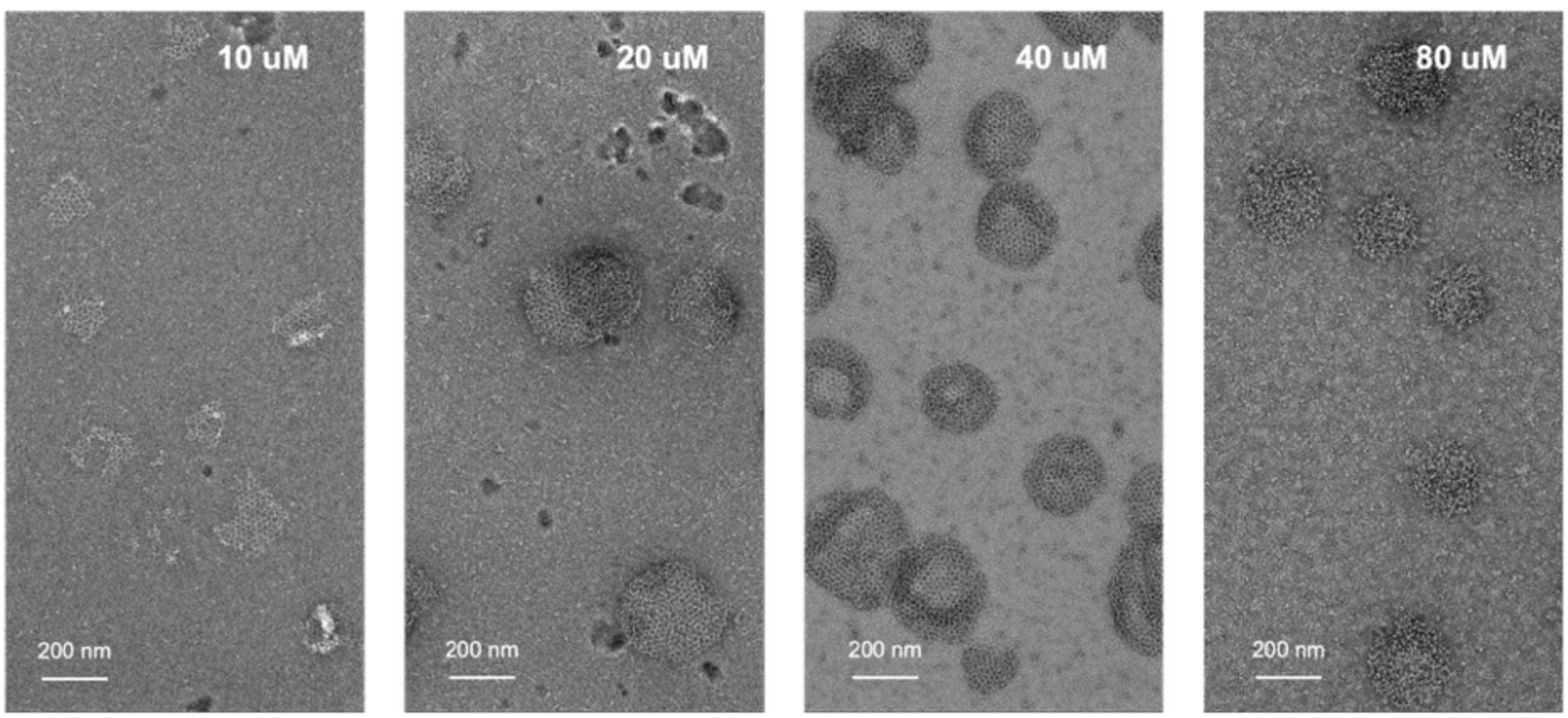

Fig. S21 | Subunit concentration influences in vitro cage assembly. In vitro C3-A_C2-B-α8 (T~25) cage assembly at different building block concentrations. Equimolar of purified C3-A and C2-B-α8 linkers in standard buffers were mixed at room temperature at monomer concentration specified above. Partial cage fragments are observed at ≤ 10 μM, while concentrations ≥ 120 μM show transient aggregation upon mixing that gradually resolves at elevated temperature.

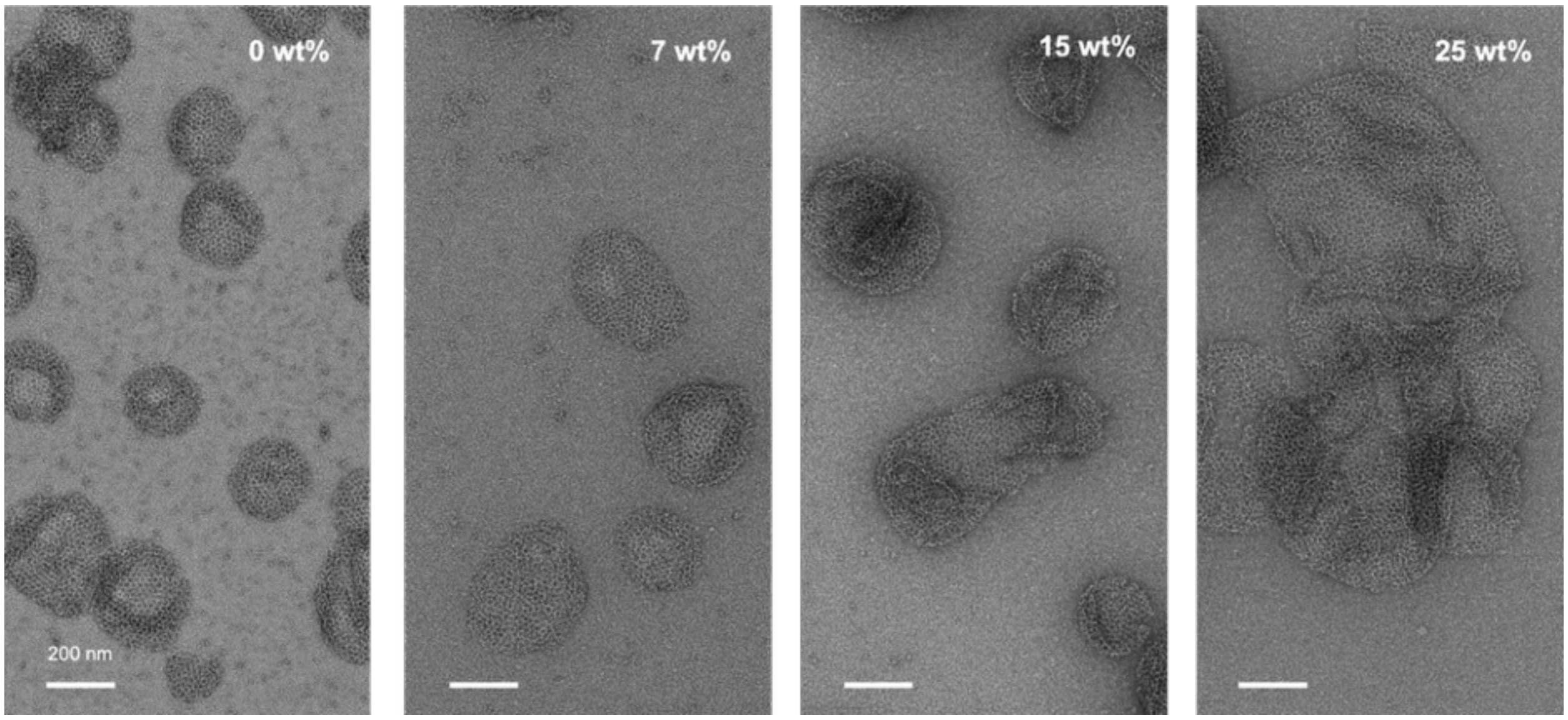

Fig. S22 | Glycerol concentration modulates in vitro cage assembly. Glycerol concentration modulates in vitro C3-A_C2-B-α8 (T~25) cage assembly size. Increasing glycerol, and thus solution viscosity, modulates apparent cage size and assembly morphology.

Supplementary Table S1. Summary of T numbers, predicted cage sizes, and molecular weights.

T number	Radius-edge ratio	Predicted diameter (nm)	Number of subunits	Molecular weight (MDa)
1	1.4	36	120	2.4
3	2.5	65	360	7.2
4	2.9	75	480	9.6
7	3.8	100	840	17
9	4.11	110	1080	22
12	4.9	127	1440	29
16	5.7	150	1920	38
21	6.5	170	2520	50
25	7.2	190	3000	60
36	8.5	220	4320	86

Supplementary Table S2. Purification yields of protein cage building blocks.

Construct	SEC purified yield (mg / 50-mL-culture)
C3-A	9
C2-B-α40	6
C2-B-α30	4
C2-B-α20	8
C2-B-α13	5
C2-B-α10	3
C2-B-α8	4
C3-A_nGFP	6
C3-A_nSpyCatcher	2
C3-A_nSpyTag	8
C2-B-α20_nGFPnb	1
C2-B-α8_cDynA-binder	2
C2-B-α20_nMCherry	6
C2-B-α8_nMCherry	5
C2-B-α8_cAPOE3	1

Supplementary Table S3. In vitro cage assembly yields of representative constructs.

C3 component	C2 component	Cage assembly yield (%)
	**Unfunctionalized cages**	
C3-A	C2-B-α40	89
C3-A	C2-B-α30	55
C3-A	C2-B-α20	61
C3-A	C2-B-α13	72
C3-A	C2-B-α10	60
C3-A	C2-B-α8	52
	**Functionalized cages**	
C3-A_nSpyTag	C2-B-α20_nMCherry	76
C3-A_nGFP	C2-B-α20_nMCherry	75
C3-A_nGFP	C2-B-α8_cDynA-binder	64
C3-A_nSpyCatcher	C2-B-α20_nMCherry	46
C3-A_nHalo	C2-B-α20_nGFPnb	44
C3-A_nGFP	C2-B-α8_cAPOE3	83

Supplementary Table S4. Plasmids and cell lines.

**Recombinant DNA**	**Source**	**Identifier**
pCMV-40nm-GEMs-GFP	Holt lab	pLH1196
pCMV-GFP-C3-P2A-C2-B-α13	Holt lab	pLH2599
pCMV-GFP-C3-P2A-B4	Holt lab	pLH2600
pCMV-GFP-C3-P2A-C2-B-α10	Holt lab	pLH2601
pCMV-GFP-C3-P2A-C2-B-α10-mScarlet	Holt lab	pLH2704
pCMV-GFP-C3-A-L267K-P2A-C2-B-α10	Holt lab	Point mutation
pCMV-GFP-C3-A-L267R-P2A-C2-B-α10	Holt lab	Point mutation
pMD2.G	Addgene #12259	pLH1863
psPAX2	Addgene #12260	pLH1864
pCMV-p53-HaloTag-GFP-nanobody	Baker lab	pSW-cargo-1
pCMV-p53-GFP-nanobody-HaloTag	Baker lab	pSW-cargo-2
**Cell lines**	**Source**	
Human U2OS cell	Holt lab	
U2OS pCMV-GFP-C3-P2A-C2-B-α10-mScarlet	Holt lab	
U2OS pCMV-GFP-C3-P2A-C2-B-α10	Holt lab	
Human HEP3B cell	Baker lab	

Supplementary Table S5. Sequences of designed protein constructs.
* His-tag for Ni-NTA purification, Trp-tag for A280 absorbance, GS flexible linker, mutation
* N terminal Methionine is not included in ordered sequences

Constructs	Protein Sequences*
C3-A	SDEAIKRLLEELRASTAELKRATASLRAITEELKKNPSEDALVEHNRAIVEHNA<br>IIVENNRIIAAVLEAIVAEFILAKARLLVWEARKKGNPEEVLEAAKKALRVAEEA<br>AKAGIEAIFKAAAEAALEIAMRLIDLATKKGDPDLVREAQKVAERVAELADKN<br>GDEEVKLKAYAAAVAAEIIRRIETLKRSGSSYEDIRQTLRETLERIIEELKRRGV<br>DSSQIVWAIIYVAVAVMGVTMETHKSGNEVKVVIKGLHESQQEELLELVLRAA<br>ELAGVRVRIRFKGDTVTIVVRGLEHHHHHH
C3-A_nGFP	MHHHHHHGSSKGEELFTGVVPILVELDGDVNGHKFSVRGEGEGDATNGKL<br>TLKFICTTGKLPVPWPTLVTTLTYGVQCFARYPDHMKQHDFFKSAMPEGYV<br>QERTISFKDDGTYKTRAEVKFEGDTLVNRIELKGIDFKEDGNILGHKLEYNFN<br>SHNVYITADKQKNGIKANFKIRHNVEDGSVQLADHYQQNTPIGDGPVLLPDN<br>HYLSTQSVLSKDPNEKRDHMVLLEFVTAAGITHGMDELYGSGSGSGSGSGS<br>GSGSSDEAIKRLLEELRASTAELKRATASLRAITEELKKNPSEDALVEHNRAIV<br>EHNAIIVENNRIIAAVLEAIVAEFILAKARLLVWEARKKGNPEEVLEAAKKALRV<br>AEEAAKAGIEAIFKAAAEAALEIAMRLIDLATKKGDPDLVREAQKVAERVAELA<br>DKNGDEEVKLKAYAAAVAAEIIRRIETLKRSGSSYEDIRQTLRETLERIIEELKR<br>RGVDSSQIVWAIIYVAVAVMGVTMETHKSGNEVKVVIKGLHESQQEELLELV<br>LRAAELAGVRVRIRFKGDTVTIVVRG
C3-A-L267Q	SDEAIKRLLEELRASTAELKRATASLRAITEELKKNPSEDALVEHNRAIVEHNA<br>IIVENNRIIAAVLEAIVAEFILAKARLLVWEARKKGNPEEVLEAAKKALRVAEEA<br>AKAGIEAIFKAAAEAALEIAMRLIDLATKKGDPDLVREAQKVAERVAELADKN<br>GDEEVKLKAYAAAVAAEIIRRIETLKRSGSSYEDIRQTLRETLERIIEELKRRGV<br>DSSQIVWAIIYVAVAVMGVTMETHKSGNEVKVVIKGLHESQQEELLELVQRA<br>AELAGVRVRIRFKGDTVTIVVRGLEHHHHHH
C3-A-L267S	SDEAIKRLLEELRASTAELKRATASLRAITEELKKNPSEDALVEHNRAIVEHNA<br>IIVENNRIIAAVLEAIVAEFILAKARLLVWEARKKGNPEEVLEAAKKALRVAEEA<br>AKAGIEAIFKAAAEAALEIAMRLIDLATKKGDPDLVREAQKVAERVAELADKN<br>GDEEVKLKAYAAAVAAEIIRRIETLKRSGSSYEDIRQTLRETLERIIEELKRRGV<br>DSSQIVWAIIYVAVAVMGVTMETHKSGNEVKVVIKGLHESQQEELLELVSRA<br>AELAGVRVRIRFKGDTVTIVVRGLEHHHHHH

C3-A_nHalo	MAEIGTGFPFDPHYVEVLGERMHYVDVGPRDGTPVLFLHGNPTSSYVWRNII<br>PHVAPTHRCIAPDLIGMGKSDKPDLGYFFDDHVRFMDAFIEALGLEEVVLVIH<br>DWGSALGFHWAKRNPERVKGIAFMEFIRPIPTWDEWPEFARETFQAFRTTD<br>VGRKLIIDQNVFIEGTLPMGVVRPLTEVEMDHYREPFLNPVDREPLWRFPNE<br>LPIAGEPANIVALVEEYMDWLHQSPVPKLLFWGTPGVLIPPAEAARLAKSLPN<br>CKAVDIGPGLNLLQEDNPDLIGSEIARWLSTLEISG<br>GSGSGSGSGSGSGSGSGSGS<br>SDEAIKRLLEELRASTAELKRATASLRAITEELKKNPSEDALVEHNRAIVEHNA<br>IIVENNRIIAAVLEAIVAEFILAKARLLVWEARKKGNPEEVLEAAKKALRVAEEA<br>AKAGIEAIFKAAAEAALEIAMRLIDLATKKGDPDLVREAQKVAERVAELADKN<br>GDEEVKLKAYAAAVAAEIIRRIETLKRSGSSYEDIRQTLRETLERIIEELKRRGV<br>DSSQIVWAIIYVAVAVMGVTMETHKSGNEVKVVIKGLHESQQEELLELVLRAA<br>ELAGVRVRIRFKGDTVTIVVRGLEHHHHHH
C3-A_nSpyCatcher	GAMVDTLSGLSSEQGQSGDMTIEEDSATHIKFSKRDEDGKELAGATMELRD<br>SSGKTISTWISDGQVKDFYLYPGKYTFVETAAPDGYEVATAITFTVNEQGQV<br>TVNGKATKGDAHI<br>GSGSGSGSGSGSGS<br>SDEAIKRLLEELRASTAELKRATASLRAITEELKKNPSEDALVEHNRAIVEHNA<br>IIVENNRIIAAVLEAIVAEFILAKARLLVWEARKKGNPEEVLEAAKKALRVAEEA<br>AKAGIEAIFKAAAEAALEIAMRLIDLATKKGDPDLVREAQKVAERVAELADKN<br>GDEEVKLKAYAAAVAAEIIRRIETLKRSGSSYEDIRQTLRETLERIIEELKRRGV<br>DSSQIVWAIIYVAVAVMGVTMETHKSGNEVKVVIKGLHESQQEELLELVLRAA<br>ELAGVRVRIRFKGDTVTIVVRGLEHHHHHH
C3-A_nSpyTag	AHIVMVDAYKPTKGSGSGSGSGSGSGSSDEAIKRLLEELRASTAELKRATAS<br>LRAITEELKKNPSEDALVEHNRAIVEHNAIIVENNRIIAAVLEAIVAEFILAKARLL<br>VWEARKKGNPEEVLEAAKKALRVAEEAAKAGIEAIFKAAAEAALEIAMRLIDL<br>ATKKGDPDLVREAQKVAERVAELADKNGDEEVKLKAYAAAVAAEIIRRIETLK<br>RSGSSYEDIRQTLRETLERIIEELKRRGVDSSQIVWAIIYVAVAVMGVTMETH<br>KSGNEVKVVIKGLHESQQEELLELVLRAAELAGVRVRIRFKGDTVTIVVRGLE<br>HHHHHH
C2-B-α40 (T~1)	MLRIEIETENEDMPAVEAMLAALSKKVKEVEVTIRAPEWDEEGVKHLAKAYE<br>ALAKNAEYAKLTVEGLHI<br>KQQRQLYRDVRETSKKQGVETEIEVEGDTVTIVVREGSWSGLEHHHHHH

C2-B-α30 (T1~3)	LRIEIEGPREEVVATLRAIAAAEAETNPKFAAILNTVADFLARSPLEEVSVRIERTPDELVITLEGLSPKQQRQLYRDVRETSKKQGVETEIEVEGDTVTIVVREGSWSGLEHHHHHH
C2-B-α20 (T~3)	PTLRVEIEGPAADVAALLRGVAELAAERAPKLAPVVAVIADFVASRPGPVRVRVEMGDGVLRVVLEGLHIKQQRQLYRDVRETSKKQGVETEIEVEGDTVTIVVREGSWSGLEHHHHHH
C2-B-α13 (T~9)	MGEVRLDVRVDIDAPTEEDAAAVMRAIMEGIVAAAKAVKEIGLRFRLDLTLRDATAAELRELVRMIEELVKIGAEVRVVVEGLSPKQQRQLYRDVRETSKKQGVETEIEVEGDTVTIVVREGSWSGLEHHHHHH
C2-B-α10 (T~16)	ATVHVTIRGDLAAADVAALAEAVMREVGRIGARLELTLDLPADITPEDLRRIVEALMECAKKTGLARLTLRGLSPKQQRQLYRDVRETSKKQGVETEIEVEGDTVTIVVREGSWSGLEHHHHHH
C2-B-α8 (T~25)	MKGTFVQEGPIPASKATTAVKKLTELAKEVGFDELDVTLQLSSDNTEEELKELAKALVELQLAVGAKITLVGVSPKQQRQLYRDVRETSKKQGVETEIEVEGDTVTIVVREGSWSGLEHHHHHH
C2-B-α8-2 (T~25)	MSELNITLKNLPDEVALDIVKEVCKSLIKHGVKAKVNITLEGLSVETIIQIYKDLIKLSKETGSEVTLTVKGLHIKQQRQLYRDVRETSKKQGVETEIEVEGDTVTIVVREGSWSGLEHHHHHH
C2-B-α8-3 (T~25)	MKKIKVKAEASTVEELKILCDALKAIVKAALKENEKLQAVAETILDFVKSLGKPLSIELVIEDNKIEITIKGLHIKQQRQLYRDVRETSKKQGVETEIEVEGDTVTIVVREGSWSGLEHHHHHH
C2-B-α20_nMCherry	MVSKGEEDNMAIIKEFMRFKVHMEGSVNGHEFEIEGEGEGRPYEGTQTAKLKVTKGGPLPFAWDILSPQFMYGSKAYVKHPADIPDYLKLSFPEGFNWERVMNFEDGGVVTVTQDSSLQDGEFIYKVKLRGTNFPSDGPVMQCRTMGWEASTERMYPEDGALKGEIKQRLKLKDGGHYDAEVKTTYKAKKPVQLPGAYNVDIKLDILSHNEDYTIVEQYERAEGRHSTGGMDELYKGGSGGSGGSGGSGGSPTLRVEIEGPAADVAALLRGVAELAAERAPKLAPVVAVIADFVASRPGPVRVRVEMGDGVLRVVLEGLHILEHHHHHH
C2-B-α10_nMCherry	MVSKGEEDNMAIIKEFMRFKVHMEGSVNGHEFEIEGEGEGRPYEGTQTAKLKVTKGGPLPFAWDILSPQFMYGSKAYVKHPADIPDYLKLSFPEGFNWERVMNFEDGGVVTVTQDSSLQDGEFIYKVKLRGTNFPSDGPVMQCRTMGWEAST

	ERMYPEDGALKGEIKQRLKLKDGGHYDAEVKTTYKAKKPVQLPGAYNVDIKL DILSHNEDYTIVEQYERAEGRHSTGGMDELYK<br>GGSGGSGGSGGSGGS<br>ATVHVTIRGDLAAADVAALAEAVMREVGRIGARLELTLDLPADITPEDLRRIVE ALMECAKKTGLARLTLRGLSPKQQRQLYRDVRETSKKQGVETEIEVEGDTV TIVVREGSWSGLEHHHHHH
C2-B-α8_nMCherry	MVSKGEEDNMAIIKEFMRFKVHMEGSVNGHEFEIEGEGEGRPYEGTQTAKL KVTKGGPLPFAWDILSPQFMYGSKAYVKHPADIPDYLKLSFPEGFNWERVM NFEDGGVVTVTQDSSLQDGEFIYKVKLRGTNFPSDGPVMQCRTMGWEAST ERMYPEDGALKGEIKQRLKLKDGGHYDAEVKTTYKAKKPVQLPGAYNVDIKL DILSHNEDYTIVEQYERAEGRHSTGGMDELYKGGSGGSGGSGGSGGSMKG TFVQEGPIPASKATTAVKKLTELAKEVGFDELDVTLQLSSDNTEEELKELAKA LVELQLAVGAKITLVGVSPKQQRQLYRDVRETSKKQGVETEIEVEGDTVTIVV RELEHHHHHH
C2-B B4-Cdynorphin A-binder	MKGTFVQEGPIPASKATTAVKKLTELAKEVGFDELDVTLQLSSDNTEEELKEL AKALVELQLAVGAKITLVGVSPKQQRQLYRDVRETSKKQGVETEIEVEGDTV TIVVRE<br>GGSGGSGGSGGSGGS<br>MSPEERELAERNLREARKNGSPLVPELERLKELSPLQAAALVNLAVAELRGN ERAEELARRAKEVADEDPLRAAALINAAAAELDGNPLARELVRAVEEDAHPL VRAALINLAVAELDGREDPDLRRVIEATRKGTITDEEAREVIERAYREGSWSG LEHHHHHH
C2-B-α8_cAPOE3	KVEQAVETEPEPELRQQTEWQSGQRWELALGRFWDYLRWVQTLSEQVQE ELLSSQVTQELRALMDETMKELKAYKSELEEQLTPVAEETRARLSKELQAAQ ARLGADMEDVCGRLVQYRGEVQAMLGQSTEELRVRLASHLRKLRKRLLRD ADDLQKRLAVYQAGAREGAERGLSAIRERLGPLVEQGRVRAATVGSLAGQP LQERAQAWGERLRARMEEMGSRTRDRLDEVKEQVAEVRAKLEEQAQQIRL QAEAFQARLKSWFEPLVEDMQRQWAGLVEKVQAAVGTSAAPVPSDNHGS GSGSGSGSGSGSGSMKGTFVQEGPIPASKATTAVKKLTELAKEVGFDELDV TLQLSSDNTEEELKELAKALVELQLAVGAKITLVGVSPKQQRQLYRDVRETS KKQGVETEIEVEGDTVTIVVRELEHHHHHH
C2-B-α20_nGFPnb	PTLRVEIEGPAADVAALLRGVAELAAERAPKLAPVVAVIADFVASRPGPVRVR VEMGDGVLRVVLEGLHIKQQRQLYRDVRETSKKQGVETEIEVEGDTVTIVVR EGGSGGSGGSGGSGGSVQLVESGGALVQPGGSLRLSCAASGFPVNRYSM RWYRQAPGKEREWVAGMSSAGDRSSYEDSVKGRFTISRDDARNTVYLQM NSLKPEDTAVYYCNVNVGFEYWGQGTQVTVSSLEHHHHHH

C2-B-α20_nGFP_cSortilin-ET	MHHHHHHGSSKGEELFTGVVPILVELDGDVNGHKFSVRGEGEGDATNGKLTLKFICTTGKLPVPWPTLVTTLTYGVQCFARYPDHMKQHDFFKSAMPEGYVQERTISFKDDGTYKTRAEVKFEGDTLVNRIELKGIDFKEDGNILGHKLEYNFNSHNVYITADKQKNGIKANFKIRHNVEDGSVQLADHYQQNTPIGDGPVLLPDNHYLSTQSVLSKDPNEKRDHMVLLEFVTAAGITHGMDELYGGSGGSGGSGGSGGSPTLRVEIEGPAADVAALLRGVAELAAERAPKLAPVVAVIADFVASRPGPVRVRVEMGDGVLRVVLEGLHIKQQRQLYRDVRETSKKQGVETEIEVEGDTVTIVVREGGSGGSGGSGGSGGSSFLLKHAVRVIERLLEEGNVELAEKWVEQLKRLAHAQRDPEALRRAEELEEIVEEL
C2-B-α10_nGFP_cSortilin-ET	MHHHHHHGSSKGEELFTGVVPILVELDGDVNGHKFSVRGEGEGDATNGKLTLKFICTTGKLPVPWPTLVTTLTYGVQCFARYPDHMKQHDFFKSAMPEGYVQERTISFKDDGTYKTRAEVKFEGDTLVNRIELKGIDFKEDGNILGHKLEYNFNSHNVYITADKQKNGIKANFKIRHNVEDGSVQLADHYQQNTPIGDGPVLLPDNHYLSTQSVLSKDPNEKRDHMVLLEFVTAAGITHGMDELYGGSGGSGGSGGSGGSATVHVTIRGDLAAADVAALAEAVMREVGRIGARLELTLDLPADITPEDLRRIVEALMECAKKTGLARLTLRGLSPKQQRQLYRDVRETSKKQGVETEIEVEGDTVTIVVREGGSGGSGGSGGSGGSSFLLKHAVRVIERLLEEGNVELAEKWVEQLKRLAHAQRDPEALRRAEELEEIVEEL
C2-B-α8_nGFP_cSortilin-ET	MHHHHHHGSSKGEELFTGVVPILVELDGDVNGHKFSVRGEGEGDATNGKLTLKFICTTGKLPVPWPTLVTTLTYGVQCFARYPDHMKQHDFFKSAMPEGYVQERTISFKDDGTYKTRAEVKFEGDTLVNRIELKGIDFKEDGNILGHKLEYNFNSHNVYITADKQKNGIKANFKIRHNVEDGSVQLADHYQQNTPIGDGPVLLPDNHYLSTQSVLSKDPNEKRDHMVLLEFVTAAGITHGMDELYGGSGGSGGSGGSGGSMKGTFVQEGPIPASKATTAVKKLTELAKEVGFDELDVTLQLSSDNTEEELKELAKALVELQLAVGAKITLVGVSPKQQRQLYRDVRETSKKQGVETEIEVEGDTVTIVVREGGSGGSGGSGGSGGSSFLLKHAVRVIERLLEEGNVELAEKWVEQLKRLAHAQRDPEALRRAEELEEIVEEL
C2-B-α20_nGFP_cIGF2R-ET	MHHHHHHGSSKGEELFTGVVPILVELDGDVNGHKFSVRGEGEGDATNGKLTLKFICTTGKLPVPWPTLVTTLTYGVQCFARYPDHMKQHDFFKSAMPEGYVQERTISFKDDGTYKTRAEVKFEGDTLVNRIELKGIDFKEDGNILGHKLEYNFNSHNVYITADKQKNGIKANFKIRHNVEDGSVQLADHYQQNTPIGDGPVLLPDNHYLSTQSVLSKDPNEKRDHMVLLEFVTAAGITHGMDELYGGSGGSGGSGGSGGSPTLRVEIEGPAADVAALLRGVAELAAERAPKLAPVVAVIADFVASRPGPVRVRVEMGDGVLRVVLEGLHIKQQRQLYRDVRETSKKQGVETEIEVEGDTVTIVVREGGSGGSGGSGGSGGSMVDARLLLLIWEAKELLERGNPEEARKVLEEAREIAERNNNEELLKELEVLLEKLEGGSTERRVIQVLEQILEDEDPEVVEKMLEILLEILEEAGDPARKLVEEILRVVRKNLEEARELVRRLS

C2-B-α20_nGFP_cASGPR-ET	MHHHHHHGSSKGEELFTGVVPILVELDGDVNGHKFSVRGEGEGDATNGKLTLKFICTTGKLPVPWPTLVTTLTYGVQCFARYPDHMKQHDFFKSAMPEGYVQERTISFKDDGTYKTRAEVKFEGDTLVNRIELKGIDFKEDGNILGHKLEYNFNSHNVYITADKQKNGIKANFKIRHNVEDGSVQLADHYQQNTPIGDGPVLLPDNHYLSTQSVLSKDPNEKRDHMVLLEFVTAAGITHGMDELYGGSGGSGGSGGSGGSPTLRVEIEGPAADVAALLRGVAELAAERAPKLAPVVAVIADFVASRPGPVRVRVEMGDGVLRVVLEGLHIKQQRQLYRDVRETSKKQGVETEIEVEGDTVTIVVREGGSGGSGGSGGSGGSAAEEAIRRLKELNTPEAEDLLDFIETLLWKGRPEEEALRKAAALARRWGEPEAARVIEEALKSLS
C2-B-α10_nGFP_cASGPR-ET	MHHHHHHGSSKGEELFTGVVPILVELDGDVNGHKFSVRGEGEGDATNGKLTLKFICTTGKLPVPWPTLVTTLTYGVQCFARYPDHMKQHDFFKSAMPEGYVQERTISFKDDGTYKTRAEVKFEGDTLVNRIELKGIDFKEDGNILGHKLEYNFNSHNVYITADKQKNGIKANFKIRHNVEDGSVQLADHYQQNTPIGDGPVLLPDNHYLSTQSVLSKDPNEKRDHMVLLEFVTAAGITHGMDELYGGSGGSGGSGGSGGSATVHVTIRGDLAAADVAALAEAVMREVGRIGARLELTLDLPADITPEDLRRIVEALMECAKKTGLARLTLRGLSPKQQRQLYRDVRETSKKQGVETEIEVEGDTVTIVVREGGSGGSGGSGGSGGSAAEEAIRRLKELNTPEAEDLLDFIETLLWKGRPEEEALRKAAALARRWGEPEAARVIEEALKSLS
C2-B-α8_nGFP_cASGPR-ET	MHHHHHHGSSKGEELFTGVVPILVELDGDVNGHKFSVRGEGEGDATNGKLTLKFICTTGKLPVPWPTLVTTLTYGVQCFARYPDHMKQHDFFKSAMPEGYVQERTISFKDDGTYKTRAEVKFEGDTLVNRIELKGIDFKEDGNILGHKLEYNFNSHNVYITADKQKNGIKANFKIRHNVEDGSVQLADHYQQNTPIGDGPVLLPDNHYLSTQSVLSKDPNEKRDHMVLLEFVTAAGITHGMDELYGGSGGSGGSGGSGGSMKGTFVQEGPIPASKATTAVKKLTELAKEVGFDELDVTLQLSSDNTEEELKELAKALVELQLAVGAKITLVGVSPKQQRQLYRDVRETSKKQGVETEIEVEGDTVTIVVREGGSGGSGGSGGSGGSAAEEAIRRLKELNTPEAEDLLDFIETLLWKGRPEEEALRKAAALARRWGEPEAARVIEEALKSLS

References

60. Sigl, C. et al. Programmable icosahedral shell system for virus trapping. Nat. Mater. **20**, 1281–1289 (2021).

61. Sutter, M., Greber, B., Aussignargues, C. & Kerfeld, C. A. Assembly principles and structure of a 6.5-MDa bacterial microcompartment shell. Science **356**, 1293–1297 (2017).

62. Praetorius, F. et al. Design of stimulus-responsive two-state hinge proteins. Science **381**, 754–760 (2023).

63. Pillai, A. et al. De novo design of allosterically switchable protein assemblies. Nature **632**, 911–920 (2024).

64. Bausch, A. R. et al. Grain Boundary Scars and Spherical Crystallography. Science **299**, 1716–1718 (2003).

65. Lipowsky, P., Bowick, M. J., Meinke, J. H., Nelson, D. R. & Bausch, A. R. Direct visualization of dislocation dynamics in grain-boundary scars. Nat. Mater. **4**, 407–411 (2005).

66. Bowick, M. J. & Giomi, L. Two-Dimensional Matter: Order, Curvature and Defects. https://doi.org/10.48550/ARXIV.0812.3064 (2008) doi:10.48550/ARXIV.0812.3064.

67. Liu, Z. B., Halldorsson, S., Calcraft, T., Calder, L. J. & Rosenthal, P. B. Topological defects drive influenza glycoprotein lattice assembly on spherical membranes. Preprint at https://doi.org/10.1101/2025.11.06.686978 (2025).

68. Irvine, W. T. M., Vitelli, V. & Chaikin, P. M. Pleats in crystals on curved surfaces. Nature **468**, 947–951 (2010).

69. Tanjeem, N., Wilkin, W. H., Beller, D. A., Rycroft, C. H. & Manoharan, V. N. Geometrical Frustration and Defect Formation in Growth of Colloidal Nanoparticle Crystals on a Cylinder: Implications for Assembly of Chiral Nanomaterials. ACS Appl. Nano Mater. **4**, 10682–10691 (2021).

70. Lidmar, J., Mirny, L. & Nelson, D. R. Virus shapes and buckling transitions in spherical shells. Phys. Rev. E **68**, 051910 (2003).
